# The identification of economically relevant health and social care services for mental disorders in the PECUNIA project

**DOI:** 10.1186/s12913-023-09944-0

**Published:** 2023-09-29

**Authors:** Paul Hinck, Mencia Gutierrez-Colosía, Christine Duval, Hans-Helmut König, Judit Simon, Claudia Fischer, Susanne Mayer, Luis Salvador-Carulla, Valentin Brodszky, Leona Hakkaart-van Roijen, Silvia Evers, A.-La Park, William Hollingworth, on behalf of the PECUNIA Group, Michael Berger, Agata Łaszewska, Nataša Peri´c, Christian Brettschneider, Marie Christine Duval, Johanna Katharina Hohls, Louisa-Kristin Muntendorf, László Gulácsi, Silvia M. A. A. Evers, Ruben M. W. A. Drost, Luca M. M. Janssen, Aggie T. G. Paulus, Irina Pokhilenko, Kimberley Hubens, Ayesha Sajjad, Pedro Serrano-Aguilar, Nerea Almeda, Pilar Campoy-Muñoz, Carlos R. García-Alonso, Mencía R. Gutiérrez-Colosía, Cristina Romero-López-Alberca, Lidia García-Pérez, Renata Linertová, Lilisbeth Perestelo-Pérez, Cristina Valcárcel-Nazco, Sian Noble, Joanna Thorn, Alexander Konnopka

**Affiliations:** 1 Department of Health Economics and Health Services Research, University Medical Center Hamburg-Eppendorf (UKE), Martinistr. 52, 20251 Hamburg, Germany; 2 Department of Psychology, Universidad Loyola Andalucía, Seville, Spain; 3 Scientific Association PSICOST, Seville, Spain; 4 Department of Health Economics, Center for Public Health, Medical University of Vienna (MUW), Vienna, Austria; 5 Department of Psychiatry, University of Oxford, Warneford Hospital, Oxford, UK; 6 Health Research Institute, Faculty of Health, University of Canberra, Canberra, Australia; 7 Menzies Centre for Health Policy. School of Population Health, Faculty of Medicine and Health, University of Sydney, Camperdown, NSW Australia; 8 Department of Health Economics, Corvinus University of Budapest (CUB), Budapest, Hungary; 9 Institute for Medical Technology Assessment, Erasmus University Rotterdam (EUR), Burgemeester Oudlaan 50, PO Box 1738, 3000 Rotterdam, DR Netherlands; 10 Health Technology Assessment, Erasmus School of Health Policy and Management, Erasmus University Rotterdam (EUR), Burgemeester Oudlaan 50, PO Box 1738, 3000 Rotterdam, DR Netherlands; 11 Department of Health Services Research, Care and Public Health Research Institute (CAPHRI), Faculty of Health, Medicine and Life Sciences (FHML), Maastricht University (UM), Maastricht, the Netherlands; 12 Trimbos, Netherlands Institute of Mental Health and Addiction, Da Costakade 45, 3521 VS Utrecht, The Netherlands; 13 Care Policy and Evaluation Centre (CPEC), Department of Health Policy, London School of Economics and Political Science (LSE), London, UK; 14 Health Economics Bristol, Department of Population Health Sciences, Bristol Medical School (UnivBris), Bristol, UK

**Keywords:** Economic evaluation, Service identification, Mental disorders, Resource use measurement

## Abstract

**Background:**

Health economic research is still facing significant problems regarding the standardization and international comparability of health care services. As a result, comparative effectiveness studies and cost-effectiveness analyses are often not comparable. This study is part of the PECUNIA project, which aimed to improve the comparability of economic evaluations by developing instruments for the internationally standardized measurement and valuation of health care services for mental disorders. The aim of this study was to identify internationally relevant services in the health and social care sectors relevant for health economic studies for mental disorders.

**Methods:**

A systematic literature review on cost-of-illness studies and economic evaluations was conducted to identify relevant services, complemented by an additional grey literature search and a search of resource use measurement (RUM) questionnaires. A preliminary long-list of identified services was explored and reduced to a short-list by multiple consolidation rounds within the international research team and an external international expert survey in six European countries.

**Results:**

After duplicate removal, the systematic search yielded 15,218 hits. From these 295 potential services could be identified. The grey literature search led to 368 and the RUM search to 36 additional potential services. The consolidation process resulted in a preliminary list of 186 health and social care services which underwent an external expert survey. A final consolidation step led to a basic list of 56 services grouped into residential care, daycare, outpatient care, information for care, accessibility to care, and self-help and voluntary care.

**Conclusions:**

The initial literature searches led to an extensive number of potential service items for health and social care. Many of these items turned out to be procedures, interventions or providing professionals rather than services and were removed from further analysis. The resulting list was used as a basis for typological coding, the development of RUM questionnaires and corresponding unit costs for international mental health economic studies in the PECUNIA project.

**Supplementary Information:**

The online version contains supplementary material available at 10.1186/s12913-023-09944-0.

## Background

Health economic evaluations are an integral part of health technology assessments and can inform policymakers and facilitate decision-making processes about the costs and the effectiveness of different health interventions. Cost-effectiveness analyses (CEAs) take changes in costs and the effectiveness of two or more alternative interventions into account, and are consequently of particular importance for decision-making within and across countries. Therefore, the need for standardization of economic evaluations to enable proper decision-making and transferability of results has long been recognized in the health economics community [1]. However, the reporting standards do still differ substantially. Health economic research is still facing significant problems regarding the standardization and international comparability of the definitions of services [2, 3], the measurement of service utilization [4], and the monetary valuation of services [5–7]. There is a demand for comparable health service data to improve evidence-based decision-making in health policy. The health agencies of the European Union (EU) need commensurable descriptions of care to evaluate health equity and ensure mobility of health service users in the EU [2]. Currently, the comparison of services and their contents is difficult, even if they have identical names, due to terminological variability [8]. The definition of a service is not always clear and can differ substantially. Even the term ‘service’ itself can refer to a range of elements such as the provider, a combination of functions, programs, resources, the facility of service provision or an organizational unit [2]. A service may be organized differently on an international, federal, regional, or even communal level, and it can also be challenging to define the target population of a service. There is a recognized shortcoming of international agreement on classification systems. In particular, there are no uniform coding matrices for different settings, which may lead to biased results in health services research [9, 10].

Another problem for the comparability of health economic studies is the lack of standardized resource use measurement (RUM) instruments, which may lead to unnecessary fluctuations of economic evaluation data [4, 11]. This impedes the comparability, transferability, and applicability of these studies within and across countries [12, 13]. RUMs are considered an important method for measuring the resources used by health service users within economic evaluations. Nevertheless, existing RUMs are often country-specific [14] or population-specific [15]. Hence, there is a need for harmonized RUMs to improve the overall methodology of both cost-effectiveness and comparative effectiveness studies [11, 13] in terms of international comparability. According to Mayer et al. [16], the first step in a RUM development process is the identification of economically relevant items by means of a literature review.

Likewise, there is also a substantial degree of variation in the applied valuation methods in European health economic studies [17]. For example, the micro-costing method and the gross costing method not only differ in their complexity but also their resulting estimates [5]. This lack of standardization potentially complicates the comparability and transferability of economic evaluations. The general methodological credibility of monetary valuations is hence questionable, and economic evaluations may be less useful for decision-making [18]. The underlying reason is that the applied valuation method has a significant impact on the results [19].

The EU project “ProgrammE in Costing, resource use measurement and outcome valuation for Use in multi-sectoral National and International health economic evaluAtions” (PECUNIA) aims to overcome the problems described above by developing standardized multi-sectoral, multi-national and multi-person resource use measurement instruments, standardized unit costs, and outcome assessment tools to improve the methodology of economic evaluations in the European Union [13, 20–24]. The PECUNIA Consortium coordinated by the Medical University of Vienna consists of ten institutions situated in six European countries: Austria (AT), Germany (DE), Hungary (HU), the Netherlands (NL), Spain (ES), and the United Kingdom (UK) [25]. Three mental disorders: depression, schizophrenia and post-traumatic stress disorder (PTSD) were chosen as exemplary diseases due to their high disease burden and economic relevance. The project is exploring multiple economic sectors and domains, namely “health and social care” sectors, “education and (criminal) justice” sectors, “employment and productivity” domains, and “patient, family and informal care” domains.

The aim of this study was to identify services in the health and social care sectors that are relevant for the calculation of costs in economic evaluations from a societal perspective. According to the “whole systems approach”, the inclusion of the social care sector is pivotal in providing comprehensive mental health care. In particular, community-based social care interventions might be neglected in some healthcare systems, while they can have a significant impact on the course of a mental disorders [26]. Hence, social care services were also explicitly considered in this study to achieve a cross-sectoral broad perspective.

## Methods

This study included two major steps: step I) a systematic and grey literature review for data identification, data extraction and the production of a preliminary listing of terms following initial consolidation steps (“preliminary service list”); step II) final consolidation step following external expert surveys in all six PECUNIA countries for the production of a “basic service list” used in all further steps in the PECUNIA project. These steps involved two working groups (A and B) as well as internal and external expert panels. The initial identification and consolidation steps included working group A from UKE (PH, AK, CD, HHK) and working group B from MUW (JS, CF, SM) supported by the relevant country-lead PECUNIA partners for country-specific tasks (internal expert panel: AT/MUW, DE/UKE, HU/CUB, NL/UM & EUR, ES/Psicost, UK/LSE & UnivBris). The external expert panel composed of 29 health and social service researchers, health economists, and planners from public agencies and other stakeholders in every participating country (AT: 7, DE: 3, HU: 4, NL: 5, ES: 6, UK: 4).

**Fig. 1:**
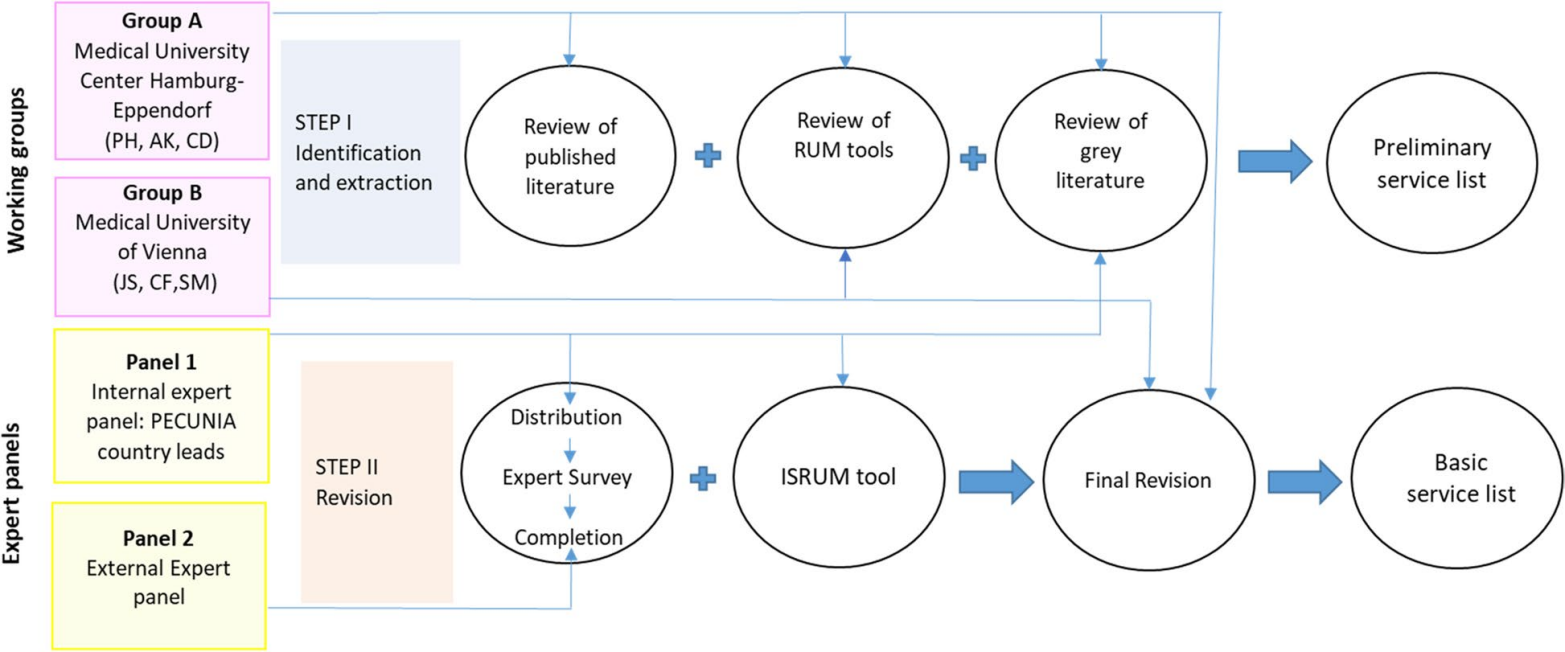
Process of the identification of relevant health and social care services in the PECUNIA project

Step I was led by working group A and involved working group B as well as the internal expert panel. Step II was led by working group B and involved working group A, the internal expert panel and the external expert panel. The whole process and the activities performed by the working groups and the expert panels are shown in Fig. 1.

### Step I – Identification and extraction

Firstly, working group A carried out a systematic review of the peer-reviewed literature to identify relevant services in mental health and social care. A systematic search strategy was applied including the use of indexed vocabulary (e.g. MeSH terms) and other keywords, combined with different Boolean operators and limits (details are shown Appendix 1). The structure of this systematic review was based on the PRISMA checklist. Information was gathered by screening of the databases PUBMED-Medline, PsycINFO, NHS EED, EconLit and Embase. One combined search was conducted for the health care and social care sectors. To identify relevant studies, we used a two-step approach. In the first step, titles and where necessary abstracts were screened to exclude studies that were distinct irrelevant. In the second step, all studies identified as possibly relevant in step one were obtained and screened for eligibility in full text.

Eligibility criteria were defined prior to the literature search. To identify relevant service items from a costing perspective, the systematic literature review included cost-of-illness studies and economic evaluations focusing on mental disorders. Publications in Dutch, English, German, Hungarian, and Spanish as per the involved countries from the previous ten years (search conducted in May 2018) were considered. Exclusion criteria included editorials, letters, case reports and reviews or cost-of-illness studies and cost-effectiveness analyses not focusing on mental disorders. Additionally, studies based on results already published elsewhere and abstracts without full-text availability were excluded. Working group A screened the methods and results sections of included studies to identify relevant service items, which were extracted into a MsExcel® file together with information on item definition (if available) and country of origin. In line with the more specific research focus of the PECUNIA project, the systematic literature search focused on services relevant for mental disorders. Although the PECUNIA project focusses on depression, schizophrenia and PTSD as exemplary diseases, we made no restrictions to specific diseases in this study for two reasons. On the one hand, we wanted to assure that we get a full picture of services relevant to mental disorders (i.e. although we choose three exemplary diseases to work with in the PECUNIA project, we developed further tools like a resource use questionnaire for mental disorders in general). On the other hand, it seemed impracticable to assign services to specific disorders, hence a full search for services was seen as the appropriate strategy.

Secondly, a manual search for additional key papers and national grey literature was performed by the internal expert panel in AT, DE, HU, NL, ES, and the UK. This focused on selected mental disorders including documents on care for depression, schizophrenia and PTSD, covering clinical guidelines, PhD-theses and publications by the government or major organizations in each of the six participating countries. No limit regarding the timeframe was set for the grey literature search, but only the most recent versions of e.g. clinical guidelines were included. To ensure a standardized approach, researchers were provided with identical search instructions and an extraction template for service items. The template asked researchers to name identified services used by persons with mental disorders together with a short description in English and national language and to provide information regarding disease, treatment setting as well as type of data source and references.

Thirdly, as part of the grey literature search, working group A screened the content of RUM questionnaires from the DIRUM repository, RUM questionnaires identified in studies included in step I, and at a later stage the so-called “Core items for a standardized resource-use measure (ISRUM)” list from the UK [27]. Since the PECUNIA project aimed to develop a generic RUM questionnaire for use in economic evaluations, RUM screening was not limited to services specific for mental disorders only.

All identified items were added to a preliminary service list in an MsExcel® file which then underwent multiple consolidation steps. Working group A supported by working group B eliminated obvious duplicates and provided brief “working-definitions” for each remaining item. If not self-explanatory (e.g. “General Practitioner-contact”), these descriptions were either taken from the respective sources (systematic or grey literature search), or were newly developed based on an item-specific internet research. Descriptions were further face-validated by members of the internal expert panel before exploited in the external expert survey.

### Step II – External expert survey and final revision

Working group B led the distribution of the consolidated preliminary service list as an MsExcel® based survey to the external expert panel in each of the six participating countries organized by the PECUNIA country leads. The survey was carried out between November 2018 and January 2019 and included questions on the clarity of definitions, the relevance and the economic impact of the identified items, and a request for any additional relevant missed services for the respective national health and social care sectors. Further details on the survey and resulting country reports can be found elsewhere [28].

The results of the expert survey were synthetized and consolidated jointly by working groups A and B with input from authors MGC and LSC for preparing a “basic service list” for all further work steps in the PECUNIA project. At this stage, eligible items from the ISRUM list [27] were added to the preliminary service list. Services were defined as follows [2, 10]:

*“A ‘service’ is a micro-level functional system of care organization, defined as the smallest unit with own administrative structure available within the catchment area (micro-organization).”*

Consequently, working groups A and B excluded all items that did not match this definition, in particular interventions (e.g. “cognitive behavioral therapy”), tests (e.g. “neuropsychological examination”) and professions (e.g. “GP assistant”). This step was necessary to reduce double counting and as a preparatory step for the DESDE coding (for details please see [29]) adapted to PECUNIA to define service-typologies for the development of internationally comparable units of analysis, harmonized resource use measurement, and corresponding reference unit costs for international (mental) health economic studies. Several of the resulting services were also merged into one single service due to similarity in terms of care activities and costs. For example, contacts with outpatient physicians and contacts with outpatient nurses were combined to one service “outpatient healthcare service” which was defined as a contact with an outpatient health care provider. On the other hand, some services were also split up, in particular if they were provided in more than one main branch of the DESDE coding-system. For example, “rehabilitation” may be provided as a residential or as an outpatient service resulting in two distinct services with distinctively different unit costs.

## Results

### Step I – Identification and extraction

**Fig. 2:**
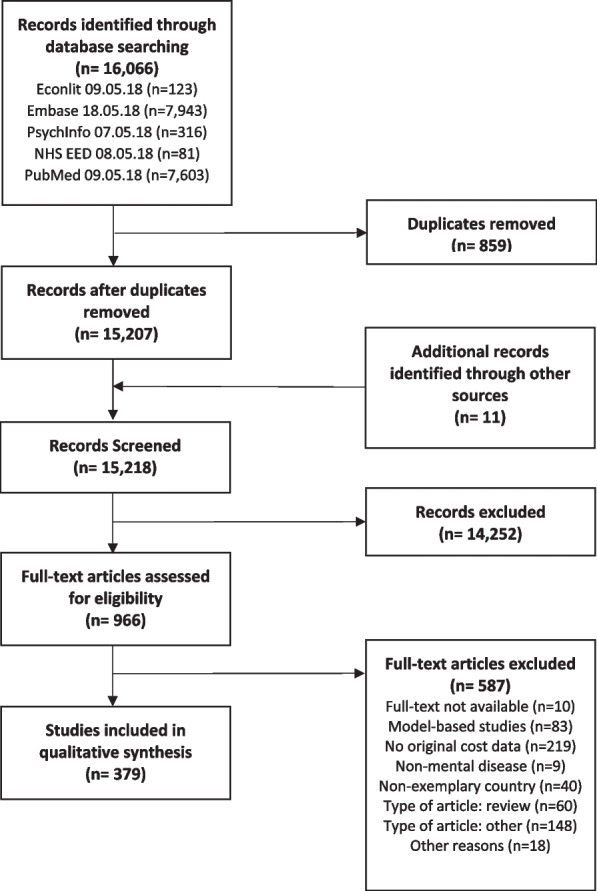
PRISMA flow chart of the systematic literature search

The systematic literature review yielded 16,066 hits, of which 859 were removed as duplicates (Fig. 2). After adding 11 studies from other sources, titles and abstracts of 15,218 records were screened for relevancy according to the eligibility criteria and 966 publications were assessed in full-text. Of these, 587 publications were excluded for reasons (for references see Appendix 3), such as reporting no original cost data, or being a non-eligible study type. Hence, 379 studies (for references see Appendix 2) were included in the qualitative synthesis of relevant potential service items. In addition to the systematic literature search, the national grey literature searches yielded an additional 81 relevant publications and the search for RUM questionnaires yielded 11 instruments that were screened for further service items (for details see Appendix 4).

**Fig. 3:**
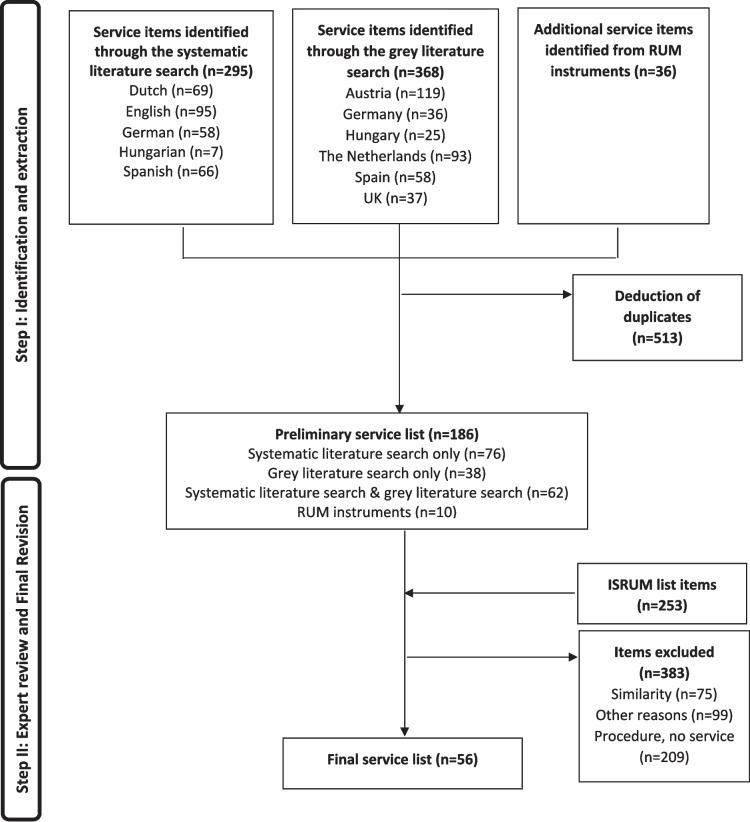
The process of identification and disambiguation of items

The total number of potential health and social care service items identified by the systematic literature search, the grey literature search and the RUM search is shown in Fig. 3. In this step, 699 items were identified. Whilst 95 of items identified in the systematic literature search, were published in international journals with English language, a considerable amount of 200 items were identified in publications from national sources. These items were translated into English language by the national partners together with the country-specific items identified in the grey literature search. Items were checked for duplicates, which were then excluded. The remaining 186 items constituted the preliminary service list used for the expert survey. The participating countries had the option to adjust the list to national specifics. For example, in Austria outpatient specialist services can be delivered in-hospital or out-of-hospital settings with distinctively different costs, hence both settings were asked as separate questions, which increased the number of items for the external expert survey in Austria.

### Step II – External expert survey and final revision

Due to the very high number of services (*n* = 186) stemming from different areas of care (health or social care) and including rare or highly specialized services, responses were heterogeneous between experts. Often experts answered that they did not know how relevant items were regarding the intensity of their utilization. No additional items were added to the preliminary service list, nor were items deleted based on the expert survey.

Final revision of the preliminary service list based on the expert survey results by working groups A and B with input from authors MGC and LSC resulted in the “basic service list” consisting of 56 identified health and social care services (Table 1).

**Table 1:** Basic service list of services in the health and social care sectors

Service	Definition
**Residential care**	
Psychiatric residential home	A mental health care facility providing sheltered and medically monitored living for mentally impaired individuals
Assisted living facility	A facility/housing that provides a place to live and medical care for people who require help with daily activities, that has been specially built for, or changed to a certain standard to accommodate people with disabilities
Residential home	A sanctuary for temporary housing, set up to provide for the needs of homeless people/women, often including shelter, food, sanitation and other forms of support
Assisted tenant group for mentally ill persons	A housing group that aids and assists people who require help with their daily activities due to mental illness
Sheltered housing for mentally ill persons	A sanctuary for temporary housing, set up to provide for the needs of homeless people/women with mental disorders, often including shelter, food, sanitation and other forms of support
Semi-residential social care	Night care (day at home)
Non-psychiatric hospital ward	A hospital unit others than mental disorders
Non-psychiatric intensive care ward	Areas of the Hospital where seriously ill patients receive specialized care such as intensive monitoring and advanced life support
Rehabilitation facility	A center or clinic where people recovering from illness, injury or addiction are treated
Hospice	An inpatient care facility that offers end-of-life care to terminally ill patients
Nursing home	An inpatient care facility that offers care for elderly or disabled persons
Acute psychiatric ward	A hospital unit specializing in the treatment of serious, acute mental disorders
Psychiatric intensive care unit (PICU)	A type of psychiatric inpatient ward, that is designed to care for patients who pose a risk to themselves or others
Psychiatric long-term ward	A hospital unit specializing in the long-term treatment of mental disorders
Soteria house	Alternative inpatient care for people who experience a psychotic crisis
Non-addiction related psychiatric rehabilitation	A center or clinic where people recovering from a mental illness others than addiction are treated
Addiction-related rehabilitation	A center or clinic where people recovering from addiction are treated
**Daycare**	
Day hospital	Stay at a hospital at day, be at home during night
Day care – voluntary organization	Care or supervision provided during the day for physically disabled persons by a voluntary organization
Day care – professional organization	Care or supervision provided during the day for physically disabled persons by a professional organization
Psychiatric daycare unit	A daycare-specific hospital unit providing specialized care for patients suffering from mental disorders
Social day-care	A social care facility providing support for individuals in need (day-care)
**Outpatient care**	
Polyclinic	A clinic that provides both general and specialist examinations and treatments
Outpatient healthcare service	A contact with an outpatient health care provider
Emergency doctor on call	An outpatient doctor coming with a car in case of emergency
Outpatient healthcare at the workplace	e.g. company physician, company nurse
Outpatient healthcare service at school	e.g. school physician, school nurse
Outpatient healthcare service at the prison	Ambulatory care that is offered to prison inmates
Pediatric outpatient clinic	A healthcare facility that offers care to children and adolescents in an outpatient setting
Hospital-based outpatient clinic	A department of the hospital that offers ambulatory care to outpatients
Psychiatric drop-in center	A daycentre run by social services or a charity that clients in mental distress may attend on an informal basis
Psychiatric rehabilitation aftercare service	Continuous care program for individuals that have been stabilized after mental illness and have left a rehabilitation center, designed to prevent relapse
Mental counselling center/ advice center	A center providing counselling support, advice and aid to individuals in mental distress
Long-term-care facility	A social care facility providing support for individuals in need (long-term care)
Child development centre	A facility that provides educational services to children and their families with the aid of age-appropriate programs
Social care outpatient service	A social care facility providing support for individuals in need
Psychosocial crisis center	A center focused on supporting individuals in emergencies and people exposed to extreme stressors, such as refugees, internally displaced persons, disaster survivors and terrorism-, war- or genocide-exposed populations
Social care facility for mentally ill persons	A social care facility providing support for individuals with mental disorders
Social assistance for mentally ill persons	Services provided by the government or private organizations to help mentally ill people
Psychological crisis resolution team	An immediate and short-term psychological-care-worker assisting individuals in a crisis situation in order to restore equilibrium to their biopsychosocial functioning and to minimize the potential of long-term psychological trauma
Legal carer/guardian for mentally ill persons	A person who has the legal authority to care for the personal and property interests of another individual
Integration workplace	A place of work promoting the integration and inclusion of disabled/mentally impaired people into a work environment
Vocational training	Individual qualification training for a specific type of job
Individual vocational qualification	Individual qualification training for a specific type of job
Supported employment programs	Programs for supporting people with significant disabilities to secure and retain paid employment
Integration services	A service that aids in integrating mentally impaired/ disabled individuals, or individuals absent from their job due to illness for a long period of time back into the workplace
Professional training	Individual training for a specific type of job and providing individuals with necessary knowledge to succeed
Protected/sheltered workshop for mentally ill persons	A place of work specially designed to provide a safe environment for mentally ill people
Pre-vocational assessment service for mentally ill persons	Support offered in terms of counseling, testing, etc. to individuals for career planning and placement in training programs
**Information for care**	
Hotline	A telephone hotline that offers information to patients
Support helplines	A special phone service offering advice and support to people in distress
**Accesability to care**	
Emergency ambulance ride	A special vehicle used to take sick or injured people to a hospital or other health care facility in case of emergency
General transport	A special vehicle used to take sick or injured people to a hospital or other health care facility as planed transport
Escort/accompanied leave	A person who travels with someone in order to protect, guide or guard them
**Self-help and voluntary care**	
Self-help groups	Voluntary associations of people who share a common desire to overcome mental illness or otherwise increase their level of cognitive or emotional wellbeing
Voluntary community support	Voluntary community support for individuals in need of aid

During the revision process, we excluded 383 out of 439 items from the combined preliminary service list and the ISRUM list items. Most items were excluded because they were procedures rather than services (*n* = 209). Procedures were excluded to avoid double counting with services including these procedures. For example, diagnostic tests (e.g. “TSH-test”, “neuropsychological examination”, etc.) were excluded as procedures that are performed in the context of an “outpatient healthcare contact”, which is the service of interest included into the basic service list. Further 75 items were excluded because of similarity. This means that different items were combined to one service in the basic service list. For example, the service “outpatient health care contact” comprised all different types of outpatient physician specializations (e.g. GP, psychotherapist, neurologist, etc.). Finally, another 99 items were excluded for “other reasons”. These were predominantly items from the ISRUM list, that did not represent items related to health or social care at all. For example, questions regarding income losses or travel costs or items belonging to other service areas like the (criminal) justice sector or the educational sector.

We grouped identified services according to the main branches of the DESDE system: “residential care” (*n* = 17), “outpatient care” (*n* = 27), “day care” (*n* = 5), “information for care” (*n* = 2), “accessibility to care” (*n* = 3), and “self-help and voluntary care” (*n* = 2).

Overall, outpatient care was the largest group containing about half of all identified services (*n* = 27). Only 10 out of the 27 services were explicitly for persons with mental disorders. Most outpatient care services could roughly be assigned to either outpatient medical care, outpatient social care or outpatient vocational assistance. Among those services assigned to outpatient medical care, four services were for persons with mental disorders (“psychiatric drop-in center”, “psychiatric rehabilitation aftercare service”, “mental counselling center/advice center”, “psychological crisis resolution team”). A further four services for persons with mental disorders could be assigned to outpatient social care (“psychosocial crisis center”, “social care facility for mentally ill persons”, “social assistance for mentally ill persons”, “legal carer/guardian for mentally ill persons”) and two services could be assigned to outpatient vocational assistance (“protected / sheltered workshop for mentally ill persons” and “pre-vocational assessment service for mentally ill persons”).

Out of the 18 services located in residential care, eight services were referring to living facilities, of which three were explicitly for persons with mental disorders (“psychiatric residential home”, “assisted tenant group for mentally ill persons” and “sheltered housing for mentally ill persons”). The remaining 10 services in residential care were treatment-related in either hospitals or rehabilitation units, of which six were explicitly for persons with mental disorders (“acute psychiatric ward”, “psychiatric intensive care unit (PICU)”, “psychiatric long-term ward”, “Soteria house”, “non-addiction related psychiatric rehabilitation”, “addiction-related rehabilitation”).

## Discussion

The objective of this study was to identify services relevant for persons with mental disorders in the health and social care sectors for economic analyses purposes. A provisional service list was compiled through systematic literature review and a grey literature search. Further data from existing RUM instruments were added. The preliminary service list resulting from these searches and several consolidation steps included 186 items, of which 56 were included in the final basic service list used for the development of the PECUNIA costing tools such as the PECUNIA Coding System [29], the PECUNIA RUM (Janssen LMMP, Evers I, Drost SMAA, Thorn RMWA, Hollingworth J, Noble W, et al: Involving respondents in the development process of the PECUNIA RUM instrument: Results of Think Aloud interviews, submitted) [30], the PECUNIA Reference Unit Costing (RUC) Templates [31] and the PECUNIA RUC Compendium [32, 33] https://www.pecunia-project.eu/tools).

The difference between the preliminary service list and the final basic service list resulted primarily from two reasons. First, we did not apply a specific definition of “service” when compiling the first preliminary service list. Instead, we collected all resource items related to health and social services use, in order to be as comprehensive as possible. As a result, the preliminary service list was a mix of not only services but also interventions, procedures or professions that had to be excluded subsequently. Second, for the further typology of services, the PECUNIA Consortium agreed to define a service according to the international DESDE classification system as adapted for the PECUNIA project. This allowed achieving a higher consolidation level where different items could be combined into one internationally harmonized service item for costing purposes. For example, the service “outpatient healthcare service” includes all types of outpatient physicians and non-physician healthcare providers.

This study allowed the realization of the magnitude of the problem when “words” are used to describe services for health economic research, because often “names” of services were prone to Babylonian confusion not allowing the comparison like with like. Whilst, for example, it was very clear for the national project partners and contact points what a simple service use like “mental hospital stay” meant in their respective country and/or region, a detailed analysis in terms of cost measurement for economic evaluations revealed that mental hospital stays may mean very different things in different countries. Affected dimensions were for example eligible target populations (regarding age group and gender, diagnosis, disease severity or functional impairment), treatments offered, average duration, setting (with and/or without overnight stay), and degree of specialization (mental ward versus closed mental ward). This problem has several implications. First, it points to a general limitation of comparisons of cost-of-illness studies and cost-effectiveness analyses when identically named services, do in fact not represent identical but rather similar compositions of utilized resources. Second, the description of services or utilized units in cost-of-illness studies and cost-effectiveness analysis can be questioned. Overall, studies rarely presented detailed descriptions of the units measured to calculate costs. We assume that this information is often regarded as unnecessary for evaluation purposes because the definition of services seems to be clear in the national context. While this might to be acceptable from a national perspective, it becomes a substantial problem, if one tries to compare the units of analysis internationally. However, if one tries to describe a service, the inherent question arises of how best to do this. Using words can introduce new problems by creating circular references. For these reasons, it was decided in the PECUNIA Consortium to use the DESDE coding system for the description of services.

As expected, most of the identified services were located in residential and outpatient facilities. Hence, currently these types of services seem to play a pivotal role in mental healthcare provision in Europe. Conversely, services in the remaining segments of care (day care, accessibility to care, information to care, self-help and voluntary care) are very much underrepresented. This might change to some extent due to care transitions induced by the COVID-19 pandemic like increased use of video-consultations that outlast the end of the pandemic. Nevertheless, we identified a large number of services beyond “medical care” for example services providing protected living, assistance in different areas of daily living or the workplace.

### Implications for future research

Our major learning concerns problems related to the use of language to describe health care services. Language turned out to be too vague and ambiguous to describe services in the international contexts. For this purpose, services were described using a taxonomical coding system in a next step. The question remains, how this problem should be addressed in further cost-of-illness studies and cost-effectiveness analyses. As already discussed, these studies rarely provided definitions of the items measured for cost calculation. Based on our findings this presents an underestimated source for inter-study heterogeneity. PECUNIA provides tools that can help to reduce this problem, but a certain amount of fuzziness will remain in international service comparisons, resulting from the between-country differences in the services themselves.

Future health services research should incorporate the analysis of vagueness and ambiguity of the terms, the production of international glossaries of terms after disambiguation of the identified terms, and dictionaries to translate the international terms into local terminology to improve semantic interoperability. In particular, cost-effectiveness and comparative effectiveness research should compare the same units of analysis and use a common vocabulary. This might increase the validity of multinational studies significantly, but healthcare provision can often also differ within a country on a regional or local level. Currently, the majority of studies compare solely the name of a service, but not the exact elements of the service such as the target group, type of care, activities, and type(s) and the number of professionals involved. The application of such a classification system is, therefore, particularly useful when developing new measurement instruments.

This study was used as a basis for the development of the PECUNIA RUM instrument. RUMs are important tools for measuring resource use in economic evaluations and their development process should follow a stepwise approach. A literature review should be conducted to identify economically and clinically relevant services for the specified disease area or target population. In addition, an expert survey may be conducted to evaluate the importance of the identified services and rank them accordingly. Existing RUMs can also be identified and analyzed regarding their content, structure and layout [12]. The results of this study reveal relevant services provided to health service users with mental disorders in the health and social care sector internationally, at least within the European context. This research was the first step in the process of the PECUNIA RUM development, i.e. the identification of economically relevant services for mental healthcare provision with a focus on Europe. The multinational PECUNIA RUM instrument is also harmonized with comparable reference unit costs (RUCs) in different countries, as it offers a catalogue of defined services that can be monetarily valued.

### Strengths and limitations

We followed a highly standardized and structured study approach and incorporated expertise from multiple international health economic and health services working groups. Nevertheless, there are several limitations to this study. First, the systematic and the grey literature searches led to a varying amount of publications per country. Hence, some countries may be overrepresented, while others may be underrepresented. On the other hand, since we checked all services in national expert surveys, the basic service list should be comprehensive for the participating countries and for generic services but may miss some specific services in health and social care of non-participating countries.

Second, the inclusion of grey literature may have negatively influenced the systematic approach of this review due to the lacking preset search strategy. Hence, the reproducibility of the results is limited [34]. In this study, there was even further variability in the search approach, because different research strategies may have been used in the national-level grey literature searches despite the centrally provided search instructions and extraction templates for service items. On the other hand, the inclusion of grey literature can also be seen as an advantage, because it can complement the data of a systematic review and ultimately increase the level of evidence. Additionally, the incorporation of grey literature may counteract potential publications bias [34].

Third, since the more specific research questions and the resources of the PECUNIA project were focused on mental disorders, we were not able to search specifically for non-mental health services with the same intensity as for services for mental health. We restricted the identification of non-mental health services to a screening of existing RUM questionnaires and included all the most common RUMs so it should be highly unlikely that we missed essential generic health and social care services.

Fourth, we did not double-screen for identification of studies and service items in the systematic literature review by two independent reviewers. This incorporates risk of falsely included or excluded studies, but the risk of bias resulting from this limitation should be negligible for several reasons: 1) we included a very high number of 379 studies limiting the risk of bias from missing out further relevant studies; 2) several researchers from different countries were involved in all steps related to the processing of identified variables, thus limiting the risk of bias resulting from false inclusion of studies or service items; 3) since we did not calculate metrics using study results, we were not prone to risk of bias related to issues of statistical calculations.

## Conclusion

We found an extensive variety of services provided to persons with mental disorders in Europe. It became apparent that the distinction between a service and a procedure is not always clear-cut. Hence, these discrepancies in the conceptualization of a service potentially impede their comparability in economic evaluations. In this study, economically relevant services for mental health and social care were identified and organized according to the different “main types of care” of the DESDE classification system. As a result, a basic list of services for mental healthcare provision in Europe emerged. The resulting service list provided the basis for a typology coding system of services for internationally comparable units of analysis, the development of a harmonized RUM instruments, and corresponding reference unit costs for international mental health economic studies within the PECUNIA project [30–33] (https://www.pecunia-project.eu/tools).

### Supplementary Information

**Additional file 1:** **Appendix 1.** Systematic search concept. **Appendix 2.** Included publications from systematic literature review. **Appendix 3.** Excluded publications from systematic literature review. **Appendix 4.** Identified and screened resource use questionnaires.

## Data Availability

The datasets used and/or analyzed during the current study are available from the corresponding author on reasonable request.

## Abbreviations

AT: Austria
DE: Germany
DESDE: Description and evaluation of services and directories in Europe
ES: Spain
HU: Hungary
ISRUM: Core items for a standardized resource-use measure
NL: Netherlands
RUM: Resource use measurement (instrument)
UK: United Kingdom
