# Supplementary material for: The identification of economically relevant health and social care services for mental disorders in the PECUNIA project
Source: BMC Health Serv Res. 2023 Sep 29;23:1045. doi: 10.1186/s12913-023-09944-0 (PMC10542258; doi:10.1186/s12913-023-09944-0)
Supplement: Supplementary file 1 — Additional file 1: Appendix 1. Systematic search concept. Appendix 2. Included publications from systematic literature review. Appendix 3. Excluded publications from systematic literature review. Appendix 4. Identified and screened resource use questionnaires. [file 12913_2023_9944_MOESM1_ESM.docx]

## Appendix 1: Systematic search concept

| # | **Key concept** | **Search terms** |
| --- | --- | --- |
| #1 | Mental Health component | [“Mental Disorders” [Mesh] OR mental OR dement* OR Alzheimer OR “cognitive impairment” OR addiction OR "substance abuse" OR "dependency" OR psychotic* OR schizophreni* OR delusional OR manic OR affective OR bipolar OR depressi* OR "depressive disorder" OR anxiety OR panic OR phobia OR "posttraumatic stress disorder*" OR "somatoform disorder" OR somatiz* OR somatis* OR "conversion disorder" OR “dissociative disorder” OR multisomatoform* OR neurotic* OR "medically unexplained symptoms" OR "medically unexplained illness" OR psychogen* OR nonorgan* OR (psychosom* syndrome) OR "functional somatic syndrome" OR "functional syndrome" OR hypochondri* OR "illness phobia" OR "health anxiety" OR "body dysmorphic disorder" OR dysmorphophobia OR "anorexia nervosa" OR "bulimia nervosa" OR binge* OR purging OR "eating disorder*" OR "personality disorder*" OR schizoid OR schizot* OR "behavioural disorder*" OR "behavioral disorder*” OR "obsessive-compulsive disorder*" OR “impulse disorder*” OR “gender identity disorder*” OR “intellectual disabilit*” OR “developmental disorder*” OR “attention-deficit disorder*” OR “attention-deficit hyperactivity disorder*”] |
| #1* |  | “Mental Disorders” [Mesh] |
| #2 | Economic component | [“cost-effectiveness” OR “cost-utility” OR “cost-benefit” OR "economic evaluation" OR cost] |
| #3 | Country component | [german* OR austria* OR hungar* OR Netherlands OR dutch OR spain OR spanish OR United Kingdom] |

Limits to: past 10 years, humans, German, Hungarian, Dutch, Spanish and English

*in PUBMED-Medline, we only used the Mesh term “Mental disorders”, since use of the full search string (#1) would have led to unmanageable amount of search results

## Appendix 2: Included publications from systematic literature review

1. Aardoom, J.J., et al., *Cost-utility of an internet-based intervention with or without therapist support in comparison with a waiting list for individuals with eating disorder symptoms: a randomized controlled trial.* Int J Eat Disord, 2016. **49**(12): p. 1068-1076.

2. Acarturk, C., et al., *Economic costs of social phobia: A population-based study.* Journal of Affective Disorders, 2009. **115**(3): p. 421-429.

3. Altmann, U., et al., *Outpatient psychotherapy reduces health-care costs: A study of 22,294 insurants over 5 years.* Frontiers in Psychiatry, 2016. **7**(JUN).

4. Amner, K., *The effect of DBT provision in reducing the cost of adults displaying the symptoms of BPD.* British Journal of Psychotherapy, 2012. **28**(3): p. 336-352.

5. Aragones, E., et al., *Cost-effectiveness analysis of a collaborative care programme for depression in primary care.* Journal of Affective Disorders, 2014. **159**: p. 85-93.

6. Arends, I., et al., *Economic evaluation of a problem solving intervention to prevent recurrent sickness absence in workers with common mental disorders.* PLoS One, 2013. **8**(8): p. e71937.

7. Avramenko, A., et al., *Cost of care of patients with personality disorders in forensic psychiatric hospitals in the Netherlands.* Criminal Behaviour and Mental Health, 2009. **19**(3): p. 165-177.

8. Baca-Garcia, E., et al., *Patterns of mental health service utilization in a general hospital and outpatient mental health facilities: Analysis of 365,262 psychiatric consultations.* European Archives of Psychiatry and Clinical Neuroscience, 2008. **258**(2): p. 117-123.

9. Bamelis, L.L., et al., *Economic evaluation of schema therapy and clarification-oriented psychotherapy for personality disorders: A multicenter, randomized controlled trial.* The Journal of Clinical Psychiatry, 2015. **76**(11): p. e1432-e1440.

10. Banerjee, S., Hellier, J., Romeo, R., Dewey, M., Knapp, M., et al. , *Study of the use of antidepressants for depression in dementia: the HTA -SADD trial - a multicentre, randomised, double-blind, placebo-controlled trial of the clinical effectiveness and cost-effectiveness of sertraline and mirtazapine.* Health Technology Assessment 2013. **17**(7).

11. Banerjee, S. and R. Wittenberg, *Clinical and cost effectiveness of services for early diagnosis and intervention in dementia.* Int J Geriatr Psychiatry, 2009. **24**(7): p. 748-54.

12. Barnes, T.R.E., et al., *Amisulpride augmentation in clozapine-unresponsive schizophrenia (AMICUS): A double-blind, placebo-controlled, randomised trial of clinical effectiveness and cost-effectiveness.* Health Technology Assessment, 2017. **21**(49): p. i-53.

13. Barrett, B., et al., *The assessment of dangerous and severe personality disorder: Service use, cost, and consequences.* Journal of Forensic Psychiatry and Psychology, 2009. **20**(1): p. 120-131.

14. Barrett, B., et al., *Service and wider societal costs of very young children with autism in the UK.* Journal of Autism and Developmental Disorders, 2012. **42**(5): p. 797-804.

15. Barrett, B., et al., *Comparing service use and costs among adolescents with autism spectrum disorders, special needs and typical development.* Autism, 2015. **19**(5): p. 562-569.

16. Barton GR, H.J., Mugford M, Jones PB, Croudace T, Fowler D. , *Cognitive behaviour therapy for improving social recovery in psychosis: cost-effectiveness analysis.* Schizophr Res, 2009. **112**(1-3): p. 158-163.

17. Bendeck, M., et al., *An integrative cross-design synthesis approach to estimate the cost of illness: an applied case to the cost of depression in Catalonia.* J Ment Health, 2013. **22**(2): p. 135-54.

18. Benecke, C., et al., *A comparison of psychoanalytic therapy and cognitive behavioral therapy for anxiety (Panic/agoraphobia) and personality disorders (APD study): Presentation of the RCT study design.* Zeitschrift fur Psychosomatische Medizin und Psychotherapie, 2016. **62**(3): p. 252-269.

19. Benedict, Á., et al., *Economic evaluation of duloxetine versus serotonin selective reuptake inhibitors and venlafaxine XR in treating major depressive disorder in Scotland.* Journal of Affective Disorders, 2010. **120**(1-3): p. 94-104.

20. Berghout, C.C., J. Zevalkink, and L. Hakkaart-Van Roijen, *The effects of long-term psychoanalytic treatment on healthcare utilization and work impairment and their associated costs.* Journal of Psychiatric Practice, 2010. **16**(4): p. 209-216.

21. Berghout, C.C., J. Zevalkink, and L. Hakkaart-van Roijen, *A cost-utility analysis of psychoanalysis versus psychoanalytic psychotherapy.* Int J Technol Assess Health Care, 2010. **26**(1): p. 3-10.

22. Biesheuvel-Leliefeld, K.E., et al., *Cost-effectiveness of nurse-led self-help for recurrent depression in the primary care setting: design of a pragmatic randomised controlled trial.* BMC Psychiatry, 2012. **12**: p. 59.

23. Blankers, M., et al., *Economic evaluation of internet-based interventions for harmful alcohol use alongside a pragmatic randomized controlled trial.* Journal of Medical Internet Research, 2012. **14**(5): p. 71-83.

24. Bock, J.-O., et al., *Excess health care costs of late-life depression-Results of the AgeMooDe study.* Journal of Affective Disorders, 2016. **199**: p. 139-147.

25. Bock, J.-O., et al., *The impact of depressive symptoms on healthcare costs in late life: Longitudinal findings from the AgeMooDe study.* The American Journal of Geriatric Psychiatry, 2017. **25**(2): p. 131-141.

26. Bodden D H M, D.C.D., Bogels S M, Nauta M H, De Haan E, Ringrose J, Appelboom C, Brinkman A G, Appelboom-Geerts K C, *Costs and cost-effectiveness of family CBT versus individual CBT in clinically anxious children.* Clinical Child Psychology and Psychiatry, 2008. **13**(4): p. 543-564.

27. Bodden, D.H.M., C.D. Dirksen, and S.M. Bögels, *Societal burden of clinically anxious youth referred for treatment: A cost-of-illness study.* Journal of Abnormal Child Psychology, 2008. **36**(4): p. 487-497.

28. Bode, K., et al., *Population-based cost-offset analyses for disorder-specific treatment of anorexia nervosa and bulimia nervosa in Germany.* International Journal of Eating Disorders, 2017. **50**(3): p. 239-249.

29. Bode, K., et al., *Health Care Costs of Borderline Personality Disorder and Matched Controls with Major Depressive Disorder: A Comparative Study Based on Anonymized Claims Data.* European Journal of Health Economics, 2017. **18**(9): p. 1125-1135.

30. Boege, I., et al., *Cost-effectiveness of intensive home treatment enhanced by inpatient treatment elements in child and adolescent psychiatry in Germany: A randomised trial.* European Psychiatry, 2015. **30**(5): p. 583-589.

31. Borschmann, R., et al., *Joint crisis plans for people with borderline personality disorder: Feasibility and outcomes in a randomised controlled trial.* British Journal of Psychiatry, 2013. **202**(5): p. 357-364.

32. Bosanquet, K., et al., *Collaborative care for screen-positive elders with major depression (CASPER plus): A multicentred randomized controlled trial of clinical effectiveness and cost-effectiveness.* Health Technology Assessment, 2017. **21**(67): p. 1-251.

33. Bosmans, J.E., et al., *Health care costs of depression in primary care patients in The Netherlands.* Family Practice, 2010. **27**(5): p. 542-548.

34. Bosmans, J.E., et al., *Cost-effectiveness of a stepped care programme to prevent depression and anxiety in residents in homes for the older people: A randomised controlled trial.* International Journal of Geriatric Psychiatry, 2014. **29**(2): p. 182-190.

35. Bosmans, J.E., et al., *Cost-effectiveness of usual general practitioner care with or without antidepressant medication for patients with minor or mild-major depression.* Journal of Affective Disorders, 2008. **111**(1): p. 106-112.

36. Bosmans, J.E., et al., *Cost-effectiveness of problem-solving treatment in comparison with usual care for primary care patients with mental health problems: a randomized trial.* BMC Fam Pract, 2012. **13**: p. 98.

37. Brabyn, S., et al., *The second Randomised Evaluation of the Effectiveness, cost-effectiveness and Acceptability of Computerised Therapy (REEACT-2) trial: does the provision of telephone support enhance the effectiveness of computer-delivered cognitive behaviour therapy? A randomised controlled trial.* Health Technol Assess, 2016. **20**(89): p. 1-64.

38. Braun, S., et al., *Treatment Costs of Attention Deficit Hyperactivity Disorder in Germany.* European Journal of Health Economics, 2013. **14**(6): p. 939-945.

39. Brimblecombe, N., et al., *The role of youth mental health services in the treatment of young people with serious mental illness: 2-year outcomes and economic implications.* Early Intervention in Psychiatry, 2017. **11**(5): p. 393-400.

40. Buntrock, C., et al., *Preventing Depression in Adults With Subthreshold Depression: Health-Economic Evaluation Alongside a Pragmatic Randomized Controlled Trial of a Web-Based Intervention.* J Med Internet Res, 2017. **19**(1): p. e5.

41. Buszewicz, M., et al., *Pilot of a randomised controlled trial of the selective serotonin reuptake inhibitor sertraline versus cognitive behavioural therapy for anxiety symptoms in people with generalised anxiety disorder who have failed to respond to low-intensity psychological treatments as defined by the national institute for health and care excellence guidelines.* Health Technology Assessment, 2017. **21**(45): p. 1-137.

42. Butler, A., et al., *Frailty: a costly phenomenon in caring for elders with cognitive impairment.* Int J Geriatr Psychiatry, 2016. **31**(2): p. 161-8.

43. Byford, S., et al., *Cost-effectiveness of injectable opioid treatment v. oral methadone for chronic heroin addiction.* Br J Psychiatry, 2013. **203**(5): p. 341-9.

44. Caballero-Martínez, F., et al., *Use of health care resources and loss of productivity in patients with depressive disorders seen in Primary Care: INTERDEP Study.* Actas espanolas de psiquiatria, 2014. **42**(6): p. 281-291.

45. Carrasco, J.L., et al., *A comparative cost-analysis of initiating pregabalin or SSRI/SNRI therapy in benzodiazepine-resistant patients with generalized anxiety disorder in Spain.* Actas Espanolas de Psiquiatria, 2013. **41**(3): p. 164-174.

46. Cary, M., et al., *Economic evaluation of multisystemic therapy for young people at risk for continuing criminal activity in the UK.* PLoS One, 2013. **8**(4): p. e61070.

47. Cary, M., et al., *Human trafficking and severe mental illness: an economic analysis of survivors' use of psychiatric services.* BMC health services research, 2016. **16**: p. 284.

48. Chalder, M., et al., *A pragmatic randomised controlled trial to evaluate the cost-effectiveness of a physical activity intervention as a treatment for depression: The treating depression with physical activity (TREAD) trial.* Health Technology Assessment, 2012. **16**(10): p. i-xii+1-164.

49. Charlesworth, G., et al., *Does befriending by trained lay workers improve psychological well-being and quality of life for carers of people with dementia, and at what cost? A randomised controlled trial.* Health Technology Assessment, 2008. **12**(4): p. 1-48.

50. Chernyak, N., et al., *Economic evaluation of brief psychodynamic interpersonal therapy in patients with multisomatoform disorder.* PLoS One, 2014. **9**(1): p. e83894.

51. Chollet, J., et al., *A clinical research practice datalink analysis of antidepressant treatment patterns and health care costs in generalized anxiety disorder.* Value in Health, 2013. **16**(8): p. 1133-1139.

52. Chorozoglou, M., et al., *Preschool hyperactivity is associated with long-term economic burden: evidence from a longitudinal health economic analysis of costs incurred across childhood, adolescence and young adulthood.* Journal of child psychology and psychiatry, and allied disciplines, 2015. **56**(9): p. 966-975.

53. Coduras, A., et al., *Prospective one-year cost-of-illness study in a cohort of patients with dementia of Alzheimer's disease type in Spain: The ECO study.* Journal of Alzheimer's Disease, 2010. **19**(2): p. 601-615.

54. Collins, B.J., K. Cuddy, and A.P. Martin, *Assessing the effectiveness and cost-effectiveness of drug intervention programs: UK case study.* J Addict Dis, 2017. **36**(1): p. 5-13.

55. Coulton, S., et al., *Effectiveness and cost-effectiveness of community singing on mental health-related quality of life of older people: Randomised controlled trial.* British Journal of Psychiatry, 2015. **207**(3): p. 250-255.

56. Craig TKJ, J.S., McCrone P, Afuwape S, Hughes E, Gournay K, White I, Wanigaratne S, Leese M, Thornicroft G, *Integrated care for co-occurring disorders: psychiatric symptoms, social functioning, and service costs at 18 months. .* Psychiatr Serv 2008. **59**(3): p. 276-282.

57. Crawford, M.J., et al., *Group art therapy as an adjunctive treatment for people with schizophrenia: a randomised controlled trial (MATISSE).* Health Technol Assess, 2012. **16**(8): p. iii-iv, 1-76.

58. Crawford, M.J., et al., *Lamotrigine for people with borderline personality disorder: A RCT.* Health Technology Assessment, 2018. **22**(17).

59. Creswell, C., et al., *Treatment of childhood anxiety disorder in the context of maternal anxiety disorder: A randomised controlled trial and economic analysis.* Health Technology Assessment, 2015. **19**(38): p. 1-218.

60. Cuijpers, P., et al., *Economic costs of neuroticism: A population-based study.* Archives of General Psychiatry, 2010. **67**(10): p. 1086-1093.

61. D'Amico, F., et al., *Use of services and associated costs for young adults with childhood hyperactivity/conduct problems: 20-Year follow-up.* British Journal of Psychiatry, 2014. **204**(6): p. 441-447.

62. D'Amico, F., et al., *Cost-effectiveness of exercise as a therapy for behavioural and psychological symptoms of dementia within the EVIDEM-E randomised controlled trial.* International Journal of Geriatric Psychiatry, 2016. **31**(6): p. 656-665.

63. Dams, J., et al., *Excess costs of social anxiety disorder in Germany.* Journal of Affective Disorders, 2017. **213**: p. 23-29.

64. Davidson, K.M., et al., *Cognitive therapy v. usual treatment for borderline personality disorder: Prospective 6-year follow-up.* British Journal of Psychiatry, 2010. **197**(6): p. 456-462.

65. Davies, L.M., et al., *A randomized controlled trial of the cost-utility of second-generation antipsychotics in people with psychosis and eligible for clozapine.* Value in Health, 2008. **11**(4): p. 549-562.

66. De Bruin, E.J., F.J. van Steensel, and A.M. Meijer, *Cost-Effectiveness of Group and Internet Cognitive Behavioral Therapy for Insomnia in Adolescents: Results from a Randomized Controlled Trial.* Sleep, 2016. **39**(8): p. 1571-81.

67. De Salas-Cansado, M., et al., *Pregabalin versus SSRIs and SNRIs in benzodiazepine-refractory outpatients with generalized anxiety disorder: A post hoc cost-effectiveness analysis in usual medical practice in Spain.* ClinicoEconomics and Outcomes Research, 2012. **4**(1): p. 157-168.

68. De Salas-Cansado M, A.E., Olivares JM, Carrasco JL, Ferro MB, Rejas J, *Modelling the cost-effectiveness of pregabalin versus usual care in daily practice in the treatment of refractory generalised anxiety disorder.* Social Psychiatry and Psychiatric Epidemiology, 2013. **48**(6): p. 985-996.

69. Dodel, I., et al., *Cost of illness in patients with Gilles de la Tourette's syndrome.* J Neurol, 2010. **257**(7): p. 1055-61.

70. Dodel, R., et al., *Determinants of societal costs in Alzheimer's disease: GERAS study baseline results.* Alzheimer's & Dementia: The Journal of the Alzheimer's Association, 2015. **11**(8): p. 933-945.

71. Drost, R.M., et al., *A Web-Based Computer-Tailored Alcohol Prevention Program for Adolescents: Cost-Effectiveness and Intersectoral Costs and Benefits.* J Med Internet Res, 2016. **18**(4): p. e93.

72. Drummond, C., et al., *Effectiveness and cost-effectiveness of a stepped care intervention for alcohol use disorders in primary care: pilot study.* Br J Psychiatry, 2009. **195**(5): p. 448-56.

73. Duarte, A., et al., *Cost-effectiveness of computerized cognitive-behavioural therapy for the treatment of depression in primary care: findings from the Randomised Evaluation of the Effectiveness and Acceptability of Computerised Therapy (REEACT) trial.* Psychol Med, 2017. **47**(10): p. 1825-1835.

74. Edwards, R.T., et al., *Cost-effectiveness of a national exercise referral programme for primary care patients in Wales: results of a randomised controlled trial.* BMC Public Health, 2013. **13**: p. 1021.

75. Effertz, T., F. Verheyen, and R. Linder, *The costs of hazardous alcohol consumption in Germany.* Eur J Health Econ, 2017. **18**(6): p. 703-713.

76. Effertz, T., F.J. Verheyen, and R. Linder, *Economic and intangible costs of cannabis consumption in Germany.* Sucht: Zeitschrift fur Wissenschaft und Praxis, 2016. **62**(1): p. 31-41.

77. Egger, N., et al., *Long-term cost-effectiveness of cognitive behavioral therapy versus psychodynamic therapy in social anxiety disorder.* Depression and Anxiety, 2016. **33**(12): p. 1114-1122.

78. Egger, N., et al., *Short-term cost-effectiveness of psychodynamic therapy and cognitive-behavioral therapy in social anxiety disorder: Results from the SOPHO-NET trial.* Journal of Affective Disorders, 2015. **180**: p. 21-28.

79. Egger, N., et al., *Cost-effectiveness of focal psychodynamic therapy and enhanced cognitive-behavioural therapy in out-patients with anorexia nervosa.* Psychological medicine, 2016. **46**(16): p. 3291-3301.

80. Ekers D, G.C., Gilbody S, Parrott S, Richards DA, Hammond D, et al. , *Cost utility of behavioural activation delivered by the non-specialist. .* Br J Psychiatry. , 2011. **199**(6): p. 510-511.

81. Ersek, K., et al., *Costs of dementia in Hungary.* J Nutr Health Aging, 2010. **14**(8): p. 633-9.

82. Farre, M., et al., *Direct and indirect costs and resource use in dementia care: A cross-sectional study in patients living at home.* International Journal of Nursing Studies, 2016. **55**: p. 39-49.

83. Feenstra, D.J., et al., *The burden of disease among adolescents with personality pathology: quality of life and costs.* Journal of personality disorders, 2012. **26**(4): p. 593-604.

84. Felce, D., et al., *Cognitive behavioural anger management intervention for people with intellectual disabilities: Costs of intervention and impact on health and social care resource use.* Journal of Intellectual Disability Research, 2015. **59**(1): p. 68-81.

85. Felce, D.J., et al., *Outcomes and costs of community living: Semi-independent living and fully staffed group homes.* American Journal on Mental Retardation, 2008. **113**(2): p. 87-101+156.

86. François, C., et al., *Analysis of health-related quality of life and costs based on a randomised clinical trial of escitalopram for relapse prevention in patients with generalised social anxiety disorder.* International Journal of Clinical Practice, 2008. **62**(11): p. 1693-1702.

87. Frey, S., *The economic burden of schizophrenia in Germany: A population-based retrospective cohort study using genetic matching.* European Psychiatry, 2014. **29**(8): p. 479-489.

88. Gensichen, J., et al., *Cost-effectiveness of depression case management in small practices.* Br J Psychiatry, 2013. **202**: p. 441-6.

89. Geraedts, A.S., et al., *Economic Evaluation of a Web-Based Guided Self-Help Intervention for Employees With Depressive Symptoms: Results of a Randomized Controlled Trial.* J Occup Environ Med, 2015. **57**(6): p. 666-75.

90. Gerhards SA, d.G.L., Jacobs LE, Severens JL, Huibers MJ, Arntz A, Riper H, Widdershoven G, Metsemakers JF, Evers SM, *Economie evaluation of online computerised cognitive-behavioural therapy without support for depression in primary care: randomised.* British Journal of Psychiatry, 2010. **196**(4): p. 310-318.

91. Gilbert, H., et al., *Start2quit: a randomised clinical controlled trial to evaluate the effectiveness and cost-effectiveness of using personal tailored risk information and taster sessions to increase the uptake of the NHS Stop Smoking Services.* Health Technol Assess, 2017. **21**(3): p. 1-206.

92. Gilden, J., et al., *Does Treatment Adherence Therapy reduce expense of healthcare use in patients with psychotic disorders? Cost-minimization analysis in a randomized controlled trial.* Schizophr Res, 2011. **133**(1-3): p. 47-53.

93. Gillespie, P., et al., *The effects of dependence and function on costs of care for Alzheimer's disease and mild cognitive impairment in Ireland.* International Journal of Geriatric Psychiatry, 2013. **28**(3): p. 256-264.

94. Gonzalez-Pinto, A., et al., *In-patient care costs of patients with bipolar 1 disorder: A comparison between two European centers.* Journal of Affective Disorders, 2010. **121**(1-2): p. 152-155.

95. Goodyer, I.M., et al., *A randomised controlled trial of cognitive behaviour therapy in adolescents with major depression treated by selective serotonin reuptake inhibitors. The ADAPT trial.* Health Technology Assessment, 2008. **12**(14): p. iii-60.

96. Goodyer, I.M., et al., *Cognitive behavioural therapy and short-term psychoanalytical psychotherapy versus a brief psychosocial intervention in adolescents with unipolar major depressive disorder (IMPACT): a multicentre, pragmatic, observer-blind, randomised controlled superiority trial.* The Lancet Psychiatry, 2017. **4**(2): p. 109-119.

97. Goodyer, I.M., et al., *Cognitive-behavioural therapy and short-term psychoanalytic psychotherapy versus brief psychosocial intervention in adolescents with unipolar major depression (IMPACT): a multicentre, pragmatic, observer-blind, randomised controlled trial.* Health Technol Assess, 2017. **21**(12): p. 1-94.

98. Goorden, M., et al., *Cost-utility of collaborative care for major depressive disorder in primary care in the Netherlands.* Journal of Psychosomatic Research, 2015. **79**(4): p. 316-323.

99. Goorden, M., et al., *Cost utility analysis of a collaborative stepped care intervention for panic and generalized anxiety disorders in primary care.* Journal of Psychosomatic Research, 2014. **77**(1): p. 57-63.

100. Goorden, M., et al., *Cost-utility of collaborative care for the treatment of comorbid major depressive disorder in outpatients with chronic physical conditions. A randomized controlled trial in the general hospital setting (CC-DIM).* Neuropsychiatric Disease and Treatment, 2017. **13**: p. 1881-1893.

101. Goorden, M., et al., *Cost-effectiveness of multidimensional family therapy compared to cognitive behavioral therapy for adolescents with a cannabis use disorder: Data from a randomized controlled trial.* Drug and Alcohol Dependence, 2016. **162**: p. 154-161.

102. Goorden, M., et al., *Cost-utility analysis of a collaborative care intervention for major depressive disorder in an occupational healthcare setting.* J Occup Rehabil, 2014. **24**(3): p. 555-62.

103. Goranitis, I., et al., *Maximizing health or sufficient capability in economic evaluation? A methodological experiment of treatment for drug addiction.* Medical Decision Making, 2017. **37**(5): p. 498-511.

104. Gowers, S.G., et al., *A randomised controlled multicentre trial of treatments for adolescent anorexia nervosa including assessment of cost-effectiveness and patient acceptability - The TOuCAN trial.* Health Technology Assessment, 2010. **14**(15): p. 1-98.

105. Grabe, H.J., et al., *Association of mental distress with health care utilization and costs: A 5-year observation in a general population.* Social Psychiatry and Psychiatric Epidemiology, 2009. **44**(10): p. 835-844.

106. Graff MJ, A.E., Vernooij-Dassen MJ, Dekker J, Jonsson L, Thijssen M, Hoefnagels WH, Olde Rikkert MG, *Community occupational therapy for older patients with dementia and their care givers: cost effectiveness study.* BMJ, 2008. **336**(7636): p. 134-138.

107. Grande, I., et al., *Asenapine prescribing patterns in the treatment of manic in- and outpatients: Results from the MANACOR study.* European Psychiatry, 2015. **30**(4): p. 528-534.

108. Green, C., et al., *Cost-effectiveness of collaborative care for depression in UK primary care: economic evaluation of a randomised controlled trial (CADET).* PLoS One, 2014. **9**(8): p. e104225.

109. Green, J.M., et al., *Group therapy for adolescents with repeated self harm: randomised controlled trial with economic evaluation.* BMJ (Clinical research ed.), 2011. **342**.

110. Greven, P., et al., *Comparative treatment patterns, healthcare resource utilization and costs of atomoxetine and long-acting methylphenidate among children and adolescents with attention-deficit/hyperactivity disorder in Germany.* European Journal of Health Economics, 2017. **18**(7): p. 893-904.

111. Grupp, H., et al., *Excess costs from functional somatic syndromes in Germany — An analysis using entropy balancing.* Journal of Psychosomatic Research, 2017. **97**: p. 52-57.

112. Grupp, H., H.H. Konig, and A. Konnopka, *[Calculation of Standardised Unit Costs for the Economic Evaluation of Mental Disorders].* Gesundheitswesen, 2017. **79**(1): p. 48-57.

113. Gustavsson, A., et al., *Predictors of costs of care in Alzheimer's disease: A multinational sample of 1222 patients.* Alzheimer's & Dementia: The Journal of the Alzheimer's Association, 2011. **7**(3): p. 318-327.

114. Gustavsson, A., et al., *Cost of disorders of the brain in Europe 2010.* European Neuropsychopharmacology, 2011. **21**(10): p. 718-779.

115. Haas, L., et al., *The Trade-Off between Costs and Quality of Care in the Treatment of Psychosomatic Patients with Somatoform Pain Disorder.* Applied Health Economics and Health Policy, 2013. **11**(4): p. 359-368.

116. Hakkaart-van Roijen, L., et al., *Economic evaluation alongside a single RCT of an integrative psychotherapeutic nursing home programme.* BMC Health Serv Res, 2013. **13**: p. 370.

117. Hamre, H.J., et al., *Health costs in patients treated for depression, in patients with depressive symptoms treated for another chronic disorder, and in non-depressed patients: A two-year prospective cohort study in anthroposophic outpatient settings.* European Journal of Health Economics, 2010. **11**(1): p. 77-94.

118. Happich, M., et al., *Excess Costs Associated with Possible Misdiagnosis of Alzheimer's Disease Among Patients with Vascular Dementia in a UK CPRD Population.* J Alzheimers Dis, 2016. **53**(1): p. 171-83.

119. Heider, D., et al., *Direct medical mental health care costs of schizophrenia in France, Germany and the United Kingdom-Findings from the European Schizophrenia Cohort (EuroSC).* European Psychiatry, 2009. **24**(4): p. 216-224.

120. Heinrich, S., et al., *Accuracy of self-reports of mental health care utilization and calculated costs compared to hospital records.* Psychiatry Research, 2011. **185**(1-2): p. 261-268.

121. Henderson, C., et al., *Cost-effectiveness of telecare for people with social care needs: the Whole Systems Demonstrator cluster randomised trial.* Age Ageing, 2014. **43**(6): p. 794-800.

122. Henderson, C., et al., *Cost-Effectiveness of Financial Incentives to Promote Adherence to Depot Antipsychotic Medication: Economic Evaluation of a Cluster-Randomised Controlled Trial.* PLoS One, 2015. **10**(10): p. e0138816.

123. Hernando Ortiz, L., et al., *[Profitability of a day hospital: analysis of activity, cost and effectiveness].* Gac Sanit, 2012. **26**(4): p. 360-5.

124. Heslin, M., et al., *Randomised controlled trial to improve health and reduce substance use in established psychosis (IMPaCT): Cost-effectiveness of integrated psychosocial health promotion.* BMC Psychiatry, 2017. **17**(1).

125. Heuft, G., et al., *Normative-empirical determination of personnel requirements in psychosomatic medicine and psychotherapy.* Zeitschrift fur Psychosomatische Medizin und Psychotherapie, 2015. **61**(4): p. 384-398.

126. Hidalgo-Mazzei, D., et al., *The real world cost and health resource utilization associated to manic episodes: The MANACOR study.* Revista de Psiquiatria y Salud Mental, 2015. **8**(2): p. 55-64.

127. Hirjak, D., et al., *Evidence for distinguishable treatment costs among paranoid schizophrenia and schizoaffective disorder.* PLoS ONE, 2016. **11**(7).

128. Holden, S.E., et al., *The prevalence and incidence, resource use and financial costs of treating people with attention deficit/hyperactivity disorder (ADHD) in the united kingdom (1998 to 2010).* Child and Adolescent Psychiatry and Mental Health, 2013. **7**(1).

129. Hollinghurst, S., et al., *Cost-effectiveness of cognitive-behavioural therapy as an adjunct to pharmacotherapy for treatment-resistant depression in primary care: economic evaluation of the CoBalT Trial.* Br J Psychiatry, 2014. **204**(1): p. 69-76.

130. Hollinghurst S, P.T., Kaur S, Wiles N, Lewis G, Kessler D, *Cost-effectiveness of therapist- delivered online cognitive-behavioural therapy for depression: randomised controlled trial. .* Br J Psychiatry 2010. **197**: p. 297-304.

131. Holman, A.J., et al., *Cost-effectiveness of cognitive behaviour therapy versus talking and usual care for depressed older people in primary care.* BMC Health Serv Res, 2011. **11**: p. 33.

132. Hong, J., et al., *Clinical and economic consequences of medication non-adherence in the treatment of patients with a manic/mixed episode of bipolar disorder: Results from the European Mania in Bipolar Longitudinal Evaluation of Medication (EMBLEM) Study.* Psychiatry Research, 2011. **190**(1): p. 110-114.

133. Hong, J., et al., *The cost of relapse for patients with a manicmixed episode of bipolar disorder in the emblem study.* PharmacoEconomics, 2010. **28**(7): p. 555-566.

134. Hormigo Amaro, J., et al., *[Cost-benefit analysis of a school-based smoking prevention program].* Gac Sanit, 2009. **23**(4): p. 311-4.

135. Horn, E.K., et al., *Cost-Effectiveness of Short-Term Inpatient Psychotherapy Based on Transactional Analysis in Patients With Personality Disorder.* J Pers Disord, 2016. **30**(4): p. 483-501.

136. Hornberger, J., et al., *Cost-effectiveness of florbetapir-PET in Alzheimer's disease: A Spanish societal perspective.* Journal of Mental Health Policy and Economics, 2015. **18**(2): p. 63-73.

137. Howard, L., et al., *Effectiveness and cost-effectiveness of admissions to women's crisis houses compared with traditional psychiatric wards: pilot patient-preference randomised controlled trial.* Br J Psychiatry Suppl, 2010. **53**: p. s32-40.

138. Ising, H., et al., *Cost-effectiveness of preventing first-episode psychosis in ultra-high-risk subjects: Multi-centre randomized controlled trial.* Psychological Medicine, 2015. **45**(7): p. 1435-1446.

139. Ising, H.K., et al., *Four-Year Cost-effectiveness of Cognitive Behavior Therapy for Preventing First-episode Psychosis: The Dutch Early Detection Intervention Evaluation (EDIE-NL) Trial.* Schizophr Bull, 2017. **43**(2): p. 365-374.

140. Jiménez Rodríguez, T.W. and A. Martí Gil, *Analysis of cost minimizing impact on the budget of the national health system for attention deficit hyperactivity disorder treatment with immediate release in place of different extended release methylphenidate alternatives.* Acta Pediatrica Espanola, 2016. **74**(9): p. 209-216.

141. Johnston, M.C., A. Ludbrook, and M.A. Jaffray, *Inequalities in the distribution of the costs of alcohol misuse in Scotland: a cost of illness study.* Alcohol Alcohol, 2012. **47**(6): p. 725-31.

142. Joling, K.J., et al., *The cost-effectiveness of a family meetings intervention to prevent depression and anxiety in family caregivers of patients with dementia: a randomized trial.* Trials, 2013. **14**: p. 305.

143. Joling, K.J., et al., *Predictors of societal costs in dementia patients and their informal caregivers: A two-year prospective cohort study.* The American Journal of Geriatric Psychiatry, 2015. **23**(11): p. 1193-1203.

144. Jones, R.W., et al., *Dependence in Alzheimer's disease and service use costs, quality of life, and caregiver burden: the DADE study.* Alzheimers Dement, 2015. **11**(3): p. 280-90.

145. Jonkers, C.C.M., et al., *Economic evaluation of a minimal psychological intervention in chronically ill elderly patients with minor or mild to moderate depression: A randomized trial (the DELTA-study).* International Journal of Technology Assessment in Health Care, 2009. **25**(4): p. 497-504.

146. Kallert, T.W. and I. Nitsche, *[Direct health-related costs of severely mentally ill patients and their informal carers in community care].* Neuropsychiatr, 2010. **24**(1): p. 42-55.

147. Kane, E., et al., *A cost and economic evaluation of the Leeds personality disorder managed clinical network-A service and commissioning development initiative.* Personal Ment Health, 2016. **10**(3): p. 169-80.

148. Karow, A., Reimer, J, Konig, HH, Heider, D, Bock, T, Huber, C, Schottle, D, Meister, K, Rietschel, L, Ohm, G, Schulz, H, Naber, D,, *Cost-effectiveness of 12-month assertive community treatment as part of integrated care versus standard care in patients with schizophrenia.* Journal of Clinical Psychiatry, 2012. **73**(3): p. e402-e408.

149. Kendrick, T., et al., *Randomised controlled trial to determine the clinical effectiveness and cost-effectiveness of selective serotonin reuptake inhibitors plus supportive care, versus supportive care alone, for mild to moderate depression with somatic symptoms in primary care: The THREAD (THREshold for AntiDepressant response) study.* Health Technology Assessment, 2009. **13**(22): p. 1-159.

150. Kessler, R.C., et al., *The prevalence and workplace costs of adult attention deficit hyperactivity disorder in a large manufacturing firm.* Psychological Medicine, 2009. **39**(1): p. 137-147.

151. Kiencke, P., et al., *[Cost of illness in Alzheimer's disease].* Med Klin (Munich), 2010. **105**(5): p. 327-33.

152. Killaspy, H., et al., *Quality of life, autonomy, satisfaction, and costs associated with mental health supported accommodation services in England: a national survey.* Lancet Psychiatry, 2016. **3**(12): p. 1129-1137.

153. Kleine-Budde, K., et al., *The cost of depression-A cost analysis from a large database.* Journal of Affective Disorders, 2013. **147**(1-3): p. 137-143.

154. Klora, M., et al., *Costs and treatment patterns of incident ADHD patients - a comparative analysis before and after the initial diagnosis.* Health Economics Review, 2015. **5**(1): p. 1-9.

155. Klora, M., et al., *Age- and Gender-specific Costs as Well as Drug Therapies of ADHD Patients.* Gesundheitswesen (Bundesverband der Arzte des Offentlichen Gesundheitsdienstes (Germany)), 2016. **78**(7): p. e23-e29.

156. Knapp, M., et al., *Cost effectiveness of a manual based coping strategy programme in promoting the mental health of family carers of people with dementia (the START (STrAtegies for RelaTives) study): a pragmatic randomised controlled trial.* Bmj, 2013. **347**: p. f6342.

157. Knapp, M., R. Romeo, and J. Beecham, *Economic cost of autism in the UK.* Autism, 2009. **13**(3): p. 317-336.

158. Knapp, M., et al., *Cost-utility analysis of treatment with olanzapine compared with other antipsychotic treatments in patients with schizophrenia in the pan-European SOHO study.* PharmacoEconomics, 2008. **26**(4): p. 341-358.

159. Knapp M, R.R., Mogg A, Eranti S, Pluck G, Purvis R, et al, *Cost-effectiveness of transcranial magnetic stimulation vs. electroconvulsive therapy for severe depression: a multi-centre randomized controlled trial. .* J Affect Disord, 2008. **109**(3): p. 273-285.

160. Kohlboeck, G., et al., *Healthcare use and costs associated with children's behavior problems.* European Child & Adolescent Psychiatry, 2014. **23**(8): p. 701-714.

161. Kolovos, S., et al., *Economic evaluation of Internet-based problem-solving guided self-help treatment in comparison with enhanced usual care for depressed outpatients waiting for face-to-face treatment: A randomized controlled trial.* J Affect Disord, 2016. **200**: p. 284-92.

162. König, H.H., et al., *Economic evaluation of cognitive behavioral therapy and Internet-based guided self-help for binge-eating disorder.* International Journal of Eating Disorders, 2018. **51**(2): p. 155-164.

163. Konig, H.H., et al., *Cost-effectiveness of a primary care model for anxiety disorders.* Br J Psychiatry, 2009. **195**(4): p. 308-17.

164. Konig, H.H., T. Grochtdreis, and C. Brettschneider, *[Health Economic Evaluations within the Hamburg Network for Mental Health].* Psychiatr Prax, 2015. **42 Suppl 1**: p. S70-4.

165. Konig, H.H., et al., *[The regional psychiatry budget (RPB): a model for a new payment system of hospital based mental health care services].* Psychiatr Prax, 2010. **37**(1): p. 34-42.

166. Konnopka, A., et al., *Association of costs with somatic symptom severity in patients with medically unexplained symptoms.* Journal of Psychosomatic Research, 2013. **75**(4): p. 370-375.

167. Konnopka, A., et al., *Cost-utility of a specific collaborative group intervention for patients with functional somatic syndromes.* Journal of Psychosomatic Research, 2016. **90**: p. 43-50.

168. Kraan, T.C., et al., *The effect of childhood adversity on 4-year outcome in individuals at ultra high risk for psychosis in the Dutch Early Detection Intervention Evaluation (EDIE-NL) Trial.* Psychiatry Research, 2017. **247**: p. 55-62.

169. Kuyken, W., et al., *The effectiveness and cost-effectiveness of mindfulness-based cognitive therapy compared with maintenance antidepressant treatment in the prevention of depressive relapse/recurrence: results of a randomised controlled trial (the PREVENT study).* Health Technol Assess, 2015. **19**(73): p. 1-124.

170. Laezer, K.L., et al., *Expensive Long-Term Psychotherapy vsCost-Effective Combined Behavioral Therapy/Medication Treatment: Comparison of Total Treatment Costs of Children with ADHD and ODD.* Gesundheitsokonomie und Qualitatsmanagement, 2015. **20**(4): p. 178-185.

171. Lambert, R.A., et al., *Cost-effectiveness analysis of an occupational therapy-led lifestyle approach and routine general practitioner's care for panic disorder.* Social psychiatry and psychiatric epidemiology, 2010. **45**(7): p. 741-750.

172. Lammerts, L., et al., *A participatory supportive return to work program for workers without an employment contract, sick-listed due to a common mental disorder: an economic evaluation alongside a randomized controlled trial.* BMC Public Health, 2017. **17**(1): p. 162.

173. Laurenssen, E.M., et al., *The burden of disease in patients eligible for mentalization-based treatment (MBT): quality of life and costs.* Health Qual Life Outcomes, 2016. **14**(1): p. 145.

174. Leicht, H., et al., *Net costs of dementia by disease stage.* Acta Psychiatr Scand, 2011. **124**(5): p. 384-95.

175. Leicht, H. and H.H. Konig, *[Costs of illness in dementia from a societal perspective. An overview].* Bundesgesundheitsblatt Gesundheitsforschung Gesundheitsschutz, 2012. **55**(5): p. 677-84.

176. Lenox-Smith, A., et al., *Resource utilisation, costs and clinical outcomes in non-institutionalised patients with Alzheimer's disease: 18-month UK results from the GERAS observational study.* BMC Geriatr, 2016. **16**(1): p. 195.

177. Leue, C., et al., *Managing complex patients on a medical psychiatric unit: An observational study of university hospital costs associated with medical service use, length of stay, and psychiatric intervention.* Journal of Psychosomatic Research, 2010. **68**(3): p. 295-302.

178. Lewis, H., et al., *CollAborative care and active surveillance for Screen-Positive EldeRs with subthreshold depression (CASPER): a multicentred randomised controlled trial of clinical effectiveness and cost-effectiveness.* Health Technol Assess, 2017. **21**(8): p. 1-196.

179. Littlewood, E., et al., *A randomised controlled trial of computerised cognitive behaviour therapy for the treatment of depression in primary care: the Randomised Evaluation of the Effectiveness and Acceptability of Computerised Therapy (REEACT) trial.* Health Technol Assess, 2015. **19**(101): p. viii, xxi-171.

180. Livingston, G., et al., *Long-term clinical and cost-effectiveness of psychological intervention for family carers of people with dementia: A single-blind, randomised, controlled trial.* The Lancet Psychiatry, 2014. **1**(7): p. 539-548.

181. Livingston, G., et al., *START (STrAtegies for RelaTives) study: a pragmatic randomised controlled trial to determine the clinical effectiveness and cost-effectiveness of a manual-based coping strategy programme in promoting the mental health of carers of people with dementia.* Health Technol Assess, 2014. **18**(61): p. 1-242.

182. Lokkerbol, J., et al., *[A study of cost-effectiveness of treating serious mental illness: challenges and solutions].* Tijdschr Psychiatr, 2016. **58**(10): p. 700-705.

183. Lokman, S., et al., *Return-to-work intervention versus usual care for sick-listed employees: Health-economic investment appraisal alongside a cluster randomised trial.* BMJ Open, 2017. **7**(10).

184. Lopez-Nunez, C., et al., *Cost-effectiveness of a voucher-based intervention for smoking cessation.* Am J Drug Alcohol Abuse, 2016. **42**(3): p. 296-305.

185. Lovell, K., et al., *Clinical effectiveness, cost-effectiveness and acceptability of low-intensity interventions in the management of obsessive-compulsive disorder: the Obsessive-Compulsive Treatment Efficacy randomised controlled Trial (OCTET).* Health Technol Assess, 2017. **21**(37): p. 1-132.

186. Lücke, C., et al., *A comparison of two psychiatric service approaches: Findings from the Consultation vs. Liaison Psychiatry-Study.* BMC Psychiatry, 2017. **17**(1).

187. Luppa, M., et al., *Direct costs associated with mild cognitive impairment in primary care.* International Journal of Geriatric Psychiatry, 2008. **23**(9): p. 963-971.

188. Luppa, M., et al., *Direct costs associated with depression in old age in Germany.* Journal of Affective Disorders, 2008. **105**(1-3): p. 195-204.

189. Luppa M, H.S., Angermeyer MC, Konig HH, Riedel-Heller SG *Healthcare costs associated with recognized and unrecognized depression in old age. .* International Psychogeriatrics 2008. **20**(6): p. 1219-1229.

190. Macdonald, G., et al., *The effectiveness, acceptability and cost-effectiveness of psychosocial interventions for maltreated children and adolescents: an evidence synthesis.* Health Technol Assess, 2016. **20**(69): p. 1-508.

191. Malins, S., et al., *Cognitive behaviour therapy for long-term frequent attenders in primary care: a feasibility case series and treatment development study.* Br J Gen Pract, 2016. **66**(651): p. e729-36.

192. Manthey, J., et al., *Economic burden associated with alcohol dependence in a German primary care sample: a bottom-up study.* BMC public health, 2016. **16**: p. 906.

193. McCrone, P., et al., *Cost-effectiveness of an early intervention service for people with psychosis.* Br J Psychiatry, 2010. **196**(5): p. 377-82.

194. McCrone, P., et al., *The REACT study: Cost-effectiveness analysis of assertive community treatment in North London.* Psychiatric Services, 2009. **60**(7): p. 908-913.

195. McCrone, P., et al., *Computer-aided self-exposure therapy for phobia/panic disorder: A pilot economic evaluation.* Cognitive Behaviour Therapy, 2009. **38**(2): p. 91-99.

196. McCrone, P., et al., *The economic cost of treatment-resistant depression in patients referred to a specialist service.* Journal of Mental Health, 2017: p. 1-7.

197. McGilloway, S., et al., *Reducing child conduct disordered behaviour and improving parent mental health in disadvantaged families: a 12-month follow-up and cost analysis of a parenting intervention.* European Child and Adolescent Psychiatry, 2014. **23**(9): p. 783-794.

198. McMurran, M., et al., *Psychoeducation with problem-solving (PEPS) therapy for adults with personality disorder: a pragmatic randomised controlled trial to determine the clinical effectiveness and cost-effectiveness of a manualised intervention to improve social functioning.* Health Technol Assess, 2016. **20**(52): p. 1-250.

199. Meeuwsen, E., et al., *Cost-effectiveness of one year dementia follow-up care by memory clinics or general practitioners: economic evaluation of a randomised controlled trial.* PLoS One, 2013. **8**(11): p. e79797.

200. Menn P, H.R., Kunz S, Donath C, Lauterberg J, Leidl R, Marx P, Mehlig H, Ruckdaschel S, Vollmar HC, Wunder S, Grassel E, *Dementia care in the general practice setting: a cluster randomized trial on the effectiveness and cost impact of three management strategies.* Value in Health, 2012. **15**(6): p. 851-859.

201. Mentzakis, E., F. Paolucci, and G. Rubicko, *Priority setting in the Austrian healthcare system: results from a discrete choice experiment and implications for mental health.* J Ment Health Policy Econ, 2014. **17**(2): p. 61-73.

202. Meuldijk, D., et al., *Economic Evaluation of Concise Cognitive Behavioural Therapy and/or Pharmacotherapy for Depressive and Anxiety Disorders.* Journal of Mental Health Policy and Economics, 2015. **18**(4): p. 175-183.

203. Michalowsky, B., et al., *Healthcare resource utilization and cost in dementia: Are there differences between patients screened positive for dementia with and those without a formal diagnosis of dementia in primary care in Germany?* International Psychogeriatrics, 2016. **28**(3): p. 359-369.

204. Michalowsky, B., et al., *Medication cost of persons with dementia in primary care in Germany.* Journal of Alzheimer's Disease, 2014. **42**(3): p. 949-958.

205. Michalowsky, B., et al., *Healthcare Utilization and Costs in Primary Care Patients with Dementia: Baseline Results of the DelpHi-Trial.* European Journal of Health Economics, 2018. **19**(1): p. 87-102.

206. Michalowsky, B., et al., *Economic analysis of formal care, informal care, and productivity losses in primary care patients who screened positive for dementia in Germany.* Journal of Alzheimer's Disease, 2016. **50**(1): p. 47-59.

207. Moreno, K., E. Sanchez, and L. Salvador-Carulla, *Methodological advances in unit cost calculation of psychiatric residential care in Spain.* Journal of Mental Health Policy and Economics, 2008. **11**(2): p. 79-88.

208. Morrell, C.J., et al., *Psychological interventions for postnatal depression: cluster randomised trial and economic evaluation. The PoNDER trial.* Health Technol Assess, 2009. **13**(30): p. iii-iv, xi-xiii, 1-153.

209. Morriss, R., et al., *Efficacy and cost-effectiveness of a specialist depression service versus usual specialist mental health care to manage persistent depression: a randomised controlled trial.* The Lancet Psychiatry, 2016. **3**(9): p. 821-831.

210. Mostardt, S., et al., *[Efficacy and cost effectiveness of case management in patients with dementia].* Z Gerontol Geriatr, 2012. **45**(7): p. 642-6.

211. Mukuria, C., et al., *Cost-effectiveness of an improving access to psychological therapies service.* British Journal of Psychiatry, 2013. **202**(3): p. 220-227.

212. Murphy, D.G.M., et al., *Autism in adults. New biologicial findings and their translational implications to the cost of clinical services.* Brain Research, 2011. **1380**: p. 22-33.

213. Nijhof, N., et al., *An evaluation of preventive sensor technology for dementia care.* J Telemed Telecare, 2013. **19**(2): p. 95-100.

214. Nikolić, N., et al., *The impact of paliperidone palmitate long-acting injection on hospital admissions in a mental health setting.* International Clinical Psychopharmacology, 2017. **32**(2): p. 95-102.

215. Nollett, C.L., et al., *Depression in Visual Impairment Trial (DEPVIT): A Randomized Clinical Trial of Depression Treatments in People With Low Vision.* Invest Ophthalmol Vis Sci, 2016. **57**(10): p. 4247-54.

216. Oliva-Moreno, J., et al., *The socioeconomic costs of mental illness in Spain.* The European Journal of Health Economics, 2009. **10**(4): p. 361-369.

217. Olivares, J.M., et al., *Cost-effectiveness analysis of switching antipsychotic medication to long-acting injectable risperidone in patients with schizophrenia: A 12- and 24-month follow-up from the e-STAR database in Spain.* Applied Health Economics and Health Policy, 2008. **6**(1): p. 41-53.

218. Ooms, P., et al., *Cost-effectiveness of deep brain stimulation versus treatment as usual for obsessive-compulsive disorder.* Brain Stimul, 2017. **10**(4): p. 836-842.

219. Orgeta, V., et al., *Individual cognitive stimulation therapy for dementia: a clinical effectiveness and cost-effectiveness pragmatic, multicentre, randomised controlled trial.* Health Technol Assess, 2015. **19**(64): p. 1-108.

220. Ormston, R., et al., *quit4u: the effectiveness of combining behavioural support, pharmacotherapy and financial incentives to support smoking cessation.* Health Educ Res, 2015. **30**(1): p. 121-33.

221. Pamias Massana, M., et al., *The social cost of depression in the city of Sabadell (Barcelona, Spain) (2007-2008).* Gaceta Sanitaria, 2012. **26**(2): p. 153-158.

222. Parker, S.G., et al., *Rehabilitation of older patients: day hospital compared with rehabilitation at home. A randomised controlled trial.* Health technology assessment (Winchester, England), 2009. **13**(39): p. 1-143, iii-iv.

223. Patel, A., et al., *Cognitive remediation therapy in schizophrenia: cost-effectiveness analysis.* Schizophr Res, 2010. **120**(1-3): p. 217-24.

224. Patel A, M.P., Leese M, Amaddeo F, Tansella M, Kilian R, et al. , *Cost-effectiveness of adherence therapy versus health education for people with schizophrenia: randomised controlled trial in four European countries. .* Cost Eff Resour Alloc, 2013. **11**(1): p. 12.

225. Paulus, A.T., A.J. van Raak, and H.J. Maarse, *Is integrated nursing home care cheaper than traditional care? A cost comparison.* Int J Nurs Stud, 2008. **45**(12): p. 1764-77.

226. Peckham, E., et al., *Smoking cessation intervention for severe mental ill health trial (SCIMITAR): A pilot randomised control trial of the clinical effectiveness and cost-effectiveness of a bespoke smoking cessation service.* Health Technology Assessment, 2015. **19**(25): p. 1-148.

227. Perry, J., et al., *Adults with intellectual disabilities and challenging behaviour: The costs and outcomes of in- and out-of-area placements.* Journal of Intellectual Disability Research, 2013. **57**(2): p. 139-152.

228. Peters, E., et al., *A randomised controlled trial of cognitive behaviour therapy for psychosis in a routine clinical service.* Acta Psychiatrica Scandinavica, 2010. **122**(4): p. 302-318.

229. Petrou, S., et al., *Economic costs and preference-based health-related quality of life outcomes associated with childhood psychiatric disorders.* Br J Psychiatry, 2010. **197**(5): p. 395-404.

230. Petrou, S., et al., *The association between neurodevelopmental disability and economic outcomes during mid-childhood.* Child: care, health and development, 2013. **39**(3): p. 345-357.

231. Priebe, K., et al., *Costs of Mental Health Care in Patients with Posttraumatic Stress Disorder Related to Sexual Abuse One Year before and after Inpatient DBT-PTSD.* Psychiatrische Praxis, 2017. **44**(2): p. 75-84.

232. Priebe, S., et al., *Effectiveness and cost-effectiveness of dialectical behaviour therapy for self-harming patients with personality disorder: A pragmatic randomised controlled trial.* Psychotherapy and Psychosomatics, 2012. **81**(6): p. 356-365.

233. Priebe, S., et al., *Financial incentives to improve adherence to antipsychotic maintenance medication in non-adherent patients: A cluster randomised controlled trial.* Health Technology Assessment, 2016. **20**(70): p. v-121.

234. Priebe, S., et al., *Treatment outcomes and costs at specialized centers for the treatment of PTSD after the war in former Yugoslavia.* Psychiatric Services, 2010. **61**(6): p. 598-604.

235. Priebe, S., et al., *The Effectiveness of a Patient-Centred Assessment with a Solution-Focused Approach (DIALOG+) for Patients with Psychosis: A Pragmatic Cluster-Randomised Controlled Trial in Community Care.* Psychotherapy and Psychosomatics, 2015. **84**(5): p. 304-313.

236. Priebe, S., et al., *Consequences of untreated posttraumatic stress disorder following war in former yugoslavia: Morbidity, subjective quality of life, and care costs.* Croatian Medical Journal, 2009. **50**(5): p. 465-475.

237. Priebe, S., et al., *Clinical effectiveness and cost-effectiveness of body psychotherapy in the treatment of negative symptoms of schizophrenia: A multicentre randomised controlled trial.* Health Technology Assessment, 2016. **20**(11): p. 1-100.

238. Prins, M., et al., *The costs of guideline-concordant care and of care according to patients' needs in anxiety and depression.* Journal of Evaluation in Clinical Practice, 2011. **17**(4): p. 537-546.

239. Puschner, B., et al., *Cost-effectiveness of needs-oriented discharge planning in high utilizers of mental health care.* Psychiatrische Praxis, 2012. **39**(8): p. 381-387.

240. Quintero, J., et al., *Health care and societal costs of the management of children and adolescents with attention-deficit/hyperactivity disorder in Spain: A descriptive analysis.* BMC Psychiatry, 2018. **18**(1).

241. Ranger, M., et al., *Cost-effectiveness of nidotherapy for comorbid personality disorder and severe mental illness: randomized controlled trial.* Epidemiol Psichiatr Soc, 2009. **18**(2): p. 128-36.

242. Rebergen, D.S., et al., *Cost-effectiveness of guideline-based care for workers with mental health problems.* Journal of Occupational and Environmental Medicine, 2009. **51**(3): p. 313-322.

243. Reed, C., et al., *Identifying factors of activities of daily living important for cost and caregiver outcomes in Alzheimer's disease.* Int Psychogeriatr, 2016. **28**(2): p. 247-59.

244. Reed, C., et al., *What drives country differences in cost of Alzheimer's disease? An explanation from resource use in the GERAS study.* Journal of Alzheimer's Disease, 2017. **57**(3): p. 797-812.

245. Reese, J.P., et al., *Cost and care of patients with Alzheimer's disease: Clinical predictors in German health care settings.* Journal of Alzheimer's Disease, 2011. **27**(4): p. 723-736.

246. Rehberg, W., U. Fürstenau, and B. Rhiner, *Multisystemic Therapy (MST) for youths with severe conduct disorders - Economic evaluation of the implementation in a German-speaking environment.* Zeitschrift fur Kinder- und Jugendpsychiatrie und Psychotherapie, 2011. **39**(1): p. 41-45.

247. Richards, D.A., et al., *Clinical effectiveness and cost-effectiveness of collaborative care for depression in UK primary care (CADET): A cluster randomised controlled trial.* Health Technology Assessment, 2016. **20**(14): p. 1-192.

248. Richards, D.A., et al., *Cost and Outcome of Behavioural Activation versus Cognitive Behavioural Therapy for Depression (COBRA): a randomised, controlled, non-inferiority trial.* The Lancet, 2016. **388**(10047): p. 871-880.

249. Richards, D.A., et al., *Cost and outcome of behavioural activation (COBRA): A randomised controlled trial of behavioural activation versus cognitive-behavioural therapy for depression.* Health Technology Assessment, 2017. **21**(46): p. i-365.

250. Ricken, R., et al., *Algorithm-guided treatment of depression reduces treatment costs - results from the randomized controlled German Algorithm Project (GAPII).* Journal of Affective Disorders, 2011. **134**(1-3): p. 249-256.

251. Ricken, R., et al., *A standardized stepwise drug treatment algorithm for depression reduces direct treatment costs in depressed inpatients-Results from the German Algorithm Project (GAP3).* Journal of Affective Disorders, 2018. **228**: p. 173-177.

252. Rive, B., et al., *Predicting time to full-time care in AD: A new model.* Journal of Medical Economics, 2010. **13**(2): p. 362-370.

253. Rivera, B., B. Casal, and L. Currais, *The social cost of illicit drugs use in Spain.* Int J Drug Policy, 2017. **44**: p. 92-104.

254. Roick, C., et al., *[The regional psychiatry budget: costs and effects of a new multisector financing model for psychiatric care].* Psychiatr Prax, 2008. **35**(6): p. 279-85.

255. Romeo, R., et al., *Cost-effectiveness analyses for mirtazapine and sertraline in dementia: Randomised controlled trial.* British Journal of Psychiatry, 2013. **202**(2): p. 121-128.

256. Romeo, R., et al., *Cost estimation of a health-check intervention for adults with intellectual disabilities in the UK.* Journal of Intellectual Disability Research, 2009. **53**(5): p. 426-439.

257. Romeo, R., et al., *The treatment of challenging behaviour in intellectual disabilities: Cost-effectiveness analysis.* Journal of Intellectual Disability Research, 2009. **53**(7): p. 633-643.

258. Romero-Sanchiz, P., et al., *Economic evaluation of a guided and unguided internet-based CBT intervention for major depression: Results from a multi-center, three-armed randomized controlled trial conducted in primary care.* PLoS One, 2017. **12**(2): p. e0172741.

259. Romeyke, T., H.C. Scheuer, and H. Stummer, *Inpatient hospital costs and length of stay for the treatment of affective and somatoform disorders – Evidence from Germany.* Risk Management and Healthcare Policy, 2014. **7**: p. 245-252.

260. Rovira, J., et al., *The cost of generalized anxiety disorder in primary care settings: results of the ANCORA study.* Community mental health journal, 2012. **48**(3): p. 372-383.

261. Rubio-Valera, M., et al., *Cost-effectiveness of a community pharmacist intervention in patients with depression: a randomized controlled trial (PRODEFAR Study).* PLoS One, 2013. **8**(8): p. e70588.

262. Sagoo, G.S., et al., *Cost Effectiveness of Using Array-CGH for Diagnosing Learning Disability.* Applied Health Economics and Health Policy, 2015. **13**(4): p. 421-432.

263. Salize, H.J., et al., *[Optimized quality of care for affective disorders by health insurance-based case-management: a controlled cost-study].* Psychiatr Prax, 2014. **41**(8): p. 432-8.

264. Salize, H.J., et al., *Treating alcoholism reduces financial burden on care-givers and increases quality-adjusted life years.* Addiction (Abingdon, England), 2013. **108**(1): p. 62-70.

265. Salize, H.J., et al., *Cost of treatment of schizophrenia in six European countries.* Schizophrenia Research, 2009. **111**(1-3): p. 70-77.

266. Salize, H.J., et al., *Cost-effective primary care-based strategies to improve smoking cessation: more value for money.* Arch Intern Med, 2009. **169**(3): p. 230-5; discussion 235-6.

267. Salvador-Carulla, L., et al., *Costs of depression in Catalonia (Spain).* Journal of Affective Disorders, 2011. **132**(1-2): p. 130-138.

268. Salvador-Carulla, L., et al., *Cost of borderline personality disorder in Catalonia (Spain).* Eur Psychiatry, 2014. **29**(8): p. 490-7.

269. Sayal, K., et al., *Effectiveness and cost-effectiveness of a brief school-based group programme for parents of children at risk of ADHD: a cluster randomised controlled trial.* Child Care Health Dev, 2016. **42**(4): p. 521-33.

270. Schmidt-Kraepelin, C., B. Janssen, and W. Gaebel, *Prevention of rehospitalization in schizophrenia: Results of an integrated care project in Germany.* European Archives of Psychiatry and Clinical Neuroscience, 2009. **259**(SUPPL. 2): p. S205-S212.

271. Schöffski, O., S. Sohn, and M. Happich, *Overall burden to society caused by hyperkinetic syndrome (HKS) and attention deficit hyperactivity disorder (ADHD).* Gesundheitswesen, 2008. **70**(7): p. 398-403.

272. Schwarzkopf, L., P. Menn, and R. Holle, *[Discretionary decisions in claims data analyses and their effects - an explorative comparison using the example of a cost of illness study on dementia].* Gesundheitswesen, 2012. **74**(8-9): p. e76-83.

273. Schwarzkopf, L., et al., *Costs of care for dementia patients in community setting: an analysis for mild and moderate disease stage.* Value Health, 2011. **14**(6): p. 827-35.

274. Schwarzkopf, L., et al., *Excess costs of dementia disorders and the role of age and gender - an analysis of German health and long-term care insurance claims data.* BMC Health Serv Res, 2012. **12**: p. 165.

275. Scott, J., et al., *Long-term mental health resource utilization and cost of care following group psychoeducation or unstructured group support for bipolar disorders: A cost-benefit analysis.* Journal of Clinical Psychiatry, 2009. **70**(3): p. 378-386.

276. Seivewright, H., et al., *Cognitive-behavioural therapy for health anxiety in a genitourinary medicine clinic: Randomised controlled trial.* British Journal of Psychiatry, 2008. **193**(4): p. 332-337.

277. Serrano-Blanco, A., et al., *In-patient costs of agitation and containment in a mental health catchment area.* BMC Psychiatry, 2017. **17**(1).

278. Serrano-Blanco, A., et al., *Fluoxetine and imipramine: Are there differences in cost-utility for depression in primary care?* Journal of Evaluation in Clinical Practice, 2009. **15**(1): p. 195-203.

279. Sevilla-Dedieu, C., *Mental Health Care and Out-of-Pocket Expenditures in Europe: Results from the ESEMeD Project.* Journal of Mental Health Policy and Economics, 2011. **14**(2): p. 95-105.

280. Sicras-Mainar, A., et al., *[Impact of morbidity, resource use and costs on maintenance of remission of major depression in Spain: a longitudinal study in a population setting].* Gac Sanit, 2010. **24**(1): p. 13-9.

281. Sicras-Mainar, A., et al., *Influence of morbidity and the use of health resources in patients who require care for generalised anxiety disorder in the primary health care setting.* Atencion Primaria, 2008. **40**(12): p. 603-610.

282. Sicras-Mainar, A., J. Ejas-Gutiérrez, and R. Navarro-Artieda, *Comparative effectiveness and costs of generic and brand-name gabapentin and venlafaxine in patients with neuropathic pain or generalized anxiety disorder in Spain.* ClinicoEconomics and Outcomes Research, 2015. **7**: p. 299-312.

283. Sicras-Mainar, A., et al., *Influence of the CYP2D6 isoenzyme in patients treated with venlafaxine for major depressive disorder: Clinical and economic consequences.* PLoS ONE, 2014. **9**(11).

284. Sicras-Mainar, A., et al., *Costs and associated factors with optimal and suboptimal responses to the treatment of major depressive disorder.* Atencion Primaria, 2012. **44**(11): p. 667-675.

285. Sicras-Mainar, A., et al., *Assessment of pharmacological strategies for management of major depressive disorder and their costs after an inadequate response to first-line antidepressant treatment in primary care.* Annals of General Psychiatry, 2012. **11**.

286. Sicras-Mainar, A., et al., *Impact of negative symptoms on healthcare resource utilization and associated costs in adult outpatients with schizophrenia: A population-based study.* BMC Psychiatry, 2014. **14**(1).

287. Sicras-Mainar, A., et al., *Comparison of escitalopram vs. citalopram and venlafaxine in the treatment of major depression in Spain: clinical and economic consequences.* Curr Med Res Opin, 2010. **26**(12): p. 2757-64.

288. Simon, E., et al., *Cost-effectiveness of child-focused and parent-focused interventions in a child anxiety prevention program.* J Anxiety Disord, 2012. **26**(2): p. 287-96.

289. Simon, J., et al., *Remote mood monitoring for adults with bipolar disorder: An explorative study of compliance and impact on mental health service use and costs.* European Psychiatry, 2017. **45**: p. 14-19.

290. Simons, C.J.P., et al., *Economic evaluation of an experience sampling method intervention in depression compared with treatment as usual using data from a randomized controlled trial.* BMC Psychiatry, 2017. **17**(1).

291. Slade, M., et al., *Alternatives to standard acute in-patient care in England: short-term clinical outcomes and cost-effectiveness.* Br J Psychiatry Suppl, 2010. **53**: p. s14-9.

292. Smit, E.S., et al., *Cost-effectiveness and cost-utility of Internet-based computer tailoring for smoking cessation.* J Med Internet Res, 2013. **15**(3): p. e57.

293. Smit, F., et al., *Preventing panic disorder: Cost-effectiveness analysis alongside a pragmatic randomised trial.* Cost Effectiveness and Resource Allocation, 2009. **7**.

294. Snell, T., et al., *Economic impact of childhood psychiatric disorder on public sector services in Britain: estimates from national survey data.* J Child Psychol Psychiatry, 2013. **54**(9): p. 977-85.

295. Soeteman, D.I., et al., *Cost-effective psychotherapy for personality disorders in the Netherlands: the value of further research and active implementation.* Value Health, 2011. **14**(2): p. 229-39.

296. Soeteman, D.I., et al., *The economic burden of personality disorders in mental health care.* Journal of Clinical Psychiatry, 2008. **69**(2): p. 259-265.

297. Sonuga-Barke, E.J.S., et al., *A comparison of the clinical effectiveness and cost of specialised individually delivered parent training for preschool attention-deficit/hyperactivity disorder and a generic, group-based programme: a multi-centre, randomised controlled trial of the New Forest Parenting Programme versus Incredible Years.* European Child and Adolescent Psychiatry, 2017: p. 1-13.

298. Soto-Gordoa, M., et al., *[The cost of applying the Dependency Law to Alzheimer disease].* Gac Sanit, 2014. **28**(5): p. 389-92.

299. Spackman, E., et al., *Cost-effectiveness analysis of acupuncture, counselling and usual care in treating patients with depression: the results of the ACUDep trial.* PLoS One, 2014. **9**(11): p. e113726.

300. Speetjens, P., et al., *Child maltreatment: Long-term economic consequences and implications.* Tijdschrift voor Psychiatrie, 2016. **58**(10): p. 706-711.

301. Spill, B., et al., *Long-term observation of patients successfully switched to risperidone long-acting injectable: A retrospective, naturalistic 18-month mirror-image study of hospitalization rates and therapy costs.* International Journal of Psychiatry in Clinical Practice, 2010. **14**(1): p. 53-62.

302. Stamm, K., I. Reinhard, and H.J. Salize, *Long-term health insurance payments for depression in Germany - A secondary analysis of routine data.* Neuropsychiatrie, 2010. **24**(2): p. 99-107.

303. Stant, A., et al., *Cost-effectiveness of a psychoeducational relapse prevention program for depression in primary care.* Journal of Mental Health Policy and Economics, 2009. **12**(4): p. 195-204.

304. Stant, A.D., et al., *Economic aspects of peer support groups for psychosis.* Community Ment Health J, 2011. **47**(1): p. 99-105.

305. Stant, A.D., et al., *Cost-effectiveness of cognitive self-therapy in patients with depression and anxiety disorders.* Acta Psychiatrica Scandinavica, 2008. **117**(1): p. 57-66.

306. Stargardt, T., et al., *Effectiveness and cost of atypical versus typical antipsychotic treatment in a nationwide cohort of patients with schizophrenia in Germany.* Journal of Clinical Psychopharmacology, 2012. **32**(5): p. 602-607.

307. Stargardt, T., et al., *Effectiveness and costs of flupentixol compared to other first- and second-generation antipsychotics in the treatment of schizophrenia.* Psychopharmacology (Berl), 2011. **216**(4): p. 579-87.

308. Stargardt, T., et al., *Effectiveness and cost of atypical versus typical antipsychotic treatment for schizophrenia in routine care.* J Ment Health Policy Econ, 2008. **11**(2): p. 89-97.

309. Steinert, T., C. Bischof, and F. Eisele, *Clinical consequences and cost effectiveness of brain imaging in psychiatric clinical care.* Gesundheitsokonomie und Qualitatsmanagement, 2009. **14**(4): p. 178-183.

310. Stevenson, M.D., A. Scope, and P.A. Sutcliffe, *The cost-effectiveness of group cognitive behavioral therapy compared with routine primary care for women with postnatal depression in the UK.* Value Health, 2010. **13**(5): p. 580-4.

311. Strydom, A., et al., *Service use and cost of mental disorder in older adults with intellectual disability.* British Journal of Psychiatry, 2010. **196**(2): p. 133-138.

312. Stuhldreher, N., et al., *The costs of social anxiety disorder: the role of symptom severity and comorbidities.* J Affect Disord, 2014. **165**: p. 87-94.

313. Stuhldreher, N., et al., *Determinants of direct and indirect costs in anorexia nervosa.* International Journal of Eating Disorders, 2015. **48**(1): p. 139-146.

314. Tafalla, M., et al., *Pattern of healthcare resource utilization and direct costs associated with manic episodes in Spain.* BMC Psychiatry, 2010. **10**.

315. Tanajewski, L., et al., *Economic Evaluation of a General Hospital Unit for Older People with Delirium and Dementia (TEAM Randomised Controlled Trial).* PLoS One, 2015. **10**(12): p. e0140662.

316. Telford, C., et al., *Estimating the costs of ongoing care for adolescents with attention-deficit hyperactivity disorder.* Social psychiatry and psychiatric epidemiology, 2013. **48**(2): p. 337-344.

317. Tempest, M., et al., *Cost-effectiveness Analysis of Aripiprazole Once-Monthly for the Treatment of Schizophrenia in the UK.* J Ment Health Policy Econ, 2015. **18**(4): p. 185-200.

318. Timman, R., et al., *Development of the Treatment Inventory of Costs in Psychiatric Patients: TIC-P Mini and Midi.* Value in Health, 2015. **18**(8): p. 994-999.

319. Toghanian, S., et al., *Economic and humanistic burden of illness in generalized anxiety disorder: An analysis of patient survey data in Europe.* ClinicoEconomics and Outcomes Research, 2014. **6**(1): p. 151-163.

320. Tsiachristas, A., et al., *Economic impact of early intervention in psychosis services: results from a longitudinal retrospective controlled study in England.* BMJ Open, 2016. **6**(10): p. e012611.

321. Turner-Stokes, L., A. Bill, and R. Dredge, *A cost analysis of specialist inpatient neurorehabilitation services in the UK.* Clin Rehabil, 2012. **26**(3): p. 256-63.

322. Turner-Stokes, L., et al., *Using the UKROC dataset to make the case for resources to improve cost-efficiency in neurological rehabilitation.* Disabil Rehabil, 2012. **34**(22): p. 1900-6.

323. Turner-Stokes, L., S. Sutch, and R. Dredge, *Healthcare tariffs for specialist inpatient neurorehabilitation services: Rationale and development of a UK casemix and costing methodology.* Clinical Rehabilitation, 2012. **26**(3): p. 264-279.

324. Turro -Garriga O, L.-P.S., Vilalta-Franch J, Turon-Estrada A, Pericot- Nierga I, et al. , *Annual economic cost of informal care in Alzheimer’s disease.* Rev Neurol, 2010. **51**: p. 201-207.

325. Twomey, C., A. Cieza, and D.S. Baldwin, *Utility of functioning in predicting costs of care for patients with mood and anxiety disorders: A prospective cohort study.* International Clinical Psychopharmacology, 2017. **32**(4): p. 205-212.

326. Tyrer, P., et al., *Clinical and cost-effectiveness of cognitive behaviour therapy for health anxiety in medical patients: a multicentre randomised controlled trial.* Lancet, 2014. **383**(9913): p. 219-25.

327. Tyrer, P., et al., *Nidotherapy in the treatment of substance misuse, psychosis and personality disorder: Secondary analysis of a controlled trial.* Psychiatrist, 2011. **35**(1): p. 9-14.

328. Tyrer, P., et al., *Neuroleptics in the treatment of aggressive challenging behaviour for people with intellectual disabilities: A randomised controlled trial (NACHBID).* Health Technology Assessment, 2009. **13**(21): p. 1-54.

329. Tyrer, P., et al., *Cognitive-behaviour therapy for health anxiety in medical patients (Champ): A randomised controlled trial with outcomes to 5 years.* Health Technology Assessment, 2017. **21**(50): p. 1-88.

330. Uegaki, K., et al., *Cost-effectiveness of a minimal intervention for stress-related sick leave in general practice: results of an economic evaluation alongside a pragmatic randomised control trial.* J Affect Disord, 2010. **120**(1-3): p. 177-87.

331. Underwood M, L.S., Eldridge S, Sheehan B, Slowther A, Spencer A, et al. , *Exercise for depression in care home residents: a randomised controlled trial with cost-effectiveness analysis (OPERA). .* Health Technol Assess. , 2013. **17**(1): p. 1-281.

332. Valladares, A., T. Dilla, and J. Sacristan, *Depression: A social mortgage: Latest advances in knowledge of the cost of the diasease.* Actas Espanolas de Psiquiatria, 2009. **37**(1): p. 49-53.

333. van 't Veer-Tazelaar, P.J., P. Cuijpers, and A.J. Beekman, *[Prevention of depression and anxiety in older people].* Tijdschr Psychiatr, 2011. **53**(9): p. 579-84.

334. van Apeldoorn, F.J., et al., *Cost-effectiveness of CBT, SSRI, and CBT+SSRI in the treatment for panic disorder.* Acta Psychiatr Scand, 2014. **129**(4): p. 286-95.

335. van Asselt, A.D., et al., *Out-patient psychotherapy for borderline personality disorder: cost-effectiveness of schema-focused therapy v. transference-focused psychotherapy.* Br J Psychiatry, 2008. **192**(6): p. 450-7.

336. van Asselt, A.D., et al., *Difficulties in calculating productivity costs: work disability associated with borderline personality disorder.* Value Health, 2008. **11**(4): p. 637-44.

337. van der Aa, H.P.A., et al., *Economic evaluation of stepped-care versus usual care for depression and anxiety in older adults with vision impairment: randomized controlled trial.* BMC Psychiatry, 2017. **17**(1): p. 280.

338. Van Der Gaag, M., et al., *Cognitive-behavioural therapy for persistent and recurrent psychosis in people with schizophrenia-spectrum disorder: Cost-effectiveness analysis.* British Journal of Psychiatry, 2011. **198**(1): p. 59-65.

339. van der Weele GM, d.W.M., van den Hout WB, de Craen AJ, Spinhoven P, Stijnen T, Assendelft WJ, van der Mast RC, Gussekloo J, *Effects of a stepped-care intervention programme among older subjects who screened positive for depressive symptoms in general practice.* Age and Ageing, 2012. **41**(4): p. 482-488.

340. van Mierlo, L.D., et al., *[Implementation and (cost-)effectiveness of case management for people with dementia and their informal caregivers: results of the COMPAS study].* Tijdschr Gerontol Geriatr, 2016. **47**(6): p. 223-233.

341. van Oostrom, S.H., et al., *Economic evaluation of a workplace intervention for sick-listed employees with distress.* Occup Environ Med, 2010. **67**(9): p. 603-10.

342. van Ravesteijn, H., et al., *Mindfulness-based cognitive therapy for patients with medically unexplained symptoms: a cost-effectiveness study.* J Psychosom Res, 2013. **74**(3): p. 197-205.

343. van Spijker, B.A., et al., *Reducing suicidal ideation: cost-effectiveness analysis of a randomized controlled trial of unguided web-based self-help.* J Med Internet Res, 2012. **14**(5): p. e141.

344. van Steensel, F.J., C.D. Dirksen, and S.M. Bogels, *A cost of illness study of children with high-functioning autism spectrum disorders and comorbid anxiety disorders as compared to clinically anxious and typically developing children.* J Autism Dev Disord, 2013. **43**(12): p. 2878-90.

345. Van Steensel, F.J.A., C.D. Dirksen, and S.M. Bögels, *Cost-effectiveness of cognitive-behavioral therapy versus treatment as usual for anxiety disorders in children with autism spectrum disorder.* Research in Autism Spectrum Disorders, 2014. **8**(2): p. 127-137.

346. Veer-Tazelaar, P.V., et al., *Cost-effectiveness of a stepped care intervention to prevent depression and anxiety in late life: Randomised trial.* British Journal of Psychiatry, 2010. **196**(4): p. 319-325.

347. Vemer, P., et al., *If you try to stop smoking, should we pay for it? The cost-utility of reimbursing smoking cessation support in the Netherlands.* Addiction, 2010. **105**(6): p. 1088-97.

348. Vermeulen, K.M., et al., *Cost-effectiveness of multisystemic therapy versus usual treatment for young people with antisocial problems.* Crim Behav Ment Health, 2017. **27**(1): p. 89-102.

349. Vila, A., et al., *Cost-effectiveness of a Barcelona home care program for individuals with multimorbidity.* J Am Geriatr Soc, 2015. **63**(5): p. 1017-24.

350. Vos de Wael, N., *[Cooperation and client perspective are the terms for success; reflection on the cost-effectiveness of psychiatry].* Tijdschr Psychiatr, 2016. **58**(10): p. 766-770.

351. Vroomen, J.M., et al., *The cost-effectiveness of two forms of case management compared to a control group for persons with dementia and their informal caregivers from a societal perspective.* PLoS ONE Vol 11(9), 2016, ArtID e0160908, 2016. **11**(9).

352. Wade, A.G., et al., *Escitalopram and duloxetine in major depressive disorder: A pharmacoeconomic comparison using UK cost data.* PharmacoEconomics, 2008. **26**(11): p. 969-981.

353. Wade, A.G., et al., *Healthcare expenditure in severely depressed patients treated with escitalopram, generic SSRIs or venlafaxine in the UK.* Current Medical Research and Opinion, 2010. **26**(5): p. 1161-1170.

354. Wagner, C.J., et al., *Depression-related treatment and costs in Germany: Do they change with comorbidity? A claims data analysis.* Journal of Affective Disorders, 2016. **193**: p. 257-266.

355. Wagner, T., et al., *Societal cost-of-illness in patients with borderline personality disorder one year before, during and after dialectical behavior therapy in routine outpatient care.* Behaviour Research and Therapy, 2014. **61**: p. 12-22.

356. Wagner, T., et al., *Societal cost-of-illness of borderline personality disorder.* Zeitschrift fur Klinische Psychologie und Psychotherapie: Forschung und Praxis, 2013. **42**(4): p. 242-255.

357. Wansink, H.J., et al., *Cost-effectiveness of preventive case management for parents with a mental illness: a randomized controlled trial from three economic perspectives.* BMC Health Serv Res, 2016. **16**: p. 228.

358. Warmerdam, L., et al., *Cost-utility and cost-effectiveness of internet-based treatment for adults with depressive symptoms: randomized trial.* J Med Internet Res, 2010. **12**(5): p. e53.

359. Watson, J.M., et al., *AESOPS: a randomised controlled trial of the clinical effectiveness and cost-effectiveness of opportunistic screening and stepped care interventions for older hazardous alcohol users in primary care.* Health Technol Assess, 2013. **17**(25): p. 1-158.

360. Watson, J.M., et al., *ADAPTA: A pilot randomised controlled trial of an alcohol-focused intervention versus a healthy living intervention for problem drinkers identified in a general hospital setting.* Drug and Alcohol Dependence, 2015. **154**: p. 117-124.

361. Wehmeier, P.M., A. Schacht, and A. Rothenberger, *Change in the direct cost of treatment for children and adolescents with hyperkinetic disorder in Germany over a period of four years.* Child and Adolescent Psychiatry and Mental Health, 2009. **3**.

362. Weiss, F.D., W. Rief, and M. Kleinstäuber, *Health care utilization in outpatients with somatoform disorders: Descriptives, interdiagnostic differences, and potential mediating factors.* General Hospital Psychiatry, 2017. **44**: p. 22-29.

363. Weschenfelder, A.K., et al., *Economic evaluation of a manual-based, multimodal cognitive behavioural therapy for school avoiding children with psychiatric disorder.* Zeitschrift fur Kinder- und Jugendpsychiatrie und Psychotherapie, 2018. **46**(1): p. 47-56.

364. Wetzelaer, P., et al., *Cost-effectiveness and Budget Impact of Specialized Psychotherapy for Borderline Personality Disorder: A Synthesis of the Evidence.* J Ment Health Policy Econ, 2017. **20**(4): p. 177-190.

365. Wild, B., et al., *Significance and costs of complex biopsychosocial health care needs in elderly people: results of a population-based study.* Psychosom Med, 2014. **76**(7): p. 497-502.

366. Wiles, N., et al., *Clinical effectiveness and cost-effectiveness of cognitive behavioural therapy as an adjunct to pharmacotherapy for treatment-resistant depression in primary care: The CoBalT randomised controlled trial.* Health Technology Assessment, 2014. **18**(31): p. 1-167.

367. Wiley-Exley, E., et al., *Cost-effectiveness of integrated care for elderly depressed patients in the PRISM-E study.* Journal of Mental Health Policy and Economics, 2009. **12**(4): p. 205-213+217+220.

368. Wilson E, T.M., Shepstone L, Charlesworth G, Poland F, Harvey I, et al. , *Befriending carers of people with dementia: a cost utility analysis.* Int J Geriatr Psychiatry. , 2009. **24**(6): p. 610-623.

369. Wimo, A., et al., *The GERAS Study: a prospective observational study of costs and resource use in community dwellers with Alzheimer's disease in three European countries--study design and baseline findings.* J Alzheimers Dis, 2013. **36**(2): p. 385-99.

370. Wolfs, C.A., et al., *Economic evaluation of an integrated diagnostic approach for psychogeriatric patients: Results of a randomized controlled trial.* Archives of General Psychiatry, 2009. **66**(3): p. 313-323.

371. Wolfs, C.A., et al., *[The costs and benefits of an integrated approach to dementia].* Tijdschr Psychiatr, 2011. **53**(9): p. 657-65.

372. Woods, R.T., et al., *REMCARE: Reminiscence groups for people with dementia and their family caregivers - Effectiveness and costeffectiveness pragmatic multicentre randomised trial.* Health Technology Assessment, 2012. **16**(48): p. v-vii.

373. Wu, Q., et al., *Economic cost of smoking in people with mental disorders in the UK.* Tob Control, 2015. **24**(5): p. 462-8.

374. Wubker, A., et al., *Costs of Care for People with Dementia Just before and after Nursing Home Placement: Primary Data from Eight European Countries.* European Journal of Health Economics, 2015. **16**(7): p. 689-707.

375. Wunsch, E.M., S. Kliem, and C. Kroger, *Population-based cost-offset estimation for the treatment of borderline personality disorder: projected costs in a currently running, ideal health system.* Behav Res Ther, 2014. **60**: p. 1-7.

376. Young, A.H., et al., *Annual cost of managing bipolar disorder to the UK healthcare system.* Journal of Affective Disorders, 2011. **133**(3): p. 450-456.

377. Zeidler, J., et al., *Cost effectiveness of paliperidone palmitate for the treatment of schizophrenia in Germany.* Appl Health Econ Health Policy, 2013. **11**(5): p. 509-21.

378. Zentner, N., et al., *Health service costs in people with severe mental illness: Patient report vs. administrative records.* Psychiatrische Praxis, 2012. **39**(3): p. 122-128.

379. Zwijsen, S., et al., *The cost-effectiveness of grip on challenging behaviour: An economic evaluation of a care programme for managing challenging behaviour.* International Journal of Geriatric Psychiatry, 2016. **31**(6): p. 567-574.

## Appendix 3: Excluded publications from systematic literature review

1. Abdul Pari, A.A., et al., *Economic evaluations in bipolar disorder: A systematic review and critical appraisal.* Bipolar Disorders, 2014. **16**(6): p. 557-582.

2. Abu-Akel, A., et al., *Autism tendencies and psychosis proneness interactively modulate saliency cost.* Schizophrenia Bulletin, 2017. **43**(1): p. 142-151.

3. Akmaz, B., et al., *Cost-effectiveness analysis of antidepressants by the Iqwig in Germany.* Value in Health, 2011. **14**(7): p. A301.

4. Anastasiadou, D., et al., *Cost-effectiveness of the mobile application TCApp combined with face-to-face CBT treatment compared to face-to-face CBT treatment alone for patients with an eating disorder: Study protocol of a multi-centre randomised controlled trial.* BMC Psychiatry, 2018. **18**(1).

5. Andersson, E., et al., *Cost-effectiveness of internet-based cognitive behavior therapy for obsessive-compulsive disorder: Results from a randomized controlled trial.* Journal of Obsessive-Compulsive and Related Disorders, 2015. **4**: p. 47-53.

6. Andersson, E., et al., *Cost-effectiveness of an internet-based booster program for patients with obsessive-compulsive disorder: Results from a randomized controlled trial.* Journal of Obsessive-Compulsive and Related Disorders, 2015. **4**: p. 14-19.

7. Aranda-Reneo, I., et al., *Informal care of patients with schizophrenia.* J Ment Health Policy Econ, 2013. **16**(3): p. 99-108.

8. Arends, I., J.J. van der Klink, and U. Bültmann, *Prevention of recurrent sickness absence among employees with common mental disorders: design of a cluster-randomised controlled trial with cost-benefit and effectiveness evaluation.* BMC public health, 2010. **10**: p. 132.

9. Aumann, I., et al., *[Cost-Effectiveness of Pharmacological Smoking Cessation Therapies - A Systematic Literature Review].* Gesundheitswesen, 2016. **78**(10): p. 660-671.

10. Ballesteros, F. and F.J. Labrador, *Empirically supported treatments for panic disorder with agoraphobia in a Spanish psychology clinic.* The Spanish journal of psychology, 2014. **17**: p. E65.

11. Bamelis, L.L., S.M. Evers, and A. Arntz, *Design of a multicentered randomized controlled trial on the clinical and cost effectiveness of schema therapy for personality disorders.* BMC Public Health, 2012. **12**: p. 75.

12. Barnett, P.G., et al., *Cost-effectiveness of integrating tobacco cessation into post-traumatic stress disorder treatment.* Nicotine and Tobacco Research, 2016. **18**(3): p. 267-274.

13. Barr, W., et al., *Quantitative findings from a mixed methods evaluation of once-weekly therapeutic community day services for people with personality disorder.* Journal of Mental Health, 2010. **19**(5): p. 412-421.

14. Barrett, B. and S. Byford, *Costs and outcomes of an intervention programme for offenders with personality disorders.* British Journal of Psychiatry, 2012. **200**(4): p. 336-341.

15. Barrett, B. and P. Tyrer, *The cost-effectiveness of the dangerous and severe personality disorder programme.* Criminal Behaviour and Mental Health, 2012. **22**(3): p. 202-209.

16. Barrett, B., et al., *An examination of the factors that influence costs in medical patients with health anxiety.* Journal of Psychosomatic Research, 2012. **73**(1): p. 59-62.

17. Bee, P., et al., *The clinical effectiveness, cost-effectiveness and acceptability of community-based interventions aimed at improving or maintaining quality of life in children of parents with serious mental illness: a systematic review.* Health Technol Assess, 2014. **18**(8): p. 1-250.

18. Bergström, J., et al., *Internet-versus group-administered cognitive behaviour therapy for panic disorder in a psychiatric setting: A randomised trial.* BMC Psychiatry, 2010. **10**.

19. Bermingham, S.L., et al., *The cost of somatisation among the working-age population in England for the year 2008-2009.* Mental Health in Family Medicine, 2010. **7**(2): p. 71-84.

20. Bitran, S. and S.G. Hofmann, *Effect of affect on social cost bias in social anxiety disorder.* Anxiety, Stress and Coping, 2010. **23**(3): p. 273-287.

21. Blom, M.M., et al., *Effectiveness and cost-effectiveness of an internet intervention for family caregivers of people with dementia: design of a randomized controlled trial.* BMC Psychiatry, 2013. **13**: p. 17.

22. Bock, J.-O., et al., *Cost-effectiveness of the treatment of depression in old age: A systematic review.* Psychiatrische Praxis, 2015. **42**(5): p. 240-247.

23. Bockting, C.L., et al., *Disrupting the rhythm of depression using Mobile Cognitive Therapy for recurrent depression: randomized controlled trial design and protocol.* BMC Psychiatry, 2011. **11**: p. 12.

24. Bockting, C.L.H., et al., *Disrupting the rhythm of depression: Design and protocol of a randomized controlled trial on preventing relapse using brief cognitive therapy with or without antidepressants.* BMC Psychiatry, 2011. **11**.

25. Boege, I., et al., *Hometreatment-An effective alternative to inpatient treatment in child and adolescent psychiatry?* Zeitschrift fur Kinder- und Jugendpsychiatrie und Psychotherapie, 2015. **43**(6): p. 411-423.

26. Bolinski, F., et al., *Effectiveness of a transdiagnostic individually tailored Internet-based and mobile-supported intervention for the indicated prevention of depression and anxiety (ICare Prevent) in Dutch college students: Study protocol for a randomised controlled trial.* Trials, 2018. **19**(1).

27. Bonin, E.M., et al., *Costs and longer-term savings of parenting programmes for the prevention of persistent conduct disorder: a modelling study.* BMC public health, 2011. **11**: p. 803.

28. Boss, L., et al., *Evaluating the (cost-)effectiveness of guided and unguided Internet-based self-help for problematic alcohol use in employees--a three arm randomized controlled trial.* BMC Public Health, 2015. **15**: p. 1043.

29. Boudrez, H., et al., *Effectiveness of varenicline as an aid to smoking cessation: Results of an inter-European observational study.* Current Medical Research and Opinion, 2011. **27**(4): p. 769-775.

30. Bowers, L. and C. Flood, *Nurse staffing, bed numbers and the cost of acute psychiatric inpatient care in England.* J Psychiatr Ment Health Nurs, 2008. **15**(8): p. 630-7.

31. Boyd, K.A. and A.H. Briggs, *Cost-effectiveness of pharmacy and group behavioural support smoking cessation services in Glasgow.* Addiction, 2009. **104**(2): p. 317-25.

32. Boyer, N.R., et al., *Examining the feasibility of an economic analysis of dyadic developmental psychotherapy for children with maltreatment associated psychiatric problems in the United Kingdom.* BMC Psychiatry, 2014. **14**: p. 346.

33. Bramesfeld, A., et al., *Effectiveness and efficiency of assertive outreach for schizophrenia in Germany: Study protocol on a pragmatic quasi-experimental controlled trial.* BMC Psychiatry Vol 13 2013, ArtID 56, 2013. **13**.

34. Bramham, J., et al., *Evaluation of group cognitive behavioral therapy for adults with ADHD.* Journal of Attention Disorders, 2009. **12**(5): p. 434-441.

35. Braun, S., et al., *Cost of attention deficit/hyperactivity disorder in Germany.* Value in Health, 2010. **13**(7): p. A448.

36. Brettschneider, C., et al., *Cost-utility analyses of cognitive-behavioural therapy of depression: A systematic review.* Psychotherapy and Psychosomatics, 2015. **84**(1): p. 6-21.

37. Brettschneider, C., S. Riedel-Heller, and H.H. König, *A systematic review of cost-of-illness studies and cost-effectiveness analyses in borderline personality disorder.* Value in Health, 2013. **16**(7): p. A546.

38. Brettschneider, C., S. Riedel-Heller, and H.H. König, *A systematic review of economic evaluations of treatments for borderline personality disorder.* PLoS ONE, 2014. **9**(9).

39. Brewin, N., et al., *Evaluation of a Motivation and Psycho-Educational Guided Self-Help Intervention for People with Eating Disorders (MOPED).* European Eating Disorders Review, 2016. **24**(3): p. 241-246.

40. Brierley, G., et al., *Psychological advocacy toward healing (PATH): study protocol for a randomized controlled trial.* Trials, 2013. **14**: p. 221.

41. Brown, J.S., et al., *Outcome, costs and patient engagement for group and individual CBT for depression: a naturalistic clinical study.* Behav Cogn Psychother, 2011. **39**(3): p. 355-8.

42. Bruijniks, S.J., et al., *Frequency and change mechanisms of psychotherapy among depressed patients: study protocol for a multicenter randomized trial comparing twice-weekly versus once-weekly sessions of CBT and IPT.* BMC Psychiatry, 2015. **15**: p. 137.

43. Buescher, A.V.S., et al., *Costs of autism spectrum disorders in the United Kingdom and the United States.* JAMA Pediatrics, 2014. **168**(8): p. 721-728.

44. Bulik, C.M., et al., *CBT4BN versus CBTF2F: Comparison of online versus face-to-face treatment for bulimia nervosa.* Contemporary Clinical Trials, 2012. **33**(5): p. 1056-1064.

45. Buntrock, C., et al., *Evaluating the efficacy and cost-effectiveness of web-based indicated prevention of major depression: Design of a randomised controlled trial.* BMC Psychiatry, 2014. **14**(1).

46. Burns, T., et al., *A randomised controlled trial of time-limited individual placement and support: IPS-LITE trial.* British Journal of Psychiatry, 2015. **207**(4): p. 351-356.

47. Byford, S. and H. Petkova, *Cost-effectiveness of Models of Care for Young People with Eating Disorders (CostED).* Journal of Mental Health Policy and Economics, 2015. **18**: p. S6.

48. Cabasés, J.M., et al., *The quality of life and the health-related costs of patients with generalized anxiety disorder: A Bayesian approach.* Pharmacoeconomics - Spanish Research Articles, 2011. **8**(1): p. 3-15.

49. Camacho, E.M., et al., *Cost-effectiveness of structured group psychoeducation versus unstructured group support for bipolar disorder: Results from a multi-centre pragmatic randomised controlled trial.* Journal of Affective Disorders, 2017. **211**: p. 27-36.

50. Carnero-Pardo, C., et al., *Diagnostic accuracy, effectiveness and cost for cognitive impairment and dementia screening of three short cognitive tests applicable to illiterates.* PLoS ONE, 2011. **6**(11).

51. Castelein, S., et al., *The effectiveness of peer support groups in psychosis: a randomized controlled trial.* Acta Psychiatr Scand, 2008. **118**(1): p. 64-72.

52. Catala-Lopez, F., et al., *[Economic evaluation of neurological and mental disorders in Spain: systematic review and comparative analysis].* Rev Neurol, 2011. **52**(2): p. 65-71.

53. Catala-Lopez, F., et al., *The increasing burden of mental and neurological disorders.* Eur Neuropsychopharmacol, 2013. **23**(11): p. 1337-9.

54. Cedillo, S. and J. Rejas, *Budgetary impact analysis of reimbursement varenicline in the smoking cessation treatment of patients with major depression in Spain.* Value in Health, 2016. **19**(7): p. A522.

55. Cedillo, S. and J. Rejas, *Effect of smoking status on costs-of-illness in patients with a depressive disorder in Spain: An approximation from the national health survey.* Value in Health, 2016. **19**(7): p. A523.

56. Cedillo, S., et al., *Budgetary Impact Analysis of Reimbursement Varenicline for the Smoking-Cessation Treatment in Patients with Cardiovascular Diseases, Chronic Obstructive Pulmonary Disease or Type-2 Diabetes Mellitus: A National Health System Perspective.* Eur Addict Res, 2017. **23**(1): p. 7-18.

57. Cheema, N., S. Frangou, and P. McCrone, *Cost-effectiveness of ethyleicosapentaenoic acid in the treatment of bipolar disorder.* Therapeutic Advances in Psychopharmacology, 2013. **3**(2): p. 73-81.

58. Clignet, F., et al., *The systematic activation method as a nursing intervention in depressed elderly: A protocol for a multi - centre cluster randomized trial.* BMC Psychiatry, 2012. **12**(1).

59. Coleman, T., et al., *Protocol for the Proactive Or Reactive Telephone Smoking CeSsation Support (PORTSSS) trial.* Trials, 2009. **10**: p. 26.

60. Comas-Herrera, A., et al., *MODEM: A comprehensive approach to modelling outcome and costs impacts of interventions for dementia. Protocol paper.* BMC Health Serv Res, 2017. **17**(1): p. 25.

61. Cornelis, J., et al., *Intensive home treatment for patients in acute psychiatric crisis situations: A multicentre randomized controlled trial.* BMC Psychiatry Vol 18 2018, ArtID 55, 2018. **18**.

62. Cottrell, S., et al., *A modeled economic evaluation comparing atomoxetine with stimulant therapy in the treatment of children with attention-deficit/hyperactivity disorder in the United Kingdom.* Value in Health, 2008. **11**(3): p. 376-388.

63. Crawford, M.J., et al., *Lamotrigine versus inert placebo in the treatment of borderline personality disorder: study protocol for a randomized controlled trial and economic evaluation.* Trials, 2015. **16**: p. 308.

64. Creswell, C., et al., *Clinical outcomes and cost-effectiveness of brief guided parent-delivered cognitive behavioural therapy and solution-focused brief therapy for treatment of childhood anxiety disorders: a randomised controlled trial.* The Lancet Psychiatry, 2017. **4**(7): p. 529-539.

65. da Silva Lima, A.F.B., et al., *Cost-effectiveness of treatment for bipolar disorders*, in *Mental health economics: The costs and benefits of psychiatric care*. 2017, Springer International Publishing; Switzerland: Cham, Switzerland. p. 299-311.

66. Davies, A., et al., *Cost-effectiveness of atypical antipsychotics for the management of schizophrenia in the UK.* Curr Med Res Opin, 2008. **24**(11): p. 3275-85.

67. de Graaf, L.E., et al., *Clinical and cost-effectiveness of computerised cognitive behavioural therapy for depression in primary care: design of a randomised trial.* BMC Public Health, 2008. **8**: p. 224.

68. de Jong, M., et al., *Effectiveness of enhanced cognitive behavioral therapy (CBT-E) for eating disorders: Study protocol for a randomized controlled trial.* Trials, 2016. **17**(1).

69. Dear, B.F., et al., *Clinical and Cost-Effectiveness of Therapist-Guided Internet-Delivered Cognitive Behavior Therapy for Older Adults With Symptoms of Anxiety: A Randomized Controlled Trial.* Behavior Therapy, 2015. **46**(2): p. 206-217.

70. Diefenbach, G.J. and D.F. Tolin, *The cost of illness associated with stepped care for obsessive-compulsive disorder.* Journal of Obsessive-Compulsive and Related Disorders, 2013. **2**(2): p. 144-148.

71. Dilla T, M.J., O Donohoe P, Alvarez M, Sacristan JA, Happich M, Tockhorn A, *Long-acting olanzapine versus long-acting risperidone for schizophrenia in Spain: a cost-effectiveness comparison?* BMC Psychiatry, 2014. **14**(1): p. 298.

72. Dixon, J., et al., *Exploring the cost-effectiveness of a one-off screen for dementia (for people aged 75years in England and Wales).* International Journal of Geriatric Psychiatry, 2015. **30**(5): p. 446-452.

73. Dixon, L., *Introduction to the supplement: Directions for future patient-centered and comparative effectiveness research for people with serious mental illness in a learning mental health care system.* Schizophrenia Bulletin, 2014. **40**: p. v-S94.

74. Dobbie, F., et al., *Evaluating Long-term Outcomes of NHS Stop Smoking Services (ELONS): a prospective cohort study.* Health Technol Assess, 2015. **19**(95): p. 1-156.

75. Donath, C., et al., *Day care for dementia patients from a family caregiver's point of view: a questionnaire study on expected quality and predictors of utilisation - Part II.* BMC Health Serv Res, 2011. **11**: p. 76.

76. Donker, T., et al., *Implementation of internet-based preventive interventions for depression and anxiety: Role of support? The design of a randomized controlled trial.* Trials, 2009. **10**.

77. Dossenbach, M., et al., *Long-term antipsychotic monotherapy for schizophrenia: disease burden and comparative outcomes for patients treated with olanzapine, quetiapine, risperidone, or haloperidol monotherapy in a pan-continental observational study.* J Clin Psychiatry, 2008. **69**(12): p. 1901-15.

78. Drost, R.M., et al., *Inter-sectoral costs and benefits of mental health prevention: towards a new classification scheme.* J Ment Health Policy Econ, 2013. **16**(4): p. 179-86.

79. Drost, R.M., et al., *Valuing inter-sectoral costs and benefits of interventions in the healthcare sector: methods for obtaining unit prices.* Expert Rev Pharmacoecon Outcomes Res, 2017. **17**(1): p. 77-84.

80. Drukker, M., et al., *The use of a Cumulative Needs for Care Monitor for individual treatment v. care as usual for patients diagnosed with severe mental illness, a cost-effectiveness analysis from the health care perspective.* Epidemiol Psychiatr Sci, 2012. **21**(4): p. 381-92.

81. Ebenfeld, L., et al., *Efficacy of a hybrid online training for panic symptoms and agoraphobia: Study protocol for a randomized controlled trial.* Trials, 2014. **15**(1).

82. Ebert, D.D., et al., *Efficacy and cost-effectiveness of minimal guided and unguided internet-based mobile supported stress-management in employees with occupational stress: a three-armed randomised controlled trial.* BMC Public Health, 2014. **14**: p. 807.

83. Edginton, E., et al., *TIGA-CUB - manualised psychoanalytic child psychotherapy versus treatment as usual for children aged 5-11 years with treatment-resistant conduct disorders and their primary carers: Study protocol for a randomised controlled feasibility trial.* Trials, 2017. **18**(1).

84. Effertz, T. and K. Mann, *The burden and cost of disorders of the brain in Europe with the inclusion of harmful alcohol use and nicotine addiction.* European Neuropsychopharmacology, 2013. **23**(7): p. 742-748.

85. Eisendrath, S.J., et al., *Mindfulness-based cognitive therapy (MBCT) versus the health-enhancement program (HEP) for adults with treatment-resistant depression: A randomized control trial study protocol.* BMC Complementary and Alternative Medicine, 2014. **14**(1).

86. Ekman, M., et al., *Cost effectiveness of quetiapine in patients with acute bipolar depression and in maintenance treatment after an acute depressive episode.* PharmacoEconomics, 2012. **30**(6): p. 513-530.

87. Elison, S., G. Davies, and J. Ward, *An outcomes evaluation of computerized treatment for problem drinking using breaking free online.* Alcoholism Treatment Quarterly, 2015. **33**(2): p. 185-196.

88. Engel, S., A. Reiter-Jaschke, and B. Hofner, *["EduKation demenz(R)". Psychoeducative training program for relatives of people with dementia].* Z Gerontol Geriatr, 2016. **49**(3): p. 187-95.

89. Escobar, R., et al., *[Attention deficit/hyperactivity disorder: burden of the disease according to subtypes in recently diagnosed children].* Actas Esp Psiquiatr, 2008. **36**(5): p. 285-94.

90. Evans-Lacko, S., et al., *Evaluating the economic impact of screening and treatment for depression in the workplace.* Eur Neuropsychopharmacol, 2016. **26**(6): p. 1004-13.

91. Eveleigh, R., et al., *Cost-utility analysis of a treatment advice to discontinue inappropriate long-term antidepressant use in primary care.* Family Practice, 2014. **31**(5): p. 578-584.

92. Faber A, v.A.M., Kalverdijk L J, Tobi H, de Jong-van den Berg L T, Annemans L, Postma M J, *Long-acting methylphenidate-OROS in youths with attention-deficit hyperactivity disorder suboptimally controlled with immediate-release.* CNS Drugs, 2008. **22**(2): p. 157-170.

93. Fajutrao L, P.B., Liu S, Locklear J, *Cost-effectiveness of quetiapine plus mood stabilizers compared with mood stabilizers alone in the maintenance therapy of bipolar.* Clinical Therapeutics, 2009. **31**(1): p. 1456-1468.

94. Faseru, B., et al., *Changing the default for tobacco-cessation treatment in an inpatient setting: Study protocol of a randomized controlled trial.* Trials, 2017. **18**(1).

95. Feenstra, D.J., P. Luyten, and D.L. Bales, *Mentalization-based treatment for borderline personality disorder in adults and adolescents: For whom, when, and how?* Bulletin of the Menninger Clinic, 2017. **81**(3): p. 264-280.

96. Fernandez de Bobadilla Osorio, J., et al., *[Cost effectiveness analysis of varenicline (Champix) for the treatment of smoking in Spain].* An Med Interna, 2008. **25**(7): p. 342-8.

97. Ferrari, G.R., et al., *Investigating the (cost-) effectiveness of attention bias modification (ABM) for outpatients with major depressive disorder (MDD): a randomized controlled trial protocol.* BMC Psychiatry, 2016. **16**(1): p. 370.

98. Fineberg, N.A., et al., *The size, burden and cost of disorders of the brain in the UK.* J Psychopharmacol, 2013. **27**(9): p. 761-70.

99. Flynn, D., et al., *Dialectical behaviour therapy for treating adults and adolescents with emotional and behavioural dysregulation: Study protocol of a coordinated implementation in a publicly funded health service.* BMC Psychiatry, 2018. **18**(1).

100. Fonagy, P., et al., *Multisystemic therapy versus management as usual in the treatment of adolescent antisocial behaviour (START): a pragmatic, randomised controlled, superiority trial.* The Lancet Psychiatry, 2018. **5**(2): p. 119-133.

101. Frey, S., et al., *Cost-effectiveness of long-acting injectable risperidone versus flupentixol decanoate in the treatment of schizophrenia: A Markov model parameterized using administrative data.* The European Journal of Health Economics, 2014. **15**(2): p. 133-142.

102. Furze, G., et al., *"Prehabilitation" prior to CABG surgery improves physical functioning and depression.* International Journal of Cardiology, 2009. **132**(1): p. 51-58.

103. Garrido, G., et al., *Computer-assisted cognitive remediation therapy in schizophrenia: Durability of the effects and cost-utility analysis.* Psychiatry Research, 2017. **254**: p. 198-204.

104. Garrido Viñado, E., et al., *The economic cost of psychiatric mechanical restraint in Spain.* Psiquiatria Biologica, 2015. **22**(1): p. 12-16.

105. Gartlehner, G., et al., *Comparative benefits and harms of second-generation antidepressants for treating major depressive disorder: An updated meta-analysis.* Annals of Internal Medicine, 2011. **155**(11): p. 772-785.

106. Geraedts, A.S., et al., *The longitudinal prediction of costs due to health care uptake and productivity losses in a cohort of employees with and without depression or anxiety.* Journal of Occupational and Environmental Medicine, 2014. **56**(8): p. 794-801.

107. Gerritsen, D.L., et al., *[Quality of life in dementia, opinions among people with dementia, their professional caregivers, and in literature].* Tijdschr Gerontol Geriatr, 2010. **41**(6): p. 241-55.

108. Getsios, D., et al., *Cost effectiveness of donepezil in the treatment of mild to moderate Alzheimer's disease: A UK evaluation using discrete-event simulation.* PharmacoEconomics, 2010. **28**(5): p. 411-427.

109. Getsios, D., et al., *An economic evaluation of early assessment for Alzheimer's disease in the United Kingdom.* Alzheimer's & Dementia: The Journal of the Alzheimer's Association, 2012. **8**(1): p. 22-30.

110. Giron, M., et al., *Efficacy and effectiveness of individual family intervention on social and clinical functioning and family burden in severe schizophrenia: a 2-year randomized controlled study.* Psychol Med, 2010. **40**(1): p. 73-84.

111. Glasper, A., *Tackling the burden of dementia care in society.* Br J Nurs, 2014. **23**(13): p. 752-3.

112. Godman, B., et al., *Potential to enhance the prescribing of generic drugs in patients with mental health problems in Austria; implications for the future.* Frontiers in Pharmacology, 2013. **4 JAN**.

113. Goorden, M., et al., *The cost-effectiveness of family/family-based therapy for treatment of externalizing disorders, substance use disorders and delinquency: a systematic review.* BMC Psychiatry, 2016. **16**: p. 237.

114. Gorska, S., et al., *Service-related needs of older people with dementia: Perspectives of service users and their unpaid carers.* International Psychogeriatrics, 2013. **25**(7): p. 1107-1114.

115. Gospodarevskaya, E. and L. Segal, *Cost-utility analysis of different treatments for post-traumatic stress disorder in sexually abused children.* Child and Adolescent Psychiatry and Mental Health, 2012. **6**.

116. Grandes, G., et al., *The burden of mental disorders in primary care.* Eur Psychiatry, 2011. **26**(7): p. 428-35.

117. Griffin, S.C., et al., *Methodological issues in undertaking independent cost-effectiveness analysis for NICE: The case of therapies for ADHD.* European Journal of Health Economics, 2008. **9**(2): p. 137-145.

118. Grochtdreis, T., et al., *Cost-effectiveness of collaborative care for the treatment of depressive disorders in primary care: a systematic review.* PLoS One, 2015. **10**(5): p. e0123078.

119. Grupp, H., H.-H. Konig, and A. Konnopka, *Cost Measurement of Mental Disorders in Germany.* Journal of Mental Health Policy and Economics, 2014. **17**(1): p. 3-8.

120. Grupp, H., H.H. König, and A. Konnopka, *Health care utilisation and costs in the general population in Germany.* Health Policy, 2016. **120**(2): p. 159-169.

121. Guerriero, C., et al., *The cost-effectiveness of smoking cessation support delivered by mobile phone text messaging: Txt2stop.* Eur J Health Econ, 2013. **14**(5): p. 789-97.

122. Gusi, N., et al., *Cost-utility of a walking programme for moderately depressed, obese, or overweight elderly women in primary care: A randomised controlled trial.* BMC Public Health, 2008. **8**.

123. Gutiérrez-Maldonado, J., et al., *Virtual reality to train diagnostic skills in eating disorders. Comparison of two low cost systems.* Annual Review of CyberTherapy and Telemedicine, 2015. **13**: p. 75-81.

124. Hacker, J., T. Hackmann, and S. Moog, *Demenzkranke und Pflegebedurftige in der Sozialen Pflegeversicherung--Ein intertemporaler Kostenvergleich. (With English summary.).* Schmollers Jahrbuch: Zeitschrift fur Wirtschafts- und Sozialwissenschaften/Journal of Applied Social Science Studies, 2009. **129**(3): p. 445-471.

125. Hagens, P., et al., *Effectiveness of intensive smoking reduction counselling plus combination nicotine replacement therapy in promoting long-term abstinence in patients with chronic obstructive pulmonary disease not ready to quit smoking: Protocol of the REDUQ trial.* Contemporary Clinical Trials Communications, 2017. **8**: p. 248-257.

126. Hamann, J., et al., *Over-the-counter-drugs for the treatment of mood and anxiety disorders - The views of German pharmacists.* Pharmacopsychiatry, 2014. **47**(3): p. 84-88.

127. Hammond, G.C., et al., *Comparative effectiveness of cognitive therapies delivered face-to-face or over the telephone: an observational study using propensity methods.* PLoS One, 2012. **7**(9): p. e42916.

128. Hannover, W., N. Spate, and H.J. Hannich, *[The outpatient provision of care for mental disorders in a rural area. An analysis of reimbursement claims in Mecklenburg-West Pomerania].* Psychother Psychosom Med Psychol, 2011. **61**(8): p. 372-6.

129. Harries, C., *The real cost of eating disorders.* Community Practitioner, 2012. **85**(10): p. 14-16.

130. Harrison, P.J., et al., *Innovative approaches to bipolar disorder and its treatment*. 2016. p. 76-89.

131. Harter, M., et al., *Evaluating a collaborative smoking cessation intervention in primary care (ENTER): study protocol for a cluster-randomized controlled trial.* Trials, 2015. **16**: p. 447.

132. Harter, M., et al., *[Collaborative and stepped care for depression: Development of a model project within the Hamburg Network for Mental Health (psychenet.de)].* Bundesgesundheitsblatt Gesundheitsforschung Gesundheitsschutz, 2015. **58**(4-5): p. 420-9.

133. Hartz S, G.D., Tao S, Blume S, Maclaine G, *Evaluating the cost effectiveness of donepezil in the treatment of Alzheimer's disease in Germany using discrete event simulation.* BMC Neurology 2012. **12**(2).

134. Harwood, R.H., et al., *Evaluation of a Medical and Mental Health Unit compared with standard care for older people whose emergency admission to an acute general hospital is complicated by concurrent 'confusion': a controlled clinical trial. Acronym: TEAM: Trial of an Elderly Acute care Medical and mental health unit.* Trials, 2011. **12**: p. 123.

135. Hassiotis, A., et al., *Applied behaviour analysis and standard treatment in intellectual disability: 2-Year outcomes.* British Journal of Psychiatry, 2011. **198**(6): p. 490-491.

136. Hassiotis, A., et al., *Cognitive behaviour therapy (CBT) for anxiety and depression in adults with mild intellectual disabilities (ID): a pilot randomised controlled trial.* Trials, 2011. **12**: p. 95.

137. Hassiotis, A., M. Serfaty, and R. Sheehan, *Psychological treatments for depression in adults with mild to moderate intellectual disabilities: are we there yet?* The Lancet Psychiatry, 2017. **4**(12): p. 888-889.

138. Hawkes, N., *Talking therapies: Can the centre hold? Can centralised programmes provide better care more cheaply?* BMJ, 2011. **342**(7797): p. 578.

139. Hay, L., *The cost of mental ill health to employers.* Perspect Public Health, 2010. **130**(2): p. 56.

140. Hayden, J., M. Flood, and F. McNicholas, *ADHD in children: a path to free medicines.* Irish Journal of Medical Science, 2016. **185**(1): p. 171-175.

141. Heber, E., et al., *Efficacy and cost-effectiveness of a web-based and mobile stress-management intervention for employees: design of a randomized controlled trial.* BMC public health, 2013. **13**: p. 655.

142. Hedman, E., et al., *Cost-effectiveness and long-term effectiveness of internet-based cognitive behaviour therapy for severe health anxiety.* Psychological medicine, 2013. **43**(2): p. 363-374.

143. Hedman, E., et al., *Cost-effectiveness of Internet-based cognitive behavior therapy vs. cognitive behavioral group therapy for social anxiety disorder: Results from a randomized controlled trial.* Behaviour Research and Therapy, 2011. **49**(11): p. 729-736.

144. Hedman, E., et al., *Clinical effectiveness and cost-effectiveness of Internet- vs. group-based cognitive behavior therapy for social anxiety disorder: 4-Year follow-up of a randomized trial.* Behaviour Research and Therapy, 2014. **59**: p. 20-29.

145. Heeg, B., et al., *The cost-effectiveness of atypicals in the UK.* Value Health, 2008. **11**(7): p. 1007-21.

146. Hees, H.L., et al., *Effectiveness of adjuvant occupational therapy in employees with depression: design of a randomized controlled trial.* BMC Public Health, 2010. **10**: p. 558.

147. Hegerl, U. and L. Wittenburg, *Focus on mental health care reforms in Europe: the European alliance against depression: a multilevel approach to the prevention of suicidal behavior.* Psychiatr Serv, 2009. **60**(5): p. 596-9.

148. Heitmann, J., et al., *Internet-based attentional bias modification training as add-on to regular treatment in alcohol and cannabis dependent outpatients: A study protocol of a randomized control trial.* BMC Psychiatry, 2017. **17**(1).

149. Hennelly, D. and B. Lawlor, *Management of dementia in a general hospital setting.* CME Journal Geriatric Medicine, 2008. **10**(1): p. 14-19.

150. Hernandez-Tejada, M.A., S. Hamski, and D. Sánchez-Carracedo, *Incorporating peer support during in vivo exposure to reverse dropout from prolonged exposure therapy for posttraumatic stress disorder: Clinical outcomes.* International Journal of Psychiatry in Medicine, 2017. **52**(4-6): p. 366-380.

151. Herpertz-Dahlmann, B. and H. Salbach-Andrae, *Overview of Treatment Modalities in Adolescent Anorexia Nervosa.* Child and Adolescent Psychiatric Clinics of North America, 2009. **18**(1): p. 131-145.

152. Herpertz-Dahlmann, B., et al., *Day-patient treatment after short inpatient care versus continued inpatient treatment in adolescents with anorexia nervosa (ANDI): a multicentre, randomised, open-label, non-inferiority trial.* Lancet, 2014. **383**(9924): p. 1222-9.

153. Heslin, M., et al., *Decision analytic model exploring the cost and cost-offset implications of street triage.* BMJ Open, 2016. **6**(2): p. e009670.

154. Heuft, G., G. Hildenbrand, and U. Cuntz, *Current information from the German Society for Psychosomatic Medicine and Medical Psychotherapy: The Hospital Finance Law (KHRG), the Psych-Procedures numbers and the new agreement concerning remuneration of services for psychosomatic medicine and psychotherapy as well as psychiatry and psychotherapy.* Zeitschrift fur Psychosomatische Medizin und Psychotherapie, 2010. **56**(1): p. 86-105.

155. Hilbert, A., *Cognitive-behavioral therapy for binge eating disorder in adolescents: study protocol for a randomized controlled trial.* Trials, 2013. **14**: p. 312.

156. Hirsch, J.K., et al., *Illness burden and symptoms of anxiety in older adults: optimism and pessimism as moderators.* International psychogeriatrics / IPA, 2012. **24**(10): p. 1614-1621.

157. Hodgkins, P., et al., *The pharmacology and clinical outcomes of amphetamines to treat ADHD: does composition matter?* CNS Drugs, 2012. **26**(3): p. 245-68.

158. Hoek, W., et al., *Prevention of depression and anxiety in adolescents: A randomized controlled trial testing the efficacy and mechanisms of Internet-based self-help problem-solving therapy.* Trials, 2009. **10**: p. 93.

159. Hogarth, L. and L. Hardy, *Alcohol use disorder symptoms are associated with greater relative value ascribed to alcohol, but not greater discounting of costs imposed on alcohol.* Psychopharmacology, 2018: p. 1-10.

160. Hohls, J.K., et al., *A systematic review of the association of anxiety with health care utilization and costs in people aged 65 years and older.* Journal of Affective Disorders, 2018. **232**: p. 163-176.

161. Hollander, E., et al., *The cost and impact of compulsivity: A research perspective.* Eur Neuropsychopharmacol, 2016. **26**(5): p. 800-9.

162. Hollingworth, W., et al., *Reducing smoking in adolescents: cost-effectiveness results from the cluster randomized ASSIST (A Stop Smoking In Schools Trial).* Nicotine Tob Res, 2012. **14**(2): p. 161-8.

163. Hong, J., T. Dilla, and J. Arellano, *A modelled economic evaluation comparing atomoxetine with methylphenidate in the treatment of children with attention-deficit/ hyperactivity disorder in Spain.* BMC Psychiatry, 2009. **9**.

164. Hong J, D.T., Arellano J, *A modelled economic evaluation comparing atomoxetine with methylphenidate in the treatment of children with attention-deficit/hyperactivity.* BMC Psychiatry 2009. **9**(15).

165. Hoogveldt B, R.B., Severens J, Maman K, Guilhaume C, *Cost-effectiveness analysis of memantine for moderate-to-severe Alzheimer's disease in the Netherlands.* Neuropsychiatric Disease and Treatment 2011. **7**(1): p. 313-317.

166. Hooper, C., S. Lovestone, and R. Sainz-Fuertes, *Alzheimer's disease, diagnosis and the need for biomarkers.* Biomarker Insights, 2008. **2008**(3): p. 317-323.

167. Houghton, S., D. Saxon, and A. Smallwood, *Effects of opt-in letters in a National Health Service psychotherapy service.* Psychiatrist, 2010. **34**(12): p. 507-510.

168. Howard, L.M., K. Trevillion, and R. Agnew-Davies, *Domestic violence and mental health.* International Review of Psychiatry, 2010. **22**(5): p. 525-534.

169. Howes, O.D., S.J. Lim, and P. Fusar-Poli, *Mind the translation gap: problems in the implementation of early intervention services.* Psychol Med, 2010. **40**(1): p. 171-2.

170. Howlin, P., *Redressing the balance in autism research.* Nature Clinical Practice Neurology, 2008. **4**(8): p. 407.

171. Huijbers, M.J., et al., *Preventing relapse in recurrent depression using mindfulness-based cognitive therapy, antidepressant medication or the combination: trial design and protocol of the MOMENT study.* BMC Psychiatry, 2012. **12**: p. 125.

172. Ibrahim, A.K., et al., *Establishing the reliability and validity of the Zagazig Depression Scale in a UK student population: an online pilot study.* BMC Psychiatry, 2010. **10**: p. 107.

173. Ikkos, G., P. Sugarman, and N. Bouras, *Mental health services commissioning and provision: Lessons from the UK?* Psychiatriki, 2015. **26**(3): p. 181-7.

174. Iliffe, S., et al., *Dementia diagnosis in primary care: Thinking outside the educational box.* Aging Health, 2009. **5**(1): p. 51-59.

175. Ilyas, S. and J. Moncrieff, *Trends in prescriptions and costs of drugs for mental disorders in England, 1998-2010.* British Journal of Psychiatry, 2012. **200**(5): p. 393-398.

176. Indave, B.I., et al., *[Research methods on alcohol-related harm in the population].* Rev Esp Salud Publica, 2014. **88**(4): p. 447-68.

177. Ivano Scandurra, R., A. Garcia-Altes, and M. Nebot, *[Social impact of abusive alcohol consumption in Spain: consumption, cost and policies].* Rev Esp Salud Publica, 2011. **85**(2): p. 141-7.

178. Jacke, C.O. and H.J. Salize, *Cost effectiveness of a health insurance based case management programme for patients with affective disorders.* Neuropsychiatrie, 2014. **28**(3): p. 130-141.

179. Jacob, L. and K. Kostev, *Impact of comorbidities on the cost of depression drug therapy in general practices in Germany.* Journal of Psychiatric Research, 2016. **83**: p. 130-136.

180. Jacobi, F. and S. Kessler-Scheil, *Epidemiology of mental disorders. Frequency and disease burden in Germany.* Psychotherapeut, 2013. **58**(2): p. 191-206.

181. Jacobs, R., et al., *Determinants of hospital length of stay for people with serious mental illness in England and implications for payment systems: a regression analysis.* BMC health services research, 2015. **15**: p. 439.

182. Jahoda, A., et al., *Comparison of behavioural activation with guided self-help for treatment of depression in adults with intellectual disabilities: a randomised controlled trial.* The Lancet Psychiatry, 2017. **4**(12): p. 909-919.

183. Jahoda, A., et al., *BEAT-IT: Comparing a behavioural activation treatment for depression in adults with intellectual disabilities with an attention control: study protocol for a randomised controlled trial.* Trials, 2015. **16**: p. 595.

184. James, A., *Cash in hand.* Ment Health Today, 2008: p. 14-6.

185. Jansen, D.E., et al., *Cost-effectiveness of Multisystemic Therapy for adolescents with antisocial behaviour: study protocol of a randomized controlled trial.* BMC Public Health, 2013. **13**: p. 369.

186. Janssen, L., et al., *Mindfulness based cognitive therapy versus treatment as usual in adults with attention deficit hyperactivity disorder (ADHD).* BMC Psychiatry, 2015. **15**: p. 216.

187. Janssen, N., et al., *Behavioural activation by mental health nurses for late-life depression in primary care: a randomized controlled trial.* BMC Psychiatry, 2017. **17**(1): p. 230.

188. Jascenoka, J., et al., *[Short- and long-term effects of parent training programmes of children with developmental disabilities].* Praxis der Kinderpsychologie und Kinderpsychiatrie, 2013. **62**(5): p. 348-367.

189. Jenkins, P.E., et al., *Comparison of face-to-face versus email guided self-help for binge eating: study protocol for a randomised controlled trial.* Trials, 2014. **15**: p. 181.

190. Jenkinson, J. and R. Howard, *Provision of specialist continuing care services for older adults across the UK.* International Psychogeriatrics, 2016. **28**(6): p. 959-966.

191. Jessen, F., et al., *Prediction of dementia in primary care patients.* PLoS ONE, 2011. **6**(2).

192. Jeurissen, P.P., et al., *Towards a sustainable, cost-effective mental health care; a policy perspective.* Tijdschrift voor Psychiatrie, 2016. **58**(10): p. 683-687.

193. Jick, H., et al., *Comparison of prescription drug costs in the United States and the United Kingdom, part 3: Methylphenidate.* Pharmacotherapy, 2012. **32**(11): p. 970-973.

194. Jin, H. and P. McCrone, *Cost-of-Illness Studies for Bipolar Disorder: Systematic Review of International Studies.* PharmacoEconomics, 2015. **33**(4): p. 341-353.

195. Jing, Y., et al., *Healthcare costs associated with treatment of bipolar disorder using a mood stabilizer plus adjunctive aripiprazole, quetiapine, risperidone, olanzapine or ziprasidone.* Journal of Medical Economics, 2009. **12**(2): p. 104-113.

196. Joling, K.J., et al., *(Cost)-effectiveness of family meetings on indicated prevention of anxiety and depressive symptoms and disorders of primary family caregivers of patients with dementia: Design of a randomized controlled trial.* BMC Geriatrics, 2008. **8**.

197. Jones, M. and D. Mountain, *Patient information sheets in emergency care.* BMJ (Online), 2009. **338**(7696).

198. Jones, S., et al., *A randomised controlled trial of time limited CBT informed psychological therapy for anxiety in bipolar disorder.* BMC Psychiatry, 2013. **13**.

199. Jones, S.H., et al., *Recovery-focused cognitive-behavioural therapy for recent-onset bipolar disorder: Randomized controlled pilot trial.* British Journal of Psychiatry, 2015. **206**(1): p. 58-66.

200. Jonsson, L. and A. Wimo, *The cost of dementia in Europe: A review of the evidence, and methodological considerations.* PharmacoEconomics, 2009. **27**(5): p. 391-403.

201. Judit, H., et al., *Amisulpridre the switching effect of schizophrenia-related costs, one-year follow-up.* Psychiatria Hungarica, 2008. **23**(6): p. 464-471.

202. Justicia, A., et al., *Rationale and methods of the iFightDepression study: A double-blind, randomized controlled trial evaluating the efficacy of an internet-based self-management tool for moderate to mild depression.* BMC Psychiatry, 2017. **17**(1).

203. Kamp-Becker, I., et al., *Study protocol of the ASD-Net, the German research consortium for the study of Autism Spectrum Disorder across the lifespan: From a better etiological understanding, through valid diagnosis, to more effective health care.* BMC Psychiatry, 2017. **17**(1).

204. Karlsdotter, K., et al., *Burden of illness and health care resource utilization in adult psychiatric outpatients with attention-deficit/hyperactivity disorder in Europe.* Curr Med Res Opin, 2016. **32**(9): p. 1547-56.

205. Katzmann, J., et al., *Behavioral and Nondirective Guided Self-Help for Parents of Children with Externalizing Behavior: Mediating Mechanisms in a Head-To-Head Comparison.* Journal of abnormal child psychology, 2017. **45**(4): p. 719-730.

206. Keedwell, P.A. and A.H. Young, *Emerging drugs for bipolar depression: An update.* Expert Opinion on Emerging Drugs, 2014. **19**(1): p. 25-36.

207. Keith, S., *Use of long-acting risperidone in psychiatric disorders: Focus on efficacy, safety and cost-effectiveness.* Expert Review of Neurotherapeutics, 2009. **9**(1): p. 9-31.

208. Kelbrick, M., S. Abu-Kmeil, and M. Picchioni, *Evaluating outcomes in an adult inpatient psychiatric rehabilitation unit.* Progress in Neurology and Psychiatry, 2016. **20**(5): p. 18-24.

209. Kelley, B.J., *Treatment of Mild Cognitive Impairment.* Current Treatment Options in Neurology, 2015. **17**(9).

210. Kelly, J.F., K. Humphreys, and M. Ferri, *Alcoholics Anonymous and other 12-step programs for alcohol use disorder.* Cochrane Database of Systematic Reviews, 2017. **2017**(11).

211. Kelly, M.J., et al., *Evaluating cutpoints for the MHI-5 and MCS using the GHQ-12: a comparison of five different methods.* BMC psychiatry, 2008. **8**: p. 10.

212. Kemmeren, L.L., et al., *Effectiveness of blended depression treatment for adults in specialised mental healthcare: Study protocol for a randomised controlled trial.* BMC Psychiatry, 2016. **16**(1).

213. Kendall, T., et al., *Borderline and antisocial personality disorders: Summary of NICE guidance.* BMJ (Online), 2009. **338**(7689): p. 293-295.

214. Kendall-Raynor, P., *Healthcare innovations could save NHS billions of pounds.* Nurs Older People, 2017. **29**(6): p. 6.

215. Kenter, R.M., et al., *Effectiveness and cost effectiveness of guided online treatment for patients with major depressive disorder on a waiting list for psychotherapy: study protocol of a randomized controlled trial.* Trials, 2013. **14**: p. 412.

216. Kenter, R.M.F., et al., *Costs and effects of Internet cognitive behavioral treatment blended with face-to-face treatment: Results from a naturalistic study.* Internet Interventions, 2015. **2**(1): p. 77-83.

217. Kenter, R.M.F., et al., *Effectiveness and cost effectiveness of guided online treatment for patients with major depressive disorder on a waiting list for psychotherapy: Study protocol of a randomized controlled trial.* Trials, 2013. **14**(1).

218. Kenworthy, J., et al., *Use of opioid substitution therapies in the treatment of opioid use disorder: results of a UK cost-effectiveness modelling study.* Journal of Medical Economics, 2017. **20**(7): p. 740-748.

219. Kerkemeyer, L., et al., *Evaluation of an integrated care program for schizophrenia: concept and study design.* Eur Arch Psychiatry Clin Neurosci, 2015. **265**(2): p. 155-62.

220. Kiencke, P., et al., *Direct costs of Alzheimer's disease in Germany.* The European Journal of Health Economics, 2011. **12**(6): p. 533-539.

221. Killaspy, H., et al., *Study protocol: cluster randomised controlled trial to assess the clinical and cost effectiveness of a staff training intervention in inpatient mental health rehabilitation units in increasing service users' engagement in activities.* BMC Psychiatry, 2013. **13**: p. 216.

222. Killaspy, H., et al., *Ten year outcomes of participants in the REACT (Randomised Evaluation of Assertive Community Treatment in North London) study.* BMC Psychiatry, 2014. **14**: p. 296.

223. Kim, E., et al., *One-year risk of psychiatric hospitalization and associated treatment costs in bipolar disorder treated with atypical antipsychotics: A retrospective claims database analysis.* BMC Psychiatry, 2011. **11**.

224. Kleiboer, A., et al., *European COMPARative Effectiveness research on blended Depression treatment versus treatment-as-usual (E-COMPARED): study protocol for a randomized controlled, non-inferiority trial in eight European countries.* Trials, 2016. **17**(1): p. 387.

225. Klein, J., et al., *Guideline-adherent inpatient psychiatric psychotherapeutic treatment of obsessive-compulsive disorder. Normative definition of personnel requirements.* Der Nervenarzt, 2016. **87**(7): p. 731-738.

226. Kleine-Budde, K., et al., *Cost of illness for bipolar disorder: a systematic review of the economic burden.* Bipolar Disord, 2014. **16**(4): p. 337-53.

227. Klug, G., et al., *Effectiveness of home treatment for elderly people with depression: randomised controlled trial.* Br J Psychiatry, 2010. **197**(6): p. 463-7.

228. Knapp, M., *Preschool hyperactivity increases costs into early adulthood.* Evidence-based mental health, 2016. **19**(1): p. 30.

229. Knapp, M., et al., *How do child and adolescent mental health problems influence public sector costs? Interindividual variations in a nationally representative British sample.* Journal of child psychology and psychiatry, and allied disciplines, 2015. **56**(6): p. 667-676.

230. Knowles, S.E., et al., *Patient experience of computerised therapy for depression in primary care.* BMJ Open, 2015. **5**(11): p. e008581.

231. Koch, A., et al., *MEMENTA-'Mental healthcare provision for adults with intellectual disability and a mental disorder'. A cross-sectional epidemiological multisite study assessing prevalence of psychiatric symptomatology, needs for care and quality of healthcare provision for adults with intellectual disability in Germany: A study protocol.* BMJ Open, 2014. **4**(5).

232. Koeser, L., et al., *Economic evaluation of audio based resilience training for depression in primary care.* Journal of Affective Disorders, 2013. **149**(1-3): p. 307-312.

233. Koeser, L., et al., *Modelling the cost-effectiveness of pharmacotherapy compared with cognitive-behavioural therapy and combination therapy for the treatment of moderate to severe depression in the UK.* Psychol Med, 2015. **45**(14): p. 3019-31.

234. Kok, G., et al., *Current treatment of depression is a waste of money.* Psychologie & Gezondheid, 2011. **39**(1): p. 26-31.

235. Kok, L., C. Berden, and K. Sadiraj, *Costs and benefits of home care for the elderly versus residential care: a comparison using propensity scores.* Eur J Health Econ, 2015. **16**(2): p. 119-31.

236. Kok, R.N., et al., *Adherence to a web-based pre-treatment for phobias in outpatient clinics.* Internet Interventions, 2017. **9**: p. 38-45.

237. Kok, R.N., et al., *Effectiveness and cost-effectiveness of web-based treatment for phobic outpatients on a waiting list for psychotherapy: protocol of a randomised controlled trial.* BMC Psychiatry, 2012. **12**: p. 131.

238. Koller, M., *[BPSD: behavioural and psychological symptoms of dementia].* MMW Fortschr Med, 2011. **153**(14): p. 54-7.

239. Kollner, V. and A. Maercker, *Diagnostic categories for PTSD and other stress-response disorders.* Trauma & Gewalt, 2011. **5**(3): p. 236-247.

240. Kolovos, S., et al., *Cost effectiveness of guided Internet-based interventions for depression in comparison with control conditions: An individual–participant data meta-analysis.* Depression and Anxiety, 2018. **35**(3): p. 209-219.

241. Konig, H.H., et al., *[How does the Regional Psychiatry Budget (RPB) work in an area with initially low capacity of psychiatric hospital beds?].* Psychiatr Prax, 2013. **40**(8): p. 430-8.

242. Konnopka, A., et al., *The cost of schizophrenia in Germany: A systematic review of the literature.* Psychiatrische Praxis, 2009. **36**(5): p. 211-218.

243. Konnopka, A., et al., *Cost-of-illness studies and cost-effectiveness analyses in anxiety disorders: a systematic review.* J Affect Disord, 2009. **114**(1-3): p. 14-31.

244. Konnopka, A., et al., *Economics of medically unexplained symptoms: a systematic review of the literature.* Psychother Psychosom, 2012. **81**(5): p. 265-75.

245. Kooij, S.J.J., et al., *European consensus statement on diagnosis and treatment of adult ADHD: The European Network Adult ADHD.* BMC Psychiatry, 2010. **10**.

246. Kooistra, L.C., et al., *Blended vs. Face-to-face cognitive behavioural treatment for major depression in specialized mental health care: Study protocol of a randomized controlled cost-effectiveness trial.* BMC Psychiatry, 2014. **14**(1): p. 1-11.

247. Korfage, I.J., et al., *A cluster randomized controlled trial on the effects and costs of advance care planning in elderly care: study protocol.* BMC Geriatr, 2015. **15**: p. 87.

248. Korte, J., E.T. Bohlmeijer, and F. Smit, *Prevention of depression and anxiety in later life: design of a randomized controlled trial for the clinical and economic evaluation of a life-review intervention.* BMC Public Health, 2009. **9**: p. 250.

249. Kortmann, L.M., et al., *[Disability Pension and Productivity Loss in Schizophrenia - An Empirical Analysis of the Financial Burden in Germany].* Psychiatr Prax, 2017. **44**(2): p. 93-98.

250. Kotsopoulos, N., et al., *The fiscal consequences of ADHD in Germany: A quantitative analysis based on differences in educational attainment and lifetime earnings.* Journal of Mental Health Policy and Economics, 2013. **16**(1): p. 27-33.

251. Kotz, D., et al., *How cost-effective is 'No Smoking Day'?* Tob Control, 2011. **20**(4): p. 302-4.

252. Kouimtsidis, C., et al., *A feasibility randomised controlled trial of extended brief intervention for alcohol misuse in adults with mild to moderate intellectual disabilities living in the community; The EBI-LD study.* Trials, 2017. **18**(1): p. 216.

253. Krishnan, S., R. Cairns, and R. Howard, *Cannabinoids for the treatment of dementia.* Cochrane Database of Systematic Reviews, 2009(2).

254. Kruckenberg, P., et al., *[Psychiatric psychotherapeutic psychosomatic treatment by the hospital: framework for the development of a multi-sector budget for regional mandatory care].* Psychiatr Prax, 2009. **36**(5): p. 246-9.

255. Kruisdijk, F.R., et al., *Effect of running therapy on depression (EFFORT-D). Design of a randomised controlled trial in adult patients [ISRCTN 1894].* BMC public health, 2012. **12**: p. 50.

256. Kuyken, W., et al., *The effectiveness and cost-effectiveness of a mindfulness training programme in schools compared with normal school provision (MYRIAD): study protocol for a randomised controlled trial.* Trials, 2017. **18**(1): p. 194.

257. Kvarstein, E.H., et al., *Health service costs and clinical gains of psychotherapy for personality disorders: A randomized controlled trial of day-hospital-based step-down treatment versus outpatient treatment at a specialist practice.* BMC Psychiatry, 2013. **13**.

258. Labrador, F.J., et al., *Treatment of Anxiety Disorders in a Psychology Clinic.* The Spanish journal of psychology, 2015. **18**: p. E83.

259. Lagerveld SE, B.R., Brenninkmeijer V, Wijngaards-de Meij L, Schaufeli WB, *Work-focused treatment of common mental disorders and return to work: a comparative outcome study.* Journal of Occupational Health Psychology, 2012. **17**(2): p. 220-234.

260. Lamb, C.E., *Alternatives to admission for children and adolescents: Providing intensive mental healthcare services at home and in communities: What works?* Current Opinion in Psychiatry, 2009. **22**(4): p. 345-350.

261. Lambert, M., et al., *Early detection and integrated care for adolescents and young adults with severe psychotic disorders: rationales and design of the Integrated Care in Early Psychosis Study (ACCESS III).* Early Intervention in Psychiatry, 2018. **12**(1): p. 96-106.

262. Lamph, G. and E. Hickey, *An inclusive approach to personality disorders.* Nursing times, 2012. **108**(39): p. 18-20.

263. Lancee, J., et al., *Guided online or face-to-face cognitive behavioral treatment for insomnia: A randomized wait-list controlled trial.* Sleep, 2016. **39**(1): p. 183-191.

264. Laporte Uribe, F., et al., *Regional dementia care networks in Germany: changes in caregiver burden at one-year follow-up and associated factors.* Int Psychogeriatr, 2017. **29**(6): p. 991-1004.

265. Laramee, P., et al., *The cost-effectiveness and public health benefit of nalmefene added to psychosocial support for the reduction of alcohol consumption in alcohol-dependent patients with high/very high drinking risk levels: a Markov model.* BMJ Open, 2014. **4**(9): p. e005376.

266. Laurenssen, E.M., et al., *Day hospital Mentalization-based treatment versus intensive outpatient Mentalization-based treatment for patients with severe borderline personality disorder: protocol of a multicentre randomized clinical trial.* BMC Psychiatry, 2014. **14**: p. 301.

267. Laurenssen, E.M., et al., *Day Hospital Mentalization-Based Treatment (MBT-DH) versus treatment as usual in the treatment of severe borderline personality disorder: protocol of a randomized controlled trial.* BMC Psychiatry, 2014. **14**: p. 149.

268. Le, H.H., et al., *Economic impact of childhood/adolescent ADHD in a European setting: The Netherlands as a reference case.* European Child & Adolescent Psychiatry, 2014. **23**(7): p. 587-598.

269. Lehnert, T., et al., *Health economic aspects of physical-mental comorbidity.* Bundesgesundheitsblatt - Gesundheitsforschung - Gesundheitsschutz, 2011. **54**(1): p. 120-127.

270. Leitner, A., et al., *[Is there adequate care for patients with psychosomatic disorders in Austria? Analysis of the need and a proposal for a model of quality assurance in Austrian psychosomatic medicine].* Z Psychosom Med Psychother, 2013. **59**(4): p. 408-21.

271. Lemmens, L.H., et al., *Effectiveness, relapse prevention and mechanisms of change of cognitive therapy vs. interpersonal therapy for depression: Study protocol for a randomised controlled trial.* Trials, 2011. **12**: p. 150.

272. Lenox-Smith, A., et al., *Cost effectiveness of venlafaxine compared with generic fluoxetine or generic amitriptyline in major depressive disorder in the UK.* Clin Drug Investig, 2009. **29**(3): p. 173-84.

273. Lensberg, B.R., et al., *Challenges in measuring and valuing productivity costs, and their relevance in mood disorders.* ClinicoEconomics and Outcomes Research, 2013. **5**(1): p. 565-573.

274. Lever Taylor, B., et al., *The effectiveness of self-help mindfulness-based cognitive therapy ina student sample: A randomised controlled trial.* Behaviour Research and Therapy, 2014. **63**: p. 63-69.

275. Lewis, F.I. and P.R. Torgerson, *The current and future burden of late-onset dementia in the United Kingdom: Estimates and interventions.* Alzheimers Dement, 2017. **13**(1): p. 38-44.

276. Li, S.C. and S.K. Aggarwal, *Estimation of resource utilisation difference between lithium and valproate treatment groups from the VALID study.* Journal of Medical Economics, 2011. **14**(3): p. 350-356.

277. Linden, M., et al., *The best next drug in the course of generalized anxiety disorders: The "pN-GAD-algorithm".* International Journal of Psychiatry in Clinical Practice, 2013. **17**(2): p. 78-89.

278. Lintzeris, N., *Prescription of heroin for the management of heroin dependence: Current status.* CNS Drugs, 2009. **23**(6): p. 463-476.

279. Livingston, G., et al., *A systematic review of the clinical effectiveness and cost-effectiveness of sensory, psychological and behavioural interventions for managing agitation in older adults with dementia.* Health Technol Assess, 2014. **18**(39): p. 1-226, v-vi.

280. Liwinski, T., G. Romer, and J.M. Muller, *[Evaluation of Treatment of Mothers at the Family Day Hospital in Munster, Germany].* Prax Kinderpsychol Kinderpsychiatr, 2015. **64**(4): p. 254-72.

281. Lloyd, A., et al., *Estimation of utilities in attention-deficit hyperactivity disorder for economic evaluations.* Patient, 2011. **4**(4): p. 247-57.

282. Lloyd, M., *Reducing the cost of dissociative identity disorder: Measuring the effectiveness of specialized treatment by frequency of contacts with mental health services.* J Trauma Dissociation, 2016. **17**(3): p. 362-70.

283. Lobban, F., et al., *Assessing Feasibility and Acceptability of Web-Based Enhanced Relapse Prevention for Bipolar Disorder (ERPonline): A Randomized Controlled Trial.* Journal of medical Internet research, 2017. **19**(3): p. e85.

284. Lobban, F., et al., *Protocol for an online randomised controlled trial to evaluate the clinical and cost-effectiveness of a peer-supported self-management intervention for relatives of people with psychosis or bipolar disorder: Relatives Education And Coping Toolkit (REACT).* BMJ Open, 2017. **7**(7): p. e016965.

285. Lock, J., J. Couturier, and W.S. Agras, *Costs of remission and recovery using family therapy for adolescent anorexia nervosa: A descriptive report.* Eating Disorders, 2008. **16**(4): p. 322-330.

286. Lokkerbol, J., et al., *Improving the cost-effectiveness of a healthcare system for depressive disorders by implementing telemedicine: A health economic modeling study.* American Journal of Geriatric Psychiatry, 2014. **22**(3): p. 253-262.

287. Lokkerbol, J., et al., *Non-fatal burden of disease due to mental disorders in the Netherlands.* Soc Psychiatry Psychiatr Epidemiol, 2013. **48**(10): p. 1591-9.

288. Lokkerbol, J., et al., *Mental health care system optimization from a health-economics perspective: Where to sow and where to reap?* Journal of Mental Health Policy and Economics, 2014. **17**(2): p. 51-60.

289. Long, C.G., et al., *Group substance abuse treatment for women in secure services.* Mental Health and Substance Use: Dual Diagnosis, 2010. **3**(3): p. 227-237.

290. Lopez-Bastida, J., et al., *Cost-effectiveness of donepezil in the treatment of mild or moderate Alzheimer's disease.* Journal of Alzheimer's Disease, 2009. **16**(2): p. 399-407.

291. Lopez-Castroman, J., et al., *Cost-efficiency of laboratory testing among psychiatric inpatients.* Int J Psychiatry Med, 2012. **44**(3): p. 211-24.

292. López-García-Franco, A., et al., *Effectiveness of a cognitive behavioral intervention in patients with medically unexplained symptoms: cluster randomized trial.* BMC family practice, 2012. **13**: p. 35.

293. Lopez-Pousa, S., et al., *[The effect of donepezil in comparison with conventional treatment on cognitive functioning and the performance of the patient in a prospective cohort of patients with Alzheimer's disease treated in routine clinical practice in Spain].* Rev Neurol, 2010. **51**(10): p. 577-88.

294. Lovell, K., et al., *Development and evaluation of culturally sensitive psychosocial interventions for under-served people in primary care.* BMC Psychiatry, 2014. **14**: p. 217.

295. Lueke, S., W. Hoffmann, and S. Flebetaa, *Transitions between care settings in dementia: Are they relevant in economic terms?* Value in Health, 2014. **17**(6): p. 679-685.

296. Luman, M., A. Papanikolau, and J. Oosterlaan, *The Unique and Combined Effects of Reinforcement and Methylphenidate on Temporal Information Processing in Attention-Deficit/Hyperactivity Disorder.* Journal of Clinical Psychopharmacology, 2015. **35**(4): p. 414-421.

297. Luppa, M., et al., *Health service utilization and costs of depressive symptoms in late life - a systematic review.* Curr Pharm Des, 2012. **18**(36): p. 5936-57.

298. Lynch, T.R., et al., *Refractory depression: Mechanisms and evaluation of radically open dialectical behaviour therapy (RO-DBT) [REFRAMED]: Protocol for randomised trial.* BMJ Open, 2015. **5**(7).

299. Lyon, K., *A new approach to alcohol misuse.* Nurs Stand, 2016. **30**(52): p. 18-21.

300. Mac Giolla Phadraig, C., et al., *Should we provide oral health training for staff caring for people with intellectual disabilities in community based residential care? A cost-effectiveness analysis.* Evaluation and program planning, 2016. **55**: p. 46-54.

301. Macaluso, M., H. Oliver, and Z. Sohail, *Pharmacokinetic drug evaluation of paliperidone in the treatment of schizoaffective disorder.* Expert Opinion on Drug Metabolism and Toxicology, 2017. **13**(8): p. 871-879.

302. Mackin, P. and S.H.L. Thomas, *Atypical antipsychotic drugs.* BMJ, 2011. **342**(7798): p. 650-653.

303. MacPherson, H., et al., *Acupuncture, Counseling, and Usual care for Depression (ACUDep): Study protocol for a randomized controlled trial.* Trials, 2012. **13**.

304. Magallón, R., et al., *Cognitive-behaviour therapy for patients with Abridged Somatization Disorder (SSI 4,6) in primary care: A randomized, controlled study.* BMC Psychiatry, 2008. **8**.

305. Magnezi, R., et al., *Comparison between neurostimulation techniques repetitive transcranial magnetic stimulation vs electroconvulsive therapy for the treatment of resistant depression: Patient preference and cost-effectiveness.* Patient Preference and Adherence, 2016. **10**: p. 1481-1487.

306. Maguire, T., *Legal action against cuts will only delay the inevitable.* Pharmaceutical Journal, 2017. **298**(7899): p. 160-162.

307. Maljanen, T., et al., *The cost-effectiveness of short-term and long-term psychotherapy in the treatment of depressive and anxiety disorders during a 5-year follow-up.* Journal of Affective Disorders, 2016. **190**: p. 254-263.

308. Marchand, A., et al., *A randomized, controlled clinical trial of standard, group and brief cognitive-behavioral therapy for panic disorder with agoraphobia: A two-year follow-up.* Journal of Anxiety Disorders, 2009. **23**(8): p. 1139-1147.

309. Martin-Carrasco, M., et al., *Effectiveness of a psychoeducational intervention group program in the reduction of the burden experienced by caregivers of patients with dementia: the EDUCA-II randomized trial.* Alzheimer Dis Assoc Disord, 2014. **28**(1): p. 79-87.

310. Martin-Carrasco, M., et al., *A randomized trial to assess the efficacy of a psychoeducational intervention on caregiver burden in schizophrenia.* Eur Psychiatry, 2016. **33**: p. 9-17.

311. Martin-Carrasco, M., et al., *Effectiveness of a psychoeducational intervention program in the reduction of caregiver burden in Alzheimer's disease patients' caregivers.* Int J Geriatr Psychiatry, 2009. **24**(5): p. 489-99.

312. Martin-Carrasco, M., et al., *EDUCA study: Psychometric properties of the Spanish version of the Zarit Caregiver Burden Scale.* Aging Ment Health, 2010. **14**(6): p. 705-11.

313. Martinez-Raga, J., et al., *Suboxone (Buprenorphine/Naloxone) as an agonist opioid treatment in Spain: A budgetary impact analysis.* European Addiction Research, 2010. **16**(1): p. 31-42.

314. Martorell, A., et al., *Family impact in intellectual disability, severe mental health disorders and mental health disorders in ID. A comparison.* Res Dev Disabil, 2011. **32**(6): p. 2847-52.

315. Marwood, L., et al., *Study protocol for a randomised pragmatic trial comparing the clinical and cost effectiveness of lithium and quetiapine augmentation in treatment resistant depression (the LQD study).* BMC Psychiatry, 2017. **17**(1).

316. Massoudi, B., et al., *Blended care vs. usual care in the treatment of depressive symptoms and disorders in general practice [BLENDING]: study protocol of a non-inferiority randomized trial.* BMC Psychiatry, 2017. **17**(1): p. 218.

317. Matheson, L., et al., *Adult ADHD patient experiences of impairment, service provision and clinical management in England: a qualitative study.* BMC Health Serv Res, 2013. **13**: p. 184.

318. Matza, L.S., et al., *Health state utilities associated with adult attention-deficit/hyperactivity disorder.* Patient Preference and Adherence, 2014. **8**: p. 997-1006.

319. Matza, L.S., et al., *Validation of a patient interview for assessing reasons for antipsychotic discontinuation and continuation.* Patient Preference and Adherence, 2012. **6**: p. 521-532.

320. Mavranezouli, I. and J. Lokkerbol, *A Systematic Review and Critical Appraisal of Economic Evaluations of Pharmacological Interventions for People with Bipolar Disorder.* Pharmacoeconomics, 2017. **35**(3): p. 271-296.

321. Mavranezouli, I., et al., *The Cost Effectiveness of Psychological and Pharmacological Interventions for Social Anxiety Disorder: A Model-Based Economic Analysis.* PLoS One, 2015. **10**(10): p. e0140704.

322. Mavranezouli, I., et al., *The cost effectiveness of pharmacological treatments for generalized anxiety disorder.* PharmacoEconomics, 2013. **31**(4): p. 317-333.

323. Mavranezouli, I., et al., *The cost-effectiveness of supported employment for adults with autism in the United Kingdom.* Autism, 2014. **18**(8): p. 975-984.

324. Mayer, S., et al., *Health-Related Resource-Use Measurement Instruments for Intersectoral Costs and Benefits in the Education and Criminal Justice Sectors.* PharmacoEconomics, 2017. **35**(9): p. 895-908.

325. McCann, D.C., et al., *Study protocol for a randomized controlled trial comparing the efficacy of a specialist and a generic parenting programme for the treatment of preschool ADHD.* Trials, 2014. **15**: p. 142.

326. McClay, C.A., et al., *A community-based group-guided self-help intervention for low mood and stress: study protocol for a randomized controlled trial.* Trials, 2013. **14**: p. 392.

327. McCrone, P., *Does early intervention for psychosis services make economic sense?* Ment Health Today, 2012: p. 30-3.

328. McCrone, P., M. Knapp, and S. Dhanasiri, *Economic impact of services for first-episode psychosis: A decision model approach.* Early Intervention in Psychiatry, 2009. **3**(4): p. 266-273.

329. McCrone, P., et al., *The economic impact of initiatives to reduce stigma: demonstration of a modelling approach.* Epidemiol Psichiatr Soc, 2010. **19**(2): p. 131-9.

330. McCrone, P., et al., *The economic impact of early intervention in psychosis services for children and adolescents.* Early Interv Psychiatry, 2013. **7**(4): p. 368-73.

331. McGill, P. and J. Poynter, *High Cost Residential Placements for Adults with Intellectual Disabilities.* Journal of Applied Research in Intellectual Disabilities, 2012. **25**(6): p. 584-587.

332. McIntyre, R.S., et al., *Major depressive disorder with subthreshold hypomanic (mixed) features: A real-world assessment of treatment patterns and economic burden.* Journal of Affective Disorders, 2017. **210**: p. 332-337.

333. McKay, J.R., et al., *Effects of automated smartphone mobile recovery support and telephone continuing care in the treatment of alcohol use disorder: Study protocol for a randomized controlled trial.* Trials, 2018. **19**(1).

334. McLeod, H., et al., *Effect of pay-for-outcomes and encouraging new providers on national health service smoking cessation services in England: a cluster controlled study.* PLoS One, 2015. **10**(4): p. e0123349.

335. McMurran, M., et al., *The addition of a goal-based motivational interview to standardised treatment as usual to reduce dropouts in a service for patients with personality disorder: A feasibility study.* Trials, 2010. **11**.

336. McNamara, R., et al., *A pilot randomised controlled trial of community-led antipsychotic drug reduction for adults with learning disabilities.* Health Technology Assessment, 2017. **21**(47): p. 1-122.

337. Meeuwsen, E.J., et al., *Cost-effectiveness of post-diagnosis treatment in dementia coordinated by Multidisciplinary Memory Clinics in comparison to treatment coordinated by general practitioners: an example of a pragmatic trial.* J Nutr Health Aging, 2009. **13**(3): p. 242-8.

338. Meeuwsen, E.J., et al., *Effectiveness of dementia follow-up care by memory clinics or general practitioners: randomised controlled trial.* Bmj, 2012. **344**: p. e3086.

339. Mellentin, A.I., et al., *A randomized controlled study of exposure therapy as aftercare for alcohol use disorder: Study protocol.* BMC Psychiatry, 2016. **16**(1).

340. Mengoni, S.E., et al., *Feasibility study of a randomised controlled trial to investigate the effectiveness of using a humanoid robot to improve the social skills of children with autism spectrum disorder (Kaspar RCT): A study protocol.* BMJ Open, 2017. **7**(6).

341. Mennini, F.S., et al., *Pilot evaluation of indirect costs and the impact of bipolar disorder type I.* Journal of Psychopathology, 2014. **20**(2): p. 216-222.

342. Meppelink, R., E.I. de Bruin, and S.M. Bögels, *Meditation or Medication? Mindfulness training versus medication in the treatment of childhood ADHD: A randomized controlled trial.* BMC Psychiatry, 2016. **16**(1).

343. Meston, C.M., T.A. Lorenz, and K.R. Stephenson, *Effects of expressive writing on sexual dysfunction, depression, and PTSD in women with a history of childhood sexual abuse: Results from a randomized clinical trial.* Journal of Sexual Medicine, 2013. **10**(9): p. 2177-2189.

344. Meuldijk, D., et al., *A randomized controlled trial of the efficacy and cost-effectiveness of a brief intensified cognitive behavioral therapy and/or pharmacotherapy for mood and anxiety disorders: Design and methods.* Contemporary Clinical Trials, 2012. **33**(5): p. 983-992.

345. Meulenbeek, P., et al., *Early intervention in panic: Randomized controlled trial and cost-effectiveness analysis.* Trials, 2008. **9**.

346. Meyers, J., et al., *The impact of adjunctive guanfacine extended release on stimulant adherence in children/adolescents with attention-deficit/hyperactivity disorder.* Journal of Comparative Effectiveness Research, 2017. **6**(2): p. 109-125.

347. Michelson, D., et al., *Early intervention for depression and anxiety in 16-18-year-olds: Protocol for a feasibility cluster randomised controlled trial of open-access psychological workshops in schools (DISCOVER).* Contemporary Clinical Trials, 2016. **48**: p. 52-58.

348. Mihalopoulos, C., et al., *Assessing outcomes for cost-utility analysis in depression: Comparison of five multi-attribute utility instruments with two depression-specific outcome measures.* British Journal of Psychiatry, 2014. **205**(5): p. 390-397.

349. Mihalopoulos, C., et al., *Is early intervention in psychosis cost-effective over the long term?* Schizophrenia Bulletin, 2009. **35**(5): p. 909-918.

350. Millier, A., et al., *Cost-Effectiveness of Nalmefene Added to Psychosocial Support for the Reduction of Alcohol Consumption in Alcohol-Dependent Patients With High/Very High Drinking Risk Levels: A Microsimulation Model.* J Stud Alcohol Drugs, 2017. **78**(6): p. 867-876.

351. Minsky, S., et al., *Service use among patients with serious mental illnesses who presented with physical symptoms at intake.* Psychiatric Services, 2011. **62**(10): p. 1146-1151.

352. Mitchell, P.M., et al., *Assessing the validity of the ICECAP-A capability measure for adults with depression.* BMC Psychiatry, 2017. **17**(1).

353. Moessner, M., et al., *Effectiveness and Cost-effectiveness of School-based Dissemination Strategies of an Internet-based Program for the Prevention and Early Intervention in Eating Disorders: A Randomized Trial.* Prev Sci, 2016. **17**(3): p. 306-13.

354. Mohseninejad, L., et al., *Value of information analysis from a societal perspective: a case study in prevention of major depression.* Value Health, 2013. **16**(4): p. 490-7.

355. Monsalve, M., et al., *Identification and characterization of the cost related to the patient care with bipolar i disorder receiving pharmaceutical care.* Vitae, 2015. **22**: p. S114-S118.

356. Moran, P., et al., *The effectiveness of joint crisis plans for people with borderline personality disorder: personalityrotocol for an exploratory randomised controlled trial.* Trials, 2010. **11**.

357. Morley, M. and G. Smyth, *Are occupational therapy interventions for service users with mental health problems cost-effective?* The British Journal of Occupational Therapy, 2013. **76**(10): p. 470-473.

358. Morriss, R., et al., *Persistent frequent attenders in primary care: costs, reasons for attendance, organisation of care and potential for cognitive behavioural therapeutic intervention.* BMC family practice, 2012. **13**: p. 39.

359. Morriss, R., et al., *Randomised controlled trial of the clinical and cost effectiveness of a specialist team for managing refractory unipolar depressive disorder.* BMC Psychiatry, 2010. **10**.

360. Morriss, R.K., et al., *Pragmatic randomised controlled trial of group psychoeducation versus group support in the maintenance of bipolar disorder.* BMC Psychiatry, 2011. **11**.

361. Morrissey, J.P., M.E. Domino, and G.S. Cuddeback, *Assessing the effectiveness of recovery-oriented ACT in reducing state psychiatric hospital use.* Psychiatric Services, 2013. **64**(4): p. 303-311.

362. Motzek, T., M. Junge, and G. Marquardt, *[Impact of dementia on length of stay and costs in acute care hospitals].* Z Gerontol Geriatr, 2017. **50**(1): p. 59-66.

363. Mukuria, C. and J. Brazier, *Valuing the EQ-5D and the SF-6D health states using subjective well-being: A secondary analysis of patient data.* Social Science and Medicine, 2013. **77**(1): p. 97-105.

364. Murphy, S.M., et al., *An evaluation of the effectiveness and cost effectiveness of the National Exercise Referral Scheme in Wales, UK: A randomised controlled trial of a public health policy initiative.* Journal of Epidemiology and Community Health, 2012. **66**(8): p. 745-753.

365. Nagtegaal, M.H., K. Goethals, and G. Meynen, *Mandatory treatment of forensic psychiatric patients in the Netherlands: Costs and benefits in perspective.* Tijdschrift voor Psychiatrie, 2016. **58**(10): p. 739-745.

366. Nagy, B., et al., *Assessing the cost-effectiveness of the rivastigmine transdermal patch for Alzheimer's disease in the UK using MMSE- and ADL-based models.* Int J Geriatr Psychiatry, 2011. **26**(5): p. 483-94.

367. Nieboer, A.P., X. Koolman, and E.A. Stolk, *Preferences for long-term care services: Willingness to pay estimates derived from a discrete choice experiment.* Social Science & Medicine, 2010. **70**(9): p. 1317-1325.

368. Nijhof, N., et al., *A personal assistant for dementia to stay at home safe at reduced cost.* Gerontechnology, 2013. **11**(3): p. 469-479.

369. Nilsson, M.E., et al., *Postdischarge interventions for depression.* Cochrane Database of Systematic Reviews, 2015. **2015**(3).

370. Noordik, E., et al., *Effectiveness and cost-effectiveness of an exposure-based return-to-work programme for patients on sick leave due to common mental disorders: design of a cluster-randomized controlled trial.* BMC Public Health, 2009. **9**: p. 140.

371. Noordraven, E.L., et al., *Money for medication: A randomized controlled study on the effectiveness of financial incentives to improve medication adherence in patients with psychotic disorders.* BMC Psychiatry, 2014. **14**(1).

372. Nordgren, L.B., et al., *Effectiveness and cost-effectiveness of individually tailored Internet-delivered cognitive behavior therapy for anxiety disorders ina primary care population: A randomized controlled trial.* Behaviour Research and Therapy, 2014. **59**: p. 1-11.

373. Normann, C., et al., *Resource use and financing of guideline-adherent psychotherapeutic inpatient care.* Der Nervenarzt, 2015. **86**(5): p. 534-541.

374. Northen, S., *Behind closed doors.* Nursing standard (Royal College of Nursing (Great Britain) : 1987), 2008. **22**(35): p. 16-19.

375. Nuijten, M.J.C., et al., *Cost-Effectiveness of Escitalopram in Major Depressive Disorder in the Dutch Health Care Setting.* Clinical Therapeutics, 2012. **34**(6): p. 1364-1377.

376. O'Neill, S., F. Ferry, and D. Heenan, *Mental health disorders in Northern Ireland: the economic imperative.* Lancet Psychiatry, 2016. **3**(5): p. 398-400.

377. Oddy, M. and S. Da Silva Ramos, *Cost effective ways of facilitating home based rehabilitation and support.* NeuroRehabilitation, 2013. **32**(4): p. 781-790.

378. Ohlsen, R.I., et al., *Returning to the issue of the cost-effectiveness of antipsychotics in the treatment of Schizophrenia.* Clinical Neuropsychiatry, 2008. **5**(4): p. 184-194.

379. Oliva-Moreno, J., *Loss of labour productivity caused by disease and health problems: What is the magnitude of its effect on Spain's Economy?* European Journal of Health Economics, 2012. **13**(5): p. 605-614.

380. Oliva-Moreno, J., L.M. Pena-Longobardo, and C. Vilaplana-Prieto, *An Estimation of the Value of Informal Care Provided to Dependent People in Spain.* Applied Health Economics and Health Policy, 2015. **13**(2): p. 223-231.

381. Oono, I.P., E.J. Honey, and H. McConachie, *Parent-mediated early intervention for young children with autism spectrum disorders (ASD).* Evidence-Based Child Health, 2013. **8**(6): p. 2380-2479.

382. Oostveen, R., et al., *Effectiveness of pharmacotherapy in behavioural therapeutic smoking cessation programmes.* Eur J Public Health, 2015. **25**(2): p. 204-9.

383. Ophuis, R.H., et al., *Cost-effectiveness of interventions for treating anxiety disorders: A systematic review.* J Affect Disord, 2017. **210**: p. 1-13.

384. Osborne, A., *Future delivery of the Drug Interventions Programme: do the benefits justify the costs?* J Forensic Leg Med, 2013. **20**(7): p. 816-20.

385. Ostad Haji, E., C. Hiemke, and B. Pfuhlmann, *Therapeutic drug monitoring for antidepressant drug treatment.* Curr Pharm Des, 2012. **18**(36): p. 5818-27.

386. Ostad Haji, E., et al., *Potential cost-effectiveness of therapeutic drug monitoring for depressed patients treated with citalopram.* Ther Drug Monit, 2013. **35**(3): p. 396-401.

387. Overend, K., et al., *CASPER plus (CollAborative care in Screen-Positive EldeRs with major depressive disorder): Study protocol for a randomised controlled trial.* Trials, 2014. **15**(1).

388. Page, T.F., et al., *Comparative Cost Analysis of Sequential, Adaptive, Behavioral, Pharmacological, and Combined Treatments for Childhood ADHD.* Journal of clinical child and adolescent psychology : the official journal for the Society of Clinical Child and Adolescent Psychology, American Psychological Association, Division 53, 2016. **45**(4): p. 416-427.

389. Pan, Y.J., M. Knapp, and P. McCrone, *Cost-effectiveness comparisons between antidepressant treatments in depression: evidence from database analyses and prospective studies.* J Affect Disord, 2012. **139**(2): p. 113-25.

390. Parés-Badell, O., et al., *Cost of disorders of the brain in Spain.* PLoS ONE, 2014. **9**(8).

391. Park, A.L., P. McCrone, and M. Knapp, *Early intervention for first-episode psychosis: Broadening the scope of economic estimates.* Early Intervention in Psychiatry, 2016. **10**(2): p. 144-151.

392. Park, A.L., et al., *Examining the cost effectiveness of interventions to promote the physical health of people with mental health problems: a systematic review.* BMC public health, 2013. **13**: p. 787.

393. Parry, S.W., et al., *Cognitive–behavioural therapy-based intervention to reduce fear of falling in older people: Therapy development and randomised controlled trial – the strategies for increasing independence, confidence and energy (STRIDE) study.* Health Technology Assessment, 2016. **20**(56): p. 1-206.

394. Patel, A., *The cost of mood disorders.* Psychiatry, 2009. **8**(2): p. 76-80.

395. Patel, V., et al., *Addressing the burden of mental, neurological, and substance use disorders: key messages from Disease Control Priorities, 3rd edition.* Lancet, 2016. **387**(10028): p. 1672-85.

396. Paulus, A.T., et al., *[Inter-sectoral costs and benefits arising from mental health (disorders)].* Tijdschr Psychiatr, 2016. **58**(10): p. 688-694.

397. Payne, H. and S.D.M. Brooks, *Clinical outcomes from The BodyMind Approach™ in the treatment of patients with medically unexplained symptoms in primary health care in England: Practice-based evidence.* Arts in Psychotherapy, 2016. **47**: p. 55-65.

398. Peckham, E., et al., *Smoking Cessation Intervention for Severe Mental Ill Health Trial (SCIMITAR+): study protocol for a randomised controlled trial.* Trials, 2017. **18**(1): p. 44.

399. Pena-Longobardo, L.M. and J. Oliva-Moreno, *Economic valuation and determinants of informal care to people with Alzheimer's disease.* The European Journal of Health Economics, 2015. **16**(5): p. 507-515.

400. Pena-Longobardo, L.M. and J. Oliva-Moreno, *Caregiver burden in Alzheimer's disease patients in Spain.* Journal of Alzheimer's Disease, 2015. **43**(4): p. 1293-1302.

401. Pentaraki, A., B. Utoblo, and E.M. Kokkoli, *Cognitive remediation therapy plus standard care versus standard care for people with schizophrenia.* Cochrane Database of Systematic Reviews, 2017. **2017**(11).

402. Pentecost, C., et al., *Combining behavioural activation with physical activity promotion for adults with depression: findings of a parallel-group pilot randomised controlled trial (BAcPAc).* Trials, 2015. **16**: p. 367.

403. Pentek, M., et al., *[Health related quality of life and disease burden of patients with schizophrenia in Hungary].* Psychiatr Hung, 2012. **27**(1): p. 4-17.

404. Perez, J., et al., *Comparison of high and low intensity contact between secondary and primary care to detect people at ultra-high risk for psychosis: study protocol for a theory-based, cluster randomized controlled trial.* Trials, 2013. **14**: p. 222.

405. Peters-Scheffer N, D.R., Korzilius H, Matson J, *Cost comparison of early intensive behavioral intervention and treatment as usual for children with autism spectrum disorder in the Netherlands.* Research in Developmental Disabilities, 2012. **33**(6): p. 1763-1772.

406. Philipsen, A., et al., *Evaluation of the efficacy and effectiveness of a structured disorder tailored psychotherapy in ADHD in adults: Study protocol of a randomized controlled multicentre trial.* ADHD Attention Deficit and Hyperactivity Disorders, 2010. **2**(4): p. 203-212.

407. Phillips, L.J., et al., *Cost implications of specific and non-specific treatment for young persons at ultra high risk of developing a first episode of psychosis.* Early Intervention in Psychiatry, 2009. **3**(1): p. 28-34.

408. Pittig, A., et al., *The cost of fear: Avoidant decision making in a spider gambling task.* Journal of Anxiety Disorders, 2014. **28**(3): p. 326-334.

409. Pocklington, C., *Depression in older adults.* British Journal of Medical Practitioners, 2017. **10**(1).

410. Pohjolainen, V., et al., *Cost-effectiveness of anorexia nervosa in QALYs.* Nordic Journal of Psychiatry, 2017. **71**(1): p. 67-71.

411. Polak, A.R., et al., *Comparison of the effectiveness of trauma-focused cognitive behavioral therapy and paroxetine treatment in PTSD patients: design of a randomized controlled trial.* BMC Psychiatry, 2012. **12**: p. 166.

412. Popova, S., et al., *A literature review of cost-benefit analyses for the treatment of alcohol dependence.* Int J Environ Res Public Health, 2011. **8**(8): p. 3351-64.

413. Portrait, F.R. and B. van den Berg, *The willingness of the Dutch to pay for mental health care.* Tijdschrift voor Psychiatrie, 2016. **58**(10): p. 759-765.

414. Pouryamout, L., et al., *Economic evaluation of treatment options in patients with Alzheimer's disease: a systematic review of cost-effectiveness analyses.* Drugs, 2012. **72**(6): p. 789-802.

415. Prasad S, A.J., Steer C, Libretto SE, *Assessing the value of atomoxetine in treating children and adolescents with ADHD in the UK.* International Journal of Clinical Practice, 2009. **63**(7): p. 1031-1040.

416. Pratt, S.I., et al., *Automated telehealth for managing psychiatric instability in people with serious mental illness.* Journal of Mental Health, 2015. **24**(5): p. 261-265.

417. Prenger, R., et al., *Dealing With Missing Behavioral Endpoints in Health Promotion Research by Modeling Cognitive Parameters in Cost-Effectiveness Analyses of Behavioral Interventions: A Validation Study.* Health Econ, 2016. **25**(1): p. 24-39.

418. Prenger, R., et al., *Moving beyond a limited follow-up in cost-effectiveness analyses of behavioral interventions.* Eur J Health Econ, 2013. **14**(2): p. 297-306.

419. Prick, A.E., et al., *Process evaluation of a multicomponent dyadic intervention study with exercise and support for people with dementia and their family caregivers.* Trials, 2014. **15**: p. 401.

420. Priebe, S., et al., *Effectiveness of one-to-one volunteer support for patients with psychosis: protocol of a randomised controlled trial.* BMJ Open, 2016. **6**(8): p. e011582.

421. Purshouse, R.C., et al., *Modelling the cost-effectiveness of alcohol screening and brief interventions in primary care in England.* Alcohol Alcohol, 2013. **48**(2): p. 180-8.

422. Quentin, W., et al., *Cost-of-illness studies of dementia: a systematic review focusing on stage dependency of costs.* Acta Psychiatr Scand, 2010. **121**(4): p. 243-59.

423. Quinn, C., et al., *Self-management in early-stage dementia: a pilot randomised controlled trial of the efficacy and cost-effectiveness of a self-management group intervention (the SMART study).* Trials, 2014. **15**: p. 74.

424. Radhakrishnan, M., et al., *Cost of improving Access to Psychological Therapies (IAPT) programme: an analysis of cost of session, treatment and recovery in selected Primary Care Trusts in the East of England region.* Behav Res Ther, 2013. **51**(1): p. 37-45.

425. Rajagopalan, K., et al., *Cost-Utility Analysis of Lurasidone Versus Aripiprazole in Adults with Schizophrenia.* Pharmacoeconomics, 2016. **34**(7): p. 709-21.

426. Ramos Goñi, J.M., et al., *Cost-effectiveness of asenapine versus olanzapine for the treatment of manic episodes in patients with bipolar disorder type I.* Pharmacoeconomics - Spanish Research Articles, 2015. **12**(4): p. 123-136.

427. Ramos-Estebanez, C., et al., *Vascular cognitive impairment and dementia expenditures: 7-year inpatient cost description in community dwellers.* Postgrad Med, 2012. **124**(5): p. 91-100.

428. Ramos-Pichardo, J.D., *[Effectiveness of a nursing in-home intervention to improve independence in adls/iadls and family burden in adults with schizophrenia].* Enferm Clin, 2013. **23**(2): p. 79-80.

429. Rathod, S., et al., *Protocol for a multicentre study to assess feasibility, acceptability, effectiveness and direct costs of TRIumPH (Treatment and Recovery In PsycHosis): integrated care pathway for psychosis.* BMJ Open, 2016. **6**(12): p. e012751.

430. Ravesteijn, B., et al., *Association of cost sharing with mental health care use, involuntary commitment, and acute care.* JAMA Psychiatry, 2017. **74**(9): p. 932-939.

431. Rejas Gutiérrez, J., et al., *Economic evaluation of desvenlafaxine in the treatment of major depressive disorder in Spain.* Revista de Psiquiatria y Salud Mental, 2016. **9**(2): p. 87-96.

432. Rhind, C., et al., *Experienced Carers Helping Others (ECHO): Protocol for a pilot randomised controlled trial to examine a psycho-educational intervention for adolescents with anorexia nervosa and their carers.* European Eating Disorders Review, 2014. **22**(4): p. 267-277.

433. Rhodes, S., et al., *Cost and outcome of behavioural activation versus cognitive behaviour therapy for depression (COBRA): study protocol for a randomised controlled trial.* Trials, 2014. **15**: p. 29.

434. Ricciardi, A., V. McAllister, and P. Dazzan, *Is early intervention in psychosis effective?* Epidemiol Psichiatr Soc, 2008. **17**(3): p. 227-35.

435. Richards, D., et al., *Digital IAPT: The effectiveness & cost-effectiveness of internet-delivered interventions for depression and anxiety disorders in the Improving Access to Psychological Therapies programme: Study protocol for a randomised control trial.* BMC Psychiatry, 2018. **18**(1).

436. Richards, D.A., et al., *Clinical effectiveness of collaborative care for depression in UK primary care (CADET): cluster randomised controlled trial.* Bmj, 2013. **347**: p. f4913.

437. Richter, C., et al., *Effectiveness of dialectical behavior therapy (DBT) in an outpatient clinic for borderline personality disorders - Impact of medication use and treatment costs.* Psychiatrische Praxis, 2014. **41**(3): p. 148-152.

438. Richter, C., et al., *Inpatient and outpatient DBT options for patients with borderline personality disorder – Results from a German survey.* Verhaltenstherapie, 2014. **24**(4): p. 265-271.

439. Riedel-Heller, S.G., *[Research in social psychiatry - addressing future challenges of health- and social systems].* Neuropsychiatr, 2009. **23**(4): p. 249-52.

440. Rihmer, Z. and A. Nemeth, *[Correlation between treatment of depression and suicide mortality in Hungary -- focus on the effects of the 2007 healthcare reform].* Neuropsychopharmacol Hung, 2014. **16**(4): p. 195-204.

441. Riihimäki, K., A. Heiska-Johansson, and E. Ketola, *Case-mix tool, costs and effectiveness in improving primary care mental health and substance abuse services.* Nordic Journal of Psychiatry, 2018. **72**(2): p. 109-111.

442. Rive, B., et al., *Cost effectiveness of memantine in Alzheimer's disease in the UK.* J Med Econ, 2010. **13**(2): p. 371-80.

443. Rivera, B., B. Casal, and L. Currais, *Provision de cuidados informales y enfermedad de Alzheimer: Valoracion economica y estudio de la variabilidad del tiempo. (Informal Care Provision and Alzheimer's Disease: Economic Valuation and Study of the Variability of Time. With English summary.).* Hacienda Publica Espanola/Revista de Economia Publica, 2009(189): p. 107-130.

444. Roca, M., et al., *Economic crisis and mental health in Spain.* The Lancet, 2013. **382**(9909): p. 1977-1978.

445. Rodriguez-Jimenez, R., et al., *Clinical usefulness and economic implications of continuation/maintenance electroconvulsive therapy in a Spanish National Health System public hospital: A case series.* Rev Psiquiatr Salud Ment, 2015. **8**(2): p. 75-82.

446. Rodriguez-Jimenez, R., et al., *Maintenance electroconvulsive therapy cost-effectiveness and patient/family satisfaction.* Journal of ECT, 2015. **31**(4): p. 279.

447. Romijn, G., et al., *Cost-effectiveness of blended vs. face-to-face cognitive behavioural therapy for severe anxiety disorders: study protocol of a randomized controlled trial.* BMC Psychiatry, 2015. **15**: p. 311.

448. Rona, R.J., et al., *Post-deployment screening for mental disorders and tailored advice about help-seeking in the UK military: a cluster randomised controlled trial.* The Lancet, 2017. **389**(10077): p. 1410-1423.

449. Rong, P., et al., *Effect of transcutaneous auricular vagus nerve stimulation on major depressive disorder: A nonrandomized controlled pilot study.* Journal of Affective Disorders, 2016. **195**: p. 172-179.

450. Rubio-Terres, C., D. Rubio-Rodriguez, and E. Baca-Baldomero, *Cost analysis of the adverse reactions of bipolar disorder treatment with aripiprazole and olanzapine in Spain.* Actas Espanolas de Psiquiatria, 2014. **42**(5): p. 242-249.

451. Rubio-Valera, M., et al., *Cost-effectiveness of active monitoring versus antidepressants for major depression in primary health care: a 12-month non-randomized controlled trial (INFAP study).* BMC Psychiatry, 2015. **15**: p. 63.

452. Ruiz-Rodriguez, P., et al., *A systematic review and critique of the economic impact and burden of common mental disorders in Spain.* Ansiedad y Estres, 2017. **23**(2-3): p. 118-123.

453. Rummel-Kluge, C., G. Pitschel-Walz, and W. Kissling, *Psychoeducation in anxiety disorders: Results of a survey of all psychiatric institutions in Germany, Austria and Switzerland.* Psychiatry Research, 2009. **169**(2): p. 180-182.

454. Rüther, T., et al., *Smoking Cessation Program for Inpatients with Substance Use Disorder: A Quasi-Randomized Controlled Trial of Feasibility and Efficacy.* European Addiction Research, 2016. **22**(5): p. 268-276.

455. Sabes-Figuera, R., et al., *The local burden of emotional disorders. An analysis based on a large health survey in Catalonia (Spain).* Gaceta Sanitaria, 2012. **26**(1): p. 24-29.

456. Sabes-Figuera, R., et al., *Developing a tool for collecting and costing activity data on psychiatric inpatient wards.* Epidemiology and Psychiatric Sciences, 2012. **21**(4): p. 393-399.

457. Salize, H.J. and C. Roth-Sackenheim, *[Economic competition in the market to improve outpatient psychiatric care].* Psychiatr Prax, 2009. **36**(3): p. 106-9.

458. Salloum, A., et al., *Stepped care versus standard trauma-focused cognitive behavioral therapy for young children.* Journal of child psychology and psychiatry, and allied disciplines, 2016. **57**(5): p. 614-622.

459. Salvador-Carulla, L. and S. Symonds, *Health services use and costs in people with intellectual disability: Building a context knowledge base for evidence-informed policy.* Current Opinion in Psychiatry, 2016. **29**(2): p. 89-94.

460. Sampson, C.J., et al., *Cost implications of treatment non-completion in a forensic personality disorder service.* Criminal Behaviour and Mental Health, 2013. **23**(5): p. 321-335.

461. Sanatinia, R., et al., *Impact of personality status on the outcomes and cost of cognitive-behavioural therapy for health anxiety.* British Journal of Psychiatry, 2016. **209**(3): p. 244-250.

462. Sanches, S.A., et al., *Cost effectiveness and budgetary impact of the Boston University approach to Psychiatric Rehabilitation for societal participation in people with severe mental illness: a randomised controlled trial protocol.* BMC Psychiatry, 2015. **15**: p. 217.

463. Sarkar, S.N., et al., *Unheard voices: Outcomes of tertiary care for treatment-refractory psychosis.* Psychiatrist, 2014. **38**(2): p. 71-74.

464. Sawyer, L., et al., *Cost-effectiveness of asenapine in the treatment of bipolar i disorder patients with mixed episodes.* Journal of Medical Economics, 2014. **17**(7): p. 508-519.

465. Schaller, S., et al., *The main cost drivers in dementia: a systematic review.* Int J Geriatr Psychiatry, 2015. **30**(2): p. 111-29.

466. Schawo, S., et al., *Probabilistic Markov Model Estimating Cost Effectiveness of Methylphenidate Osmotic-Release Oral System Versus Immediate-Release Methylphenidate in Children and Adolescents: Which Information is Needed?* Pharmacoeconomics, 2015. **33**(5): p. 489-509.

467. Schawo, S.J., et al., *Framework for modelling the cost-effectiveness of systemic interventions aimed to reduce youth delinquency.* Journal of Mental Health Policy and Economics, 2012. **15**(4): p. 187-196+199+202.

468. Schilling, C.J., B.C. Storm, and M.C. Anderson, *Examining the costs and benefits of inhibition in memory retrieval.* Cognition, 2014. **133**(2): p. 358-370.

469. Schlander, M., *The pharmaceutical economics of child psychiatric drug treatment.* Curr Pharm Des, 2010. **16**(22): p. 2443-61.

470. Schlander, M., G. Trott, and O. Schwarz, *The health economics of attention deficit hyperactivity disorder in Germany. Part 1: health care utilization and cost of illness.* Der Nervenarzt, 2010. **81**(3): p. 289-300.

471. Schlander, M., G.E. Trott, and O. Schwarz, *The health economics of attention deficit hyperactivity disorder in Germany. Part 1: Health care utilization and cost of illness.* Nervenarzt, 2010. **81**(3): p. 289-300.

472. Schlander, M., G.E. Trott, and O. Schwarz, *The health economics of attention deficit hyperactivity disorder in Germany. Part 2: Therapeutic options and their cost-effectiveness.* Nervenarzt, 2010. **81**(3): p. 301-314.

473. Schmidt, U., et al., *Do adolescents with eating disorder not otherwise specified or full-syndrome bulimia nervosa differ in clinical severity, comorbidity, risk factors, treatment outcome or cost?* International Journal of Eating Disorders, 2008. **41**(6): p. 498-504.

474. Schoevers, R.A., et al., *[Costs and benefits of psychiatry].* Tijdschr Psychiatr, 2016. **58**(10): p. 680-682.

475. Schröder, A., et al., *Long-term economic evaluation of cognitive-behavioural group treatment versus enhanced usual care for functional somatic syndromes.* Journal of Psychosomatic Research, 2017. **94**: p. 73-81.

476. Shah, A., et al., *Healthcare utilization and costs associated with treatment for opioid dependence.* Journal of Medical Economics, 2018. **21**(4): p. 406-415.

477. Shah, S., et al., *Cost–utility analysis of methylphenidate and amphetamine/dexamphetamine in adults with attention-deficit hyperactivity disorder.* Journal of Pharmaceutical Health Services Research, 2017. **8**(2): p. 101-106.

478. Shahab, L., L.S. Brose, and R. West, *Novel delivery systems for nicotine replacement therapy as an aid to smoking cessation and for harm reduction: Rationale, and evidence for advantages over existing systems.* CNS Drugs, 2013. **27**(12): p. 1007-1019.

479. Shearer, J., et al., *A cost-effectiveness analysis of modafinil therapy for psychostimulant dependence.* Drug Alcohol Rev, 2010. **29**(3): p. 235-42.

480. Sherman, K.J., et al., *Effectiveness of therapeutic massage for generalized anxiety disorder: A randomized controlled trial.* Depression and Anxiety, 2010. **27**(5): p. 441-450.

481. Shield, K.D., et al., *The potential impact of increased treatment rates for alcohol dependence in the United Kingdom in 2004.* BMC Health Serv Res, 2014. **14**: p. 53.

482. Sicras-Mainar, A. and R. Navarro-Artieda, *Use of antidepressants in the treatment of major depressive disorder in primary care during a period of economic crisis.* Neuropsychiatric Disease and Treatment, 2016. **12**: p. 29-40.

483. Sikorski, C., et al., *[Computer-aided cognitive behavioral therapy for depression].* Psychiatr Prax, 2011. **38**(2): p. 61-8.

484. Simkiss, D.E., et al., *Measuring the impact and costs of a universal group based parenting programme: protocol and implementation of a trial.* BMC public health, 2010. **10**: p. 364.

485. Simon, E., C.D. Dirksen, and S.M. Bögels, *An explorative cost-effectiveness analysis of school-based screening for child anxiety using a decision analytic model.* European Child and Adolescent Psychiatry, 2013. **22**(10): p. 619-630.

486. Simpson, W., et al., *The Effectiveness of a Community Intensive Therapy Team on Young People's Mental Health Outcomes.* Child and Adolescent Mental Health, 2010. **15**(4): p. 217-223.

487. Singh, I., S. Ramakrishna, and K. Williamson, *The Rapid Assessment Interface and Discharge service and its implications for patients with dementia.* Clin Interv Aging, 2013. **8**: p. 1101-8.

488. Skapinakis, P., et al., *A systematic review of the clinical effectiveness and cost-effectiveness of pharmacological and psychological interventions for the management of obsessive–compulsive disorder in children/adolescents and adults.* Health Technology Assessment, 2016. **20**(43).

489. Smit F, L.J., Riper H, Majo MC, Boon B, Blankers M, *Modeling the cost-effectiveness of health care systems for alcohol use disorders: how implementation of eHealth interventions improves.* Journal of Medical Internet Research, 2011. **13**(3): p. e56.

490. Smith, B.J., et al., *The identification in primary care of patients who have been repeatedly referred to hospital for medically unexplained symptoms: A pilot study.* Journal of Psychosomatic Research, 2009. **67**(3): p. 207-211.

491. Smits, F., et al., *An epidemiological approach to depression prevention in old age.* American Journal of Geriatric Psychiatry, 2008. **16**(6): p. 444-453.

492. Snyder, A., et al., *Do high fidelity wraparound services for youth with serious emotional disturbances save money in the long-term?* Journal of Mental Health Policy and Economics, 2017. **20**(4): p. 167-175.

493. Soeteman, D.I., R. Verheul, and J.J. Busschbach, *The burden of disease in personality disorders: diagnosis-specific quality of life.* J Pers Disord, 2008. **22**(3): p. 259-68.

494. Soeteman, D.I., et al., *Cost-effectiveness of psychotherapy for cluster B personality disorders.* Br J Psychiatry, 2010. **196**(5): p. 396-403.

495. Soeteman, D.I., et al., *Cost-effectiveness of psychotherapy for cluster C personality disorders: a decision-analytic model in the Netherlands.* J Clin Psychiatry, 2011. **72**(1): p. 51-9.

496. Sohn, M., et al., *A cost-effectiveness analysis of off-label atypical antipsychotic treatment in children and adolescents with ADHD who have failed stimulant therapy.* ADHD Attention Deficit and Hyperactivity Disorders, 2016. **8**(3): p. 149-158.

497. Soler, J., et al., *Dialectical behaviour therapy skills training compared to standard group therapy in borderline personality disorder: A 3-month randomised controlled clinical trial.* Behaviour Research and Therapy, 2009. **47**(5): p. 353-358.

498. Solomon, D., J. Adams, and N. Graves, *Economic evaluation of St. John's wort (Hypericum perforatum) for the treatment of mild to moderate depression.* Journal of Affective Disorders, 2013. **148**(2-3): p. 228-234.

499. Sonntag, M., H.H. König, and A. Konnopka, *The estimation of utility weights in cost-utility analysis for mental disorders: A systematic review.* PharmacoEconomics, 2013. **31**(12): p. 1131-1154.

500. Soravia, L.M., et al., *Symptom-triggered detoxification using the alcohol-withdrawal-scale reduces risks and healthcare costs.* Alcohol and Alcoholism, 2018. **53**(1): p. 71-77.

501. Soto-Gordoa, M., et al., *Projecting Burden of Dementia in Spain, 2010-2050: Impact of Modifying Risk Factors.* J Alzheimers Dis, 2015. **48**(3): p. 721-30.

502. Spencer, L., et al., *A web-based intervention for carers of individuals with anorexia nervosa (We Can): Trial protocol of a randomised controlled trial investigating the effectiveness of different levels of support.* Internet Interventions, 2018.

503. Sperling, W., U. Reulbach, and J. Kornhuber, *Clinical benefits and cost effectiveness of vagus nerve stimulation in a long-term treatment of patients with major depression.* Pharmacopsychiatry, 2009. **42**(3): p. 85-8.

504. Stapleton, J.A., et al., *Varenicline in the routine treatment of tobacco dependence: A pre-post comparison with nicotine replacement therapy and an evaluation in those with mental illness.* Addiction, 2008. **103**(1): p. 146-154.

505. Stensland, M.D., J.F. Schultz, and J.R. Frytak, *Depression diagnoses following the identification of bipolar disorder: Costly incongruent diagnoses.* BMC Psychiatry, 2010. **10**.

506. Stensland, M.D., et al., *Costs associated with attempted suicide among individuals with bipolar disorder.* Journal of Mental Health Policy and Economics, 2010. **13**(2): p. 87-92+95+98.

507. Stephen, C., H. Sultan, and E. Frew, *Valuing telecare using willingness to pay from the perspective of carers for people with dementia: a pilot study from the West Midlands.* J Telemed Telecare, 2014. **20**(3): p. 141-6.

508. Stevenson, M.D., et al., *Group cognitive behavioural therapy for postnatal depression: a systematic review of clinical effectiveness, cost-effectiveness and value of information analyses.* Health Technol Assess, 2010. **14**(44): p. 1-107, iii-iv.

509. Stuhldreher, N., et al., *Cost-of-illness studies and cost-effectiveness analyses in eating disorders: a systematic review.* Int J Eat Disord, 2012. **45**(4): p. 476-91.

510. Stuttard, L., et al., *A preliminary investigation into the effectiveness of a group-delivered sleep management intervention for parents of children with intellectual disabilities.* Journal of Intellectual Disabilities, 2015. **19**(4): p. 342-355.

511. Stuttard, L., et al., *Riding the Rapids: Living with autism or disability-An evaluation of a parenting support intervention for parents of disabled children.* Research in Developmental Disabilities, 2014. **35**(10): p. 2371-2383.

512. Suijkerbuijk, A.W., et al., *[Cost-effectiveness of addiction care].* Tijdschr Psychiatr, 2015. **57**(7): p. 498-507.

513. Sung, H.C., et al., *A group music intervention using percussion instruments with familiar music to reduce anxiety and agitation of institutionalized older adults with dementia.* International Journal of Geriatric Psychiatry, 2012. **27**(6): p. 621-627.

514. Taneja, C., et al., *Cost-effectiveness of adjunctive therapy with atypical antipsychotics for acute treatment of major depressive disorder.* Annals of Pharmacotherapy, 2012. **46**(5): p. 642-649.

515. Tariq, L., et al., *Cost-effectiveness of an opportunistic screening programme and brief intervention for excessive alcohol use in primary care.* PLoS One, 2009. **4**(5): p. e5696.

516. Taylor, D., et al., *Risperidone long-acting injection: A 6-year mirror-image study of healthcare resource use.* Acta Psychiatrica Scandinavica, 2009. **120**(2): p. 97-101.

517. Taylor, M., et al., *Cost effectiveness of interventions to reduce relapse to smoking following smoking cessation.* Addiction, 2011. **106**(10): p. 1819-26.

518. Thiart, H., et al., *Internet-Based Cognitive Behavioral Therapy for Insomnia: A Health Economic Evaluation.* Sleep, 2016. **39**(10): p. 1769-1778.

519. Thiart, H., et al., *Log in and breathe out: Efficacy and cost-effectiveness of an online sleep training for teachers affected by work-related strain - study protocol for a randomized controlled trial.* Trials, 2013. **14**(1).

520. Thomas, C.L., et al., *Effectiveness and cost-effectiveness of a telehealth intervention to support the management of long-term conditions: study protocol for two linked randomized controlled trials.* Trials, 2014. **15**: p. 36.

521. Thunnissen, M., et al., *A randomized clinical trial on the effectiveness of a reintegration training program versus booster sessions after short-term inpatient psychotherapy.* Journal of personality disorders, 2008. **22**(5): p. 483-495.

522. Tilford, J.M., et al., *Treatment for Sleep Problems in Children with Autism and Caregiver Spillover Effects.* Journal of Autism and Developmental Disorders, 2015. **45**(11): p. 3613-3623.

523. Tomlinson, M., et al., *Setting priorities for global mental health research.* Bulletin of the World Health Organization, 2009. **87**(6): p. 438-446.

524. Trapero-Bertran, M., *[Economic evaluation of smoking cessation interventions: have we overlooked something].* Arch Bronconeumol, 2009. **45**(5): p. 209-11.

525. Trautmann, S., J. Rehm, and H.U. Wittchen, *The economic costs of mental disorders: Do our societies react appropriately to the burden of mental disorders?* EMBO Reports, 2016. **17**(9): p. 1245-1249.

526. Treur M, B.E., Bobes J, Canas F, Salvador L, Gonzalez B, Heeg B, *The cost-effectiveness of paliperidone extended release in Spain.* Journal of Medical Economics, 2012. **15**(Supplement 1): p. 26-34.

527. Trevillion, K., et al., *Depression: An exploratory parallel-group randomised controlled trial of Antenatal guided self help for WomeN (DAWN): Study protocol for a randomised controlled trial.* Trials, 2016. **17**(1).

528. Turri, M.G. and L. Andreatta, *Does psychoanalytic psychotherapy offset use of mental health services and related costs in severe borderline personality disorder? - A case study.* Psychoanalytic Psychotherapy, 2014. **28**(2): p. 139-158.

529. Twomey, C., et al., *Utility of the health of the nation outcome scales (HoNOS) in predicting mental health service costs for patients with common mental health problems: Historical cohort study.* PLoS ONE, 2016. **11**(11).

530. Tyrer, P., et al., *CHAMP: Cognitive behaviour therapy for health anxiety in medical patients, a randomised controlled trial.* BMC Psychiatry, 2011. **11**.

531. Tyrer, P., et al., *Risperidone, haloperidol, and placebo in the treatment of aggressive challenging behaviour in patients with intellectual disability: a randomised controlled trial.* The Lancet, 2008. **371**(9606): p. 57-63.

532. Vallejo-Torres, L., et al., *Cost-effectiveness of electroconvulsive therapy compared to repetitive transcranial magnetic stimulation for treatment-resistant severe depression: a decision model.* Psychol Med, 2015. **45**(7): p. 1459-70.

533. Valmaggia, L.R., et al., *Economic impact of early intervention in people at high risk of psychosis.* Psychol Med, 2009. **39**(10): p. 1617-26.

534. Van Agthoven, M., et al., *Cost-effectiveness in Dutch mental health care: Future because of ROM?* Tijdschrift voor Psychiatrie, 2015. **57**(9): p. 672-679.

535. van Asselt, A.D., et al., *How to deal with cost differences at baseline.* Pharmacoeconomics, 2009. **27**(6): p. 519-28.

536. van den Berg, M., et al., *Cost-effectiveness of opportunistic screening and minimal contact psychotherapy to prevent depression in primary care patients.* PLoS One, 2011. **6**(8): p. e22884.

537. Van Der Gaag, M., *The efficacy of CBT for severe mental illness and the challenge of dissemination in routine care.* World Psychiatry, 2014. **13**(3): p. 257-258.

538. van der Gaag, M., et al., *[Prognostic modelling and proactive intervention in psychosis: efficacy and cost-effectiveness].* Tijdschr Psychiatr, 2016. **58**(10): p. 695-699.

539. van der Schans, J., et al., *Cost-effectiveness of extended-release methylphenidate in children and adolescents with attention-deficit/hyperactivity disorder sub-optimally treated with immediate release methylphenidate.* PLoS One, 2015. **10**(5): p. e0127237.

540. van Dongen, J.M., et al., *A cost-effectiveness and return-on-investment analysis of a worksite vitality intervention among older hospital workers: results of a randomized controlled trial.* J Occup Environ Med, 2013. **55**(3): p. 337-46.

541. van Dongen, J.M., et al., *Long-Term Cost-Effectiveness and Return-on-Investment of a Mindfulness-Based Worksite Intervention: Results of a Randomized Controlled Trial.* J Occup Environ Med, 2016. **58**(6): p. 550-60.

542. Van Oostrom, S.H., et al., *Cost-effectiveness of a workplace intervention for sick-listed employees with common mental disorders: Design of a randomized controlled trial.* BMC Public Health, 2008. **8**.

543. van Orden M, H.T., Haffmans J, Spinhoven P, Hoencamp E, *Collaborative mental health care versus care as usual in a primary care setting: a randomized controlled trial.* Psychiatric Services, 2009. **60**(1): p. 74-79.

544. van Steenbergen-Weijenburg, K.M., et al., *Cost-effectiveness of collaborative care for the treatment of major depressive disorder in primary care. A systematic review.* BMC Health Serv Res, 2010. **10**: p. 19.

545. van Veen, M., et al., *Cost effectiveness of interpersonal community psychiatric treatment for people with long-term severe non-psychotic mental disorders: protocol of a multi-centre randomized controlled trial.* BMC Psychiatry, 2015. **15**: p. 100.

546. Vanoli, A., et al., *Adequacy of venlafaxine dose prescribing in major depression and hospital resources implications.* Journal of Psychopharmacology, 2008. **22**(4): p. 434-440.

547. Vara, M.D., et al., *Efficacy and cost-effectiveness of a blended cognitive behavioral therapy for depression in Spanish primary health care: Study protocol for a randomised non-inferiority trial.* BMC Psychiatry, 2018. **18**(1).

548. Vataire, A.L., et al., *Core discrete event simulation model for the evaluation of health care technologies in major depressive disorder.* Value in Health, 2014. **17**(2): p. 183-195.

549. Vazquez-Mourelle, R., et al., *[Efficiency of a pharmaceutical care program for long-acting parenteral antipsychotics in the health area of Santiago de Compostela].* Gac Sanit, 2016. **30**(1): p. 73-6.

550. Vera-Llonch, M., et al., *Cost-effectiveness of pregabalin versus venlafaxine in the treatment of generalized anxiety disorder: findings from a Spanish perspective.* Eur J Health Econ, 2010. **11**(1): p. 35-44.

551. Vermeiren, R.M. and J. van der Meer, *[Child psychiatry: limited research, evidence for cost-effectiveness of treatment].* Tijdschr Psychiatr, 2016. **58**(10): p. 728-732.

552. Veronika, M., G. Tamás, and V. Enikő, *The cognitive paradigm in the rehabilitation of schizophrenia – focusing on cognitive remediation.* Neuropsychopharmacologia Hungarica, 2015. **17**(3): p. 129-140.

553. Visser, M.S., et al., *The Cost-Effectiveness of Cognitive-Behavioral Group Training for Patients with Unexplained Physical Symptoms.* Value Health, 2015. **18**(5): p. 570-7.

554. Vitiello, B., *Treatment of adolescent depression: What we have come to know.* Depression and Anxiety, 2009. **26**(5): p. 393-395.

555. Wammes, J.J.G., et al., *Characteristics and healthcare utilization of patients with highest costs of care.* Nederlands Tijdschrift voor Geneeskunde, 2017. **161**(49).

556. Warmerdam, L., et al., *Internet-based treatment for adults with depressive symptoms: Randomized controlled trial.* Journal of Medical Internet Research, 2008. **10**(4).

557. Watzke, B., et al., *Effectiveness and cost-effectiveness of telephone-based cognitive-behavioural therapy in primary care: study protocol of TIDe - telephone intervention for depression.* BMC Psychiatry, 2017. **17**(1): p. 263.

558. Watzke, B., et al., *Effectiveness and cost-effectiveness of a guideline-based stepped care model for patients with depression: Study protocol of a cluster-randomized controlled trial in routine care.* BMC Psychiatry Vol 14 2014, ArtID 230, 2014. **14**.

559. Weck, F., et al., *Effectiveness of cognitive-behavioral group therapy for patients with hypochondriasis (health anxiety).* Journal of Anxiety Disorders, 2015. **30**: p. 1-7.

560. Weisel, K.K., et al., *Efficacy and cost-effectiveness of guided and unguided internet- and mobile-based indicated transdiagnostic prevention of depression and anxiety (ICare Prevent): A three-armed randomized controlled trial in four European countries.* Internet Interventions, 2018.

561. Wetzelaer, P., et al., *Cost-effectiveness of psychotherapy for personality disorders. A systematic review on economic evaluation studies.* Tijdschrift voor Psychiatrie, 2016. **58**(10): p. 717-727.

562. Whitney, J., et al., *A practical comparison of two types of family intervention: An exploratory RCT of family day workshops and individual family work as a supplement to inpatient care for adults with anorexia nervosa.* European Eating Disorders Review, 2012. **20**(2): p. 142-150.

563. Wiersma, J.E., et al., *Treatment of chronically depressed patients: A multisite randomized controlled trial testing the effectiveness of 'Cognitive Behavioral Analysis System of Psychotherapy' (CBASP) for chronic depressions versus usual secondary care.* BMC Psychiatry, 2008. **8**.

564. Wiertsema, S.H., et al., *Evaluation of a new Transmural Trauma Care Model (TTCM) for the rehabilitation of trauma patients: a study protocol.* BMC Health Serv Res, 2017. **17**(1): p. 99.

565. Wildman, J., et al., *Economic evaluation of integrated new technologies for health and social care: Suggestions for policy makers, users and evaluators.* Soc Sci Med, 2016. **169**: p. 141-148.

566. Williams, A.D., et al., *The effectiveness of internet cognitive behaviour therapy (iCBT) for social anxiety disorder across two routine practice pathways.* Internet Interventions, 2014. **1**(4): p. 225-229.

567. Williams, C., et al., *Community-based group guided self-help intervention for low mood and stress: Randomised controlled trial.* British Journal of Psychiatry, 2018. **212**(2): p. 88-95.

568. Wise, J., *Short course of cognitive behavioural therapy may reduce health anxiety, say researchers.* BMJ (Online), 2013. **347**(7930).

569. Wittchen, H.U., et al., *The size and burden of mental disorders and other disorders of the brain in Europe 2010.* Eur Neuropsychopharmacol, 2011. **21**(9): p. 655-79.

570. Wolff, J., et al., *Cost drivers of inpatient mental health care: A systematic review.* Epidemiology and Psychiatric Sciences, 2015. **24**(1): p. 78-89.

571. Wolff, J., et al., *Determinants of per diem hospital costs in mental health.* PLoS ONE, 2016. **11**(3).

572. Woods, R.T., et al., *Reminiscence groups for people with dementia and their family carers: Pragmatic eight-centre randomised trial of joint reminiscence and maintenance versus usual treatment: A protocol.* Trials, 2009. **10**.

573. Wright, B., et al., *Computerised cognitive-behavioural therapy for depression in adolescents: Feasibility results and 4-month outcomes of a UK randomised controlled trial.* BMJ Open, 2017. **7**(1).

574. Wu, E., et al., *Comparison of treatment persistence, hospital utilization and costs among major depressive disorder geriatric patients treated with escitalopram versus other SSRI/SNRI antidepressants.* Current Medical Research and Opinion, 2008. **24**(10): p. 2805-2813.

575. Wu, E., et al., *Comparison of escitalopram versus citalopram for the treatment of major depressive disorder in a geriatric population.* Current Medical Research and Opinion, 2008. **24**(9): p. 2587-2595.

576. Wu, E.Q., et al., *Healthcare utilization and costs incurred by patients with major depression after being switched from escitalopram to another SSRI for non-medical reasons.* Journal of Medical Economics, 2010. **13**(2): p. 314-323.

577. Wubbeler, M., et al., *[Outpatient Care of People with Dementia within Residential Communities in Germany--Care Potential and Cost].* Gesundheitswesen, 2015. **77**(11): p. 839-44.

578. Wunner, C., et al., *Effectiveness of a psychosomatic day hospital treatment for the elderly: A naturalistic longitudinal study with waiting time before treatment as control condition.* Journal of Psychosomatic Research, 2014. **76**(2): p. 121-126.

579. Wunsch, E.-M., et al., *How expensive is it really going to be? Cost-effectiveness analysis of psychotherapy for anxiety and mood disorders in Germany.* Psychologische Rundschau, 2013. **64**(2): p. 75-93.

580. Young, A., et al., *Cost-utility evaluation of vortioxetine in patients with major depressive disorder experiencing inadequate response to alternative antidepressants in the United Kingdom.* Journal of Affective Disorders, 2017. **218**: p. 291-298.

581. Yu, A.P., et al., *Economic consequence of switching to citalopram after its generic entry for adult patients with major depressive disorder (MDD) treated with escitalopram: A 6-month retrospective study.* Journal of Medical Economics, 2010. **13**(4): p. 599-609.

582. Zeidler, J., et al., *Calculation of disease-related costs in claims data analyses with the example of attention-deficit hyperactivity disorder: Comparison of methods.* Bundesgesundheitsblatt - Gesundheitsforschung - Gesundheitsschutz, 2013. **56**(3): p. 430-438.

583. Zeidler, J., et al., *Cost Effectiveness of Paliperidone Palmitate for the Treatment of Schizophrenia in Germany: Erratum.* Applied Health Economics and Health Policy, 2013. **11**(6): p. 689-689.

584. Zimmermann, G., Y. De Roten, and J.N. Despland, *Efficacy, cost-effectiveness and appropriateness of psychotherapy: A review.* Schweizer Archiv fur Neurologie und Psychiatrie, 2008. **159**(3): p. 119-126.

585. Zimovetz, E.A., et al., *A Cost-Utility Analysis of Lisdexamfetamine Versus Atomoxetine in the Treatment of Children and Adolescents with Attention-Deficit/Hyperactivity Disorder and Inadequate Response to Methylphenidate.* CNS Drugs, 2016. **30**(10): p. 985-996.

586. Zimovetz, E.A., et al., *A Cost-Effectiveness Analysis of Lisdexamfetamine Dimesylate in the Treatment of Adults with Attention-Deficit/Hyperactivity Disorder in the UK.* European Journal of Health Economics, 2018. **19**(1): p. 21-35.

587. Zoun, M.H., et al., *Effectiveness and cost-effectiveness of a self-management training for patients with chronic and treatment resistant anxiety or depressive disorders: design of a multicenter randomized controlled trial.* BMC Psychiatry, 2016. **16**: p. 216.

## Appendix 4: Identified and screened resource use questionnaires

| **Name** | **Acronym** | **Reference** |
| --- | --- | --- |
| Client Service Receipt Inventory | CSRI | [1] |
| Client Sociodemographic and Service Receipt Inventory | CSSRI | [2] |
| Secure Facilities Service Use Schedule | SF-SUS | [3] |
| Child and Adolescent Service Use Schedule | CA-SUS | [4] |
| Treatment Inventory of Costs in Patients with Psychiatric Disorders | TiC-P | [5] |
| Fragebogen zur Inanspruchnahme medizinischer Leistungen bei Älteren | FIMA | [6] |
| Fragebogen zur Inanspruchnahme medizinischer Leistungen bei psychischen Erkrankungen | FIMPsy | [7] |
| World Health Organization Health and Work Performance Questionnaire | HPQ | [8] |
| Resource Utilization in Dementia | RUD | [9] |
| Health Care Utilization Questionnaire | HCU-Q | [10] |
| Children’s Service Interview | CSI | [11] |
| Items for a Standardized Resource Use Measure | ISRUM | [12] |

1. Beecham J.K., K.M.R.J., *Costing psychiatric interventions.*, in *Measuring mental health needs*, B.C. Thornicroft G., Wing J. , Editor. 1992, Gaskell: London.

2. Chisholm, D., et al., *Client Socio-Demographic and Service Receipt Inventory--European Version: development of an instrument for international research. EPSILON Study 5. European Psychiatric Services: Inputs Linked to Outcome Domains and Needs.* Br J Psychiatry Suppl, 2000(39): p. s28-33.

3. Barrett, B. and S. Byford, *Collecting service use data for economic evaluation in DSPD populations: development of the Secure Facilities Service Use Schedule.* Br J Psychiatry Suppl, 2007. **49**: p. s75-8.

4. Byford S., H.R., Torgerson D., Kerfoot M., Dyer E., Harrington V., Woodham a., Gill J., McNiven F., *Cost-effectiveness analysis of a home-based social work intervention for children and adolescents who have deliberately poisoned themselves. Results of a randomised controlled trial.* Br J Psychiatry 1999. **174**: p. 56-62.

5. Hakkaart-Van Roijen L., V.S.A., Donker M., Arendts L., *Manual Trimbos iMTA Questionnaire for Costs Associated With Psychiatric Illness (TIC-P) [in Dutch]. Rotterdam, the Netherlands: Erasmus University.* 2002.

6. Seidl, H., et al., *[FIMA--questionnaire for health-related resource use in an elderly population: development and pilot study].* Gesundheitswesen, 2015. **77**(1): p. 46-52.

7. Grupp, H., et al., *[FIMPsy - Questionnaire for the Assessment of Medical and non Medical Resource Utilisation in Mental Disorders: Development and Application].* Psychiatr Prax, 2018. **45**(2): p. 87-94.

8. Kessler, R.C., et al., *The World Health Organization Health and Work Performance Questionnaire (HPQ).* J Occup Environ Med, 2003. **45**(2): p. 156-74.

9. Wimo, A. and G. Nordberg, *Validity and reliability of assessments of time. Comparisons of direct observations and estimates of time by the use of the resource utilization in dementia (RUD)-instrument.* Arch Gerontol Geriatr, 2007. **44**(1): p. 71-81.

10. Weiss F. D., M.R., Rief W., Kleinstäuber, M. , *HCU-Q: Entwicklung eines Fragebogens zur Erfassung der Inanspruchnahme medizinischer Gesundheitsleistungen.* Verhaltenstherapie, 2018. **28** ((1)): p. 15-24.

11. Ford, T., et al., *The children's services interview: validity and reliability.* Soc Psychiatry Psychiatr Epidemiol, 2007. **42**(1): p. 36-49.

12. Thorn, J.C., et al., *Core Items for a Standardized Resource Use Measure: Expert Delphi Consensus Survey.* Value Health, 2018. **21**(6): p. 640-649.
